# BBX maintains mammalian brain development by repressing p53-mediated p21 expression

**DOI:** 10.1038/s44319-026-00949-4

**Published:** 2026-09-25

**Authors:** Youngik Yoon, Gwang Yeong Kim, Woo Young Lim, Saeah Hwang, Solji Lee, Dongrok Yoon, Samuel J Pleasure, Keejung Yoon

**Affiliations:** 1 Department of Biopharmaceutical Convergence, Sungkyunkwan University, Suwon, South Korea; 2 Department of Integrative Biotechnology, Sungkyunkwan University, Suwon, South Korea; 3 Department of Neurology, University of California San Francisco, San Francisco, CA USA

**Keywords:** Chromatin, Transcription & Genomics, Neuroscience

## Abstract

Brain development requires coordinated regulation of neural stem cell (NSC) proliferation, differentiation, and neuronal migration. Here, we identify bobby sox homolog (BBX), a nuclear HMG box domain-containing protein enriched in mouse embryonic cortical germinal zones, as a regulator of corticogenesis. In the embryonic cortex, BBX depletion reduces SOX2-expressing NSCs, promotes premature cell-cycle exit, disrupts cortical cell positioning, causes abnormal clustering of newborn neurons, and impairs migratory neuron morphology. RNA-seq and public ChIP-seq analyses, together with biochemical assays, show that BBX interacts with p53 and suppresses p53-dependent p21 expression. Consistent with activation of the p21 pathway, BBX knockdown increases premature cell-cycle exit and senescence-associated features, including γH2AX accumulation and senescence-associated gene expression. Co-depletion of p21 rescues BBX knockdown-induced defects in NSC maintenance, neuronal distribution, and morphology. In line with this, depletion of retinoblastoma protein (RB), a major mediator of p21-dependent senescence, restores abnormal cortical cell distribution. These findings indicate that BBX maintains normal cortical development by restraining the p53–p21–RB axis, thereby preventing premature cell-cycle exit and senescence in developing neural cells.

## Synopsis

BBX promotes mammalian cortical development by maintaining neural stem/progenitor cells and supporting newborn neuron migration. By restraining p53-mediated p21 expression, BBX prevents premature cell-cycle exit and senescence-associated changes, safeguarding normal brain development.

- BBX depletion in the mouse embryonic brain promotes premature cell-cycle exit in the germinal zones and impairs newborn neuron migration and morphology.
- BBX associates with p53 and represses p53-dependent p21 expression.
- Suppression of p53 activity or p21 expression rescues developmental defects caused by BBX knockdown, including abnormal distribution and impaired morphology of newborn neurons in the developing cortex.

## Abstract (web summary)

BBX promotes mammalian cortical development by maintaining neural stem/progenitor cells and supporting newborn neuron migration. By restraining p53-mediated p21 expression, BBX prevents premature cell-cycle exit and senescence-associated changes, safeguarding normal brain development.

## Graphical abstract

**Figure:**
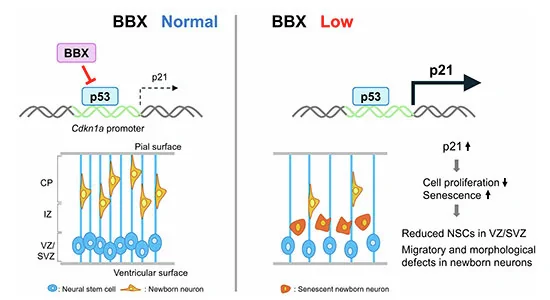

## Introduction

Brain development is a highly coordinated biological process that generates a functional brain with appropriate size and cellular diversity, driven by the tightly regulated self-renewal and differentiation of neural stem cells (NSCs) residing in the ventricular zone (VZ) (Rakic et al, 1974; Breunig et al, 2011; Kim et al, 2018). Once committed to a neuronal fate, these cells exit the VZ and migrate toward the brain’s outer surface, thereby establishing the laminar architecture of the neocortex (Kwan et al, 2012; Greig et al, 2013). Perturbations in the balance of NSC proliferation and differentiation result in a wide spectrum of developmental abnormalities, ranging from cognitive impairments to microcephaly (Rubenstein, 2011; Hu et al, 2014; Tang et al, 2016).

The regulation of NSC fate is governed by a complex interplay of extracellular cues and intracellular signaling pathways, many of which converge on changes in transcription factor expression levels or activity within the nucleus (Yoon and Gaiano, 2005; Abematsu et al, 2006; Nord et al, 2015). A previous study reported that direct reprogramming via forced expression of SOX2 alone in mouse and human fibroblasts can generate induced NSCs that closely resemble wild-type NSCs in terms of self-renewal ability and multipotency (Ring et al, 2012). Similarly, a separate study showed that overexpression of NeuroD1 alone can directly transdifferentiate reactive glial cells into functional neurons in mouse models of stab injury and Alzheimer’s disease (Guo et al, 2014). These findings underscore the pivotal roles of transcription factors in governing NSC proliferation and differentiation.

Since NSCs and neural progenitor cells reside predominantly in the germinal zones of the developing cortex, including the VZ and subventricular zone (SVZ), genes essential for NSC/progenitor function are expected to be enriched in these regions. Based on this reasoning, we surveyed the literature for candidate genes enriched in the germinal zones, predicted to function as transcription factors, and whose roles in brain development were largely unexplored. Among such genes, we identified bobby sox homolog (BBX).

BBX is a nuclear protein harboring a high mobility group box (HMG box) DNA-binding domain (Pöhler et al, 1998), suggesting its function as a transcription factor (Chen et al, 2014). Its transcriptional role was first demonstrated in fission yeast, where BBX was shown to promote the G1/S transition in an HMG box-dependent manner (Sánchez-Díaz et al, 2001). In mammals, BBX has been associated with bone and tooth development (Bassett et al, 2012; Choi et al, 2014), as well as with male fertility through its role in spermatid survival (Wang et al, 2016). Most relevant to brain development, BBX has been found in the embryonic hippocampus and neocortex (Dixon et al, 2013), and its transcription is repressed by NFIX, a factor that suppresses progenitor-specific pathways (Dixon et al, 2013; Heng et al, 2012). However, the precise function and regulatory mechanism of BBX in the developing mammalian brain remain to be defined.

In this study, we demonstrate that BBX is required for the maintenance of NSC properties in vitro, and that BBX knockdown in vivo disrupts multiple neurodevelopmental processes, including the morphology and migration of newborn neurons. Furthermore, we show that these defects are attributable to increased expression of p53-induced p21. Our findings provide detailed insights into the molecular mechanisms underlying BBX-mediated regulation of NSCs and its critical function during brain development.

## Results

### BBX maintains NSC properties in vitro

**Figure 1:**
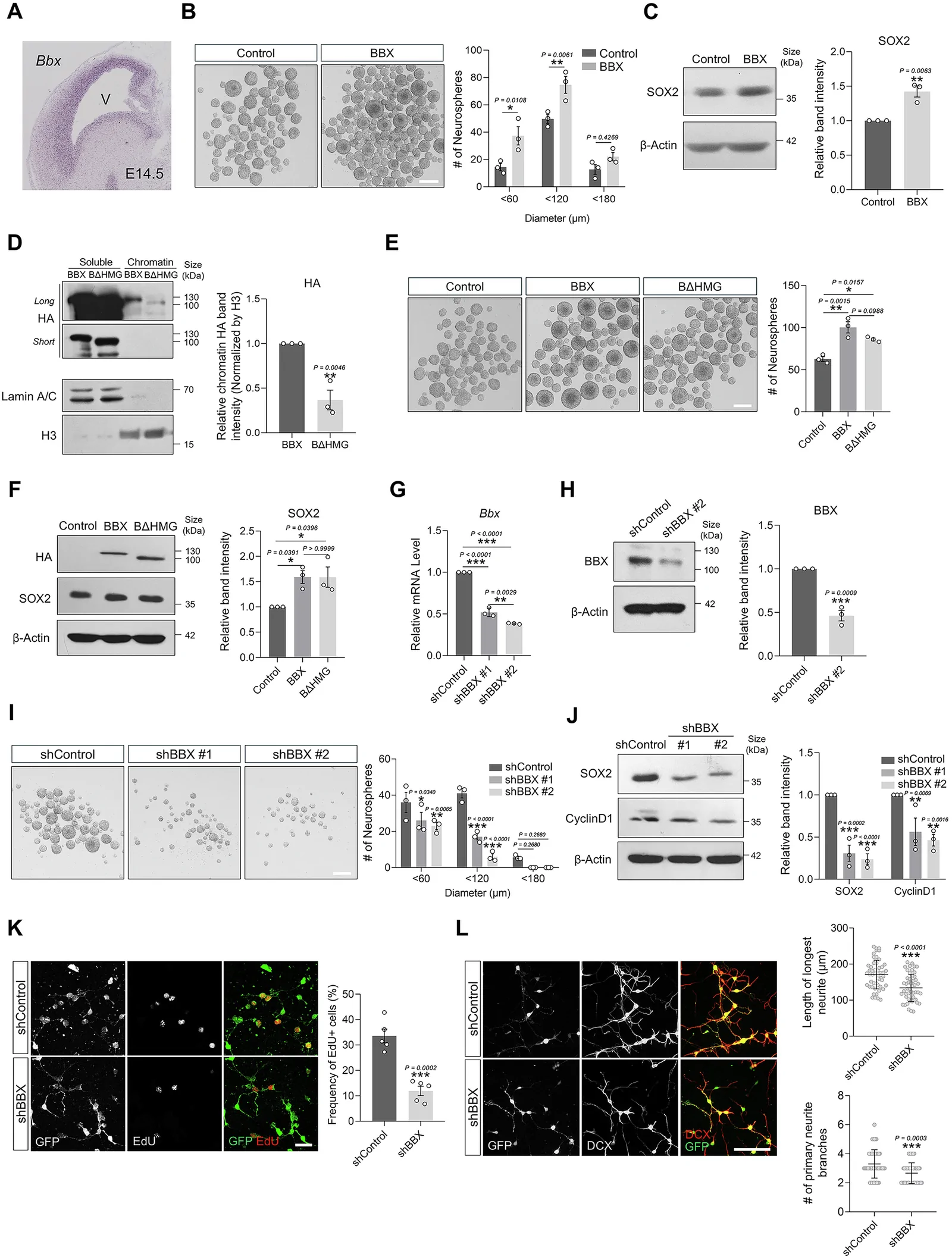
BBX is required for the maintenance of self-renewal-associated NSC properties in vitro. (**A**) In situ hybridization image of a sagittal section showing *Bbx* expression in the germinal zone of the developing mouse brain at E14.5. The image was obtained from the GenePaint database. V ventricle. (**B**) Neurosphere assay using NSCs transduced with lentiviral vectors expressing BBX. Scale bar, 200 μm. The graph shows neurosphere quantification (*n* = 3). (**C**) Western blot analysis of SOX2 expression in NSCs overexpressing BBX. The graph shows western blot quantification (*n* = 3). Unless otherwise stated, all western blot data were normalized to β-actin. (**D**) Chromatin association assay of HA-tagged wild-type BBX and BΔHMG in HEK293T cells. HEK293T cells were transfected with plasmids expressing HA-tagged BBX or BΔHMG and harvested 2 days after transfection. Nuclear extracts were separated into soluble and chromatin-associated fractions, followed by western blot analysis with an anti-HA antibody. Long and short exposures of the HA blot are shown. Lamin A/C and histone H3 were used to validate the soluble and chromatin-associated nuclear fractions, respectively. The graph shows quantification of HA signal intensity in the chromatin-associated fraction normalized to H3 (*n* = 3). (**E**) Neurosphere assay images of NSCs transduced with BBX or BΔHMG lentiviruses. Scale bar, 200 μm. The graph shows neurosphere quantification (*n* = 3). (**F**) Detection of SOX2 by western blot in NSCs transduced with HA-tagged BBX or BΔHMG lentiviruses. The graph shows western blot quantification (*n* = 3). (**G**) qPCR analysis of the knockdown efficiency of two shRNA vectors targeting *Bbx*, shBBX #1 and shBBX #2, in NSCs (*n* = 3). (**H**) Western blot analysis of endogenous BBX protein expression in NSCs transduced with shBBX #2. The graph shows quantification of relative BBX band intensity (*n* = 3). (**I**) Neurosphere assay using NSCs transduced with shBBX #1 or shBBX #2 lentivirus. Scale bar, 200 μm. The graph shows neurosphere quantification (*n* = 3). (**J**) Western blot analysis of SOX2 and CyclinD1 in shBBX-transduced NSC lysates. The graph shows western blot quantification (*n* = 3). Hereafter, “shBBX” refers to shBBX #2, unless otherwise specified. (**K**) EdU incorporation assay in shBBX-transduced NSCs. NSCs were plated on poly-L-ornithine- and laminin-coated plates. After 12 h, cells were pulsed with EdU for 1 h, fixed, and immunostained for GFP (green) and EdU incorporation (red). The graph shows the frequency of EdU⁺ cells among GFP⁺ cells (*n* = 5). Scale bar, 50 μm. (**L**) In vitro neuronal differentiation of NSCs following BBX knockdown. NSCs were transduced with shBBX and cultured for 3 days in differentiation medium lacking FGF. Cells were then fixed and immunostained for GFP (green) and DCX (red). Scale bar, 100 μm. The graphs present quantification of the longest neurite length and the number of primary neurite branches (*n* = 50 cells from three biological replicates). Data information: Data are presented as mean ± SEM from three independent biological replicates. Statistical significance was determined by the unpaired Student’s *t* test (**C**, **D**, **H**, **K**, **L**), one-way ANOVA (**E**–**G**), and two-way ANOVA (**B**, **I**, **J**). *P < 0.05, **P < 0.01, ***P < 0.001. Source data are available online for this figure.

**Figure EV1:**
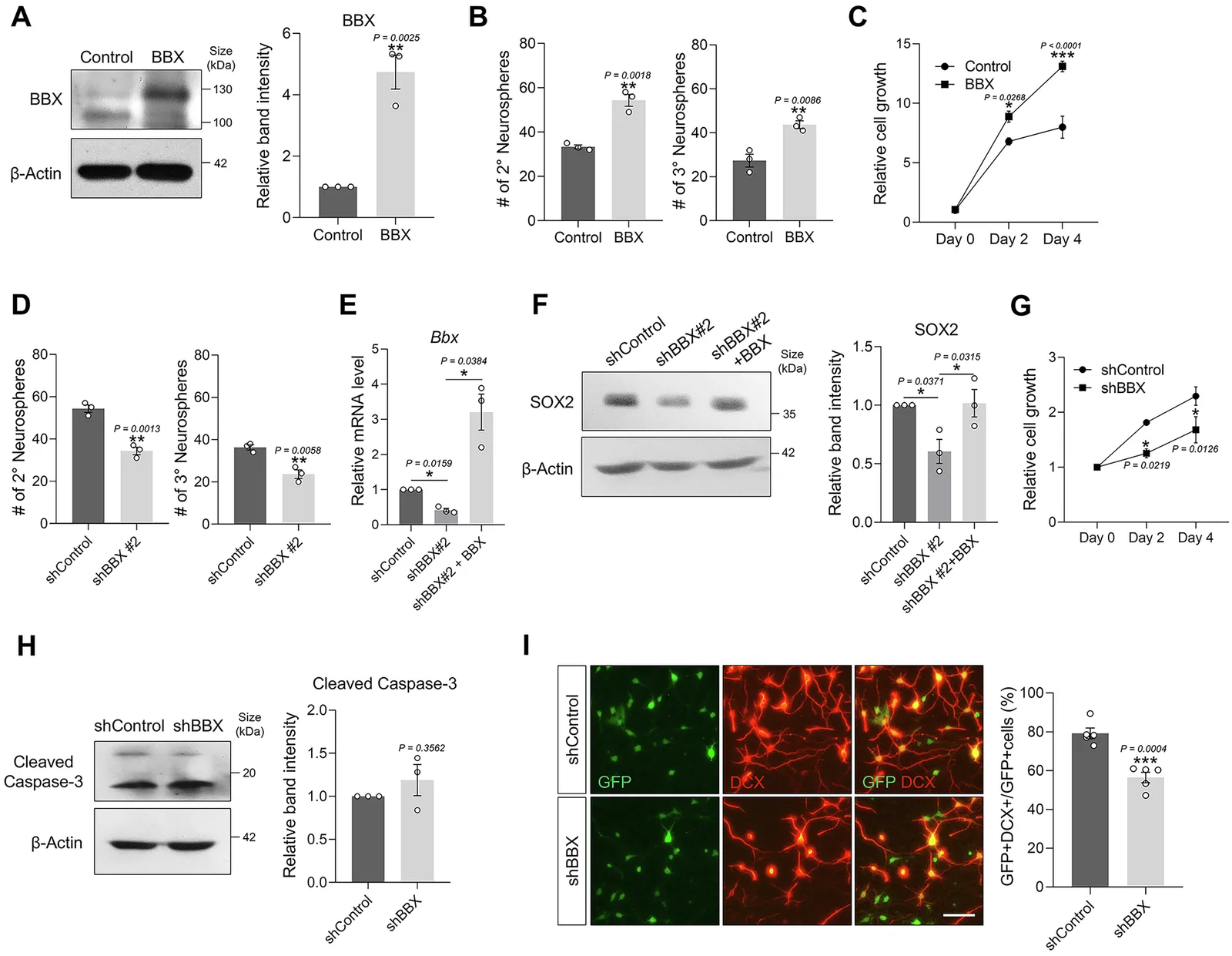
BBX regulates neurosphere self-renewal and SOX2 expression in NSCs in vitro. (**A**) Validation of BBX overexpression in NSCs following lentiviral transduction. NSCs were transduced with control or BBX-expressing lentivirus and harvested 2 days later for western blot analysis using an anti-BBX antibody. The graph shows quantification of relative BBX band intensity (*n* = 3). (**B**) Quantification of secondary and tertiary neurospheres formed by NSCs transduced with control or BBX-expressing lentiviral vectors. Primary neurospheres were dissociated and replated at 3000 cells per well to generate secondary neurospheres, and the same procedure was repeated to generate tertiary neurospheres (*n* = 3). (**C**) XTT assay showing the growth of NSCs transduced with lentiviral vectors expressing BBX. Relative cell growth was assessed on days 0, 2, and 4 after seeding (*n* = 3). (**D**) Secondary and tertiary neurosphere assays using NSCs transduced with control or shBBX #2 lentiviral vectors (*n* = 3). Assays were performed as described in (**B**). (**E**) Validation of BBX expression in rescue conditions. NSCs were transduced with shControl, shBBX #2, or shBBX #2 together with BBX-expressing lentivirus, and *Bbx* mRNA levels were measured by qPCR (*n* = 3). (**F**) Western blot analysis showing that BBX co-expression restores SOX2 protein levels reduced by shBBX #2 in NSCs. The graph shows quantification of relative SOX2 band intensity (*n* = 3). (**G**) XTT assay assessing the growth of NSCs transduced with shBBX lentivirus. Relative cell growth was measured on days 0, 2, and 4 after seeding (*n* = 3). (**H**) Western blot analysis of cleaved caspase-3 levels in NSCs transduced with shControl or shBBX lentivirus. Cells were harvested 3 days after transduction and subjected to immunoblotting with an anti-cleaved caspase-3 antibody (*n* = 3). (**I**) GFP (green) and DCX (red) immunostaining in NSCs transduced with shControl or shBBX lentivirus and cultured for 2 days under differentiation conditions. The graph shows the percentage of GFP⁺DCX⁺ cells among GFP⁺ cells. The graph shows immunostaining quantification (*n* = 5). Scale bar, 100 μm. Data information: Data are presented as mean ± SEM from at least three independent biological replicates. Statistical significance was determined by the unpaired Student’s *t* test (**A**, **B**, **D**, **H**, **I**), one-way ANOVA (**E**, **F**), and two-way ANOVA (**C**, **G**). *P < 0.05, **P < 0.01, ***P < 0.001. Source data are available online for this figure.

BBX was previously reported to be expressed within the VZ of the developing mouse neocortex (Dixon et al, 2013). To further verify this expression pattern, we examined an in situ hybridization image obtained from the GenePaint database (http://www.genepaint.org). Consistent with the previous report, *Bbx* expression was enriched in the germinal zones of the developing cortex, including the VZ and SVZ (Fig. 1A). To investigate the functional role of BBX in NSCs, we started with a neurosphere assay (Reynolds and Weiss, 1992). Throughout this study, NSCs refer to mouse embryonic NSCs derived from developing brain tissues. All gene delivery vectors used in this study, whether designed to express a coding sequence or a short hairpin RNA (shRNA), contain a GFP reporter cassette, allowing transduced or electroporated cells to be identified by GFP expression. Negative controls received the corresponding backbone vector and therefore expressed GFP alone. Lentiviral-mediated BBX overexpression in NSCs derived from embryonic day 14.5 (E14.5) brain tissues increased BBX protein expression approximately fivefold relative to control levels and increased neurosphere number, including larger-diameter neurospheres (Figs. EV1A and  1B). BBX overexpression also increased secondary and tertiary neurosphere formation upon serial passaging (Fig. EV1B). Consistent with these observations, BBX overexpression increased the levels of SOX2 protein, an NSC marker (Zappone et al, 2000) (Fig. 1C). In addition, XTT assays, which measure metabolically active cell number, showed that BBX overexpression increased NSC growth over time (Fig. EV1C). Together, these results suggest that BBX promotes self-renewal-associated neurosphere-forming capacity and helps maintain stem/progenitor-associated characteristics in NSCs.

BBX encodes a protein containing an HMG box domain at its N-terminus and has been proposed to function as a DNA-binding transcription factor (Chen et al, 2014). However, HMG box-containing proteins can interact with DNA through diverse modes, including recognition of distorted DNA structures, association with promoter regions without clearly defined sequence specificity, or sequence-specific DNA binding (Chen et al, 2014). Although the mode by which the BBX HMG box domain engages DNA remains unclear, these possibilities commonly imply chromatin association. We therefore first asked whether the HMG box domain contributes to the chromatin association of BBX. To this end, we generated an HMG box deletion mutant, BΔHMG, in which amino acids 80-148 were removed from full-length BBX, an 888-amino-acid protein, by site-directed mutagenesis. Cellular fractionation confirmed that BΔHMG was substantially reduced in the chromatin fraction compared with wild-type BBX, indicating impaired chromatin association (Fig. 1D). Unexpectedly, however, BΔHMG enhanced neurosphere formation (Fig. 1E) and increased SOX2 protein expression (Fig. 1F) to levels comparable to those induced by wild-type BBX, suggesting that the HMG box domain is not required for BBX-mediated enhancement of NSC properties.

We also performed a loss-of-function study using short hairpin RNA (shRNA) to assess the functional significance of the *Bbx* gene. Both shRNA constructs targeting *Bbx*, shBBX #1 and shBBX #2, effectively reduced *Bbx* mRNA levels to less than 50% of the control (Fig. 1G), with shBBX #2 showing stronger knockdown efficiency. Western blot analysis further confirmed that shBBX #2 reduced endogenous BBX protein levels by approximately 50% (Fig. 1H). Suppression of BBX expression led to a substantial reduction in neurosphere formation and the number of larger neurospheres (Fig. 1I), along with decreased SOX2 protein levels (Fig. 1J). Consistently, secondary and tertiary neurosphere assays using shBBX #2 showed reduced neurosphere formation upon serial passaging (Fig. EV1D). Cyclin D1, a marker of cell cycle progression (Motokura et al, 1991; Xiong et al, 1991), was also reduced (Fig. 1J). Throughout these experiments, shBBX #2 was more effective than shBBX #1, and the correlation between more effective BBX silencing and stronger impairment of neurosphere-forming capacity and SOX2/Cyclin D1 expression argued against a major off-target contribution. Supporting this interpretation, BBX re-expression restored Bbx mRNA levels to higher than control levels and rescued the reduction in SOX2 caused by shBBX (Fig. EV1E,F). Therefore, in subsequent experiments, shBBX #2 was primarily used alone, and unless otherwise specified, “shBBX” refers to shBBX #2.

In line with the reduced neurosphere formation and Cyclin D1 expression, XTT assays showed that shBBX decreased NSC growth over time (Fig. EV1G), and 5-ethynyl-2′-deoxyuridine (EdU) incorporation assays revealed reduced DNA synthesis under proliferative conditions (Fig. 1K). In contrast, cleaved caspase-3 levels were not significantly altered by BBX knockdown (Fig. EV1H), suggesting that the reduced NSC growth was not primarily attributable to increased apoptotic cell death. Together, these knockdown results are consistent with the overexpression findings and support the conclusion that BBX is required for maintaining stem/progenitor-associated characteristics in NSCs.

We further investigated the effects of BBX depletion under differentiation conditions. BBX knockdown reduced the generation of immature neurons, identified by immunostaining for DCX, a marker of immature and migrating neurons (Francis et al, 1999; Gleeson et al, 1999) (Fig. EV1I). BBX knockdown also disrupted neurite outgrowth, as assessed by the length of the longest neurite and the number of primary neurite branches (Fig. 1L). Collectively, these findings suggest that BBX is required for maintaining self-renewal-associated stem/progenitor characteristics in embryonic NSCs under proliferative conditions, while also supporting neuronal differentiation and subsequent neurite outgrowth under differentiation conditions.

### BBX regulates NSC characteristics and disrupts the migration of newborn neurons in vivo

**Figure 2:**
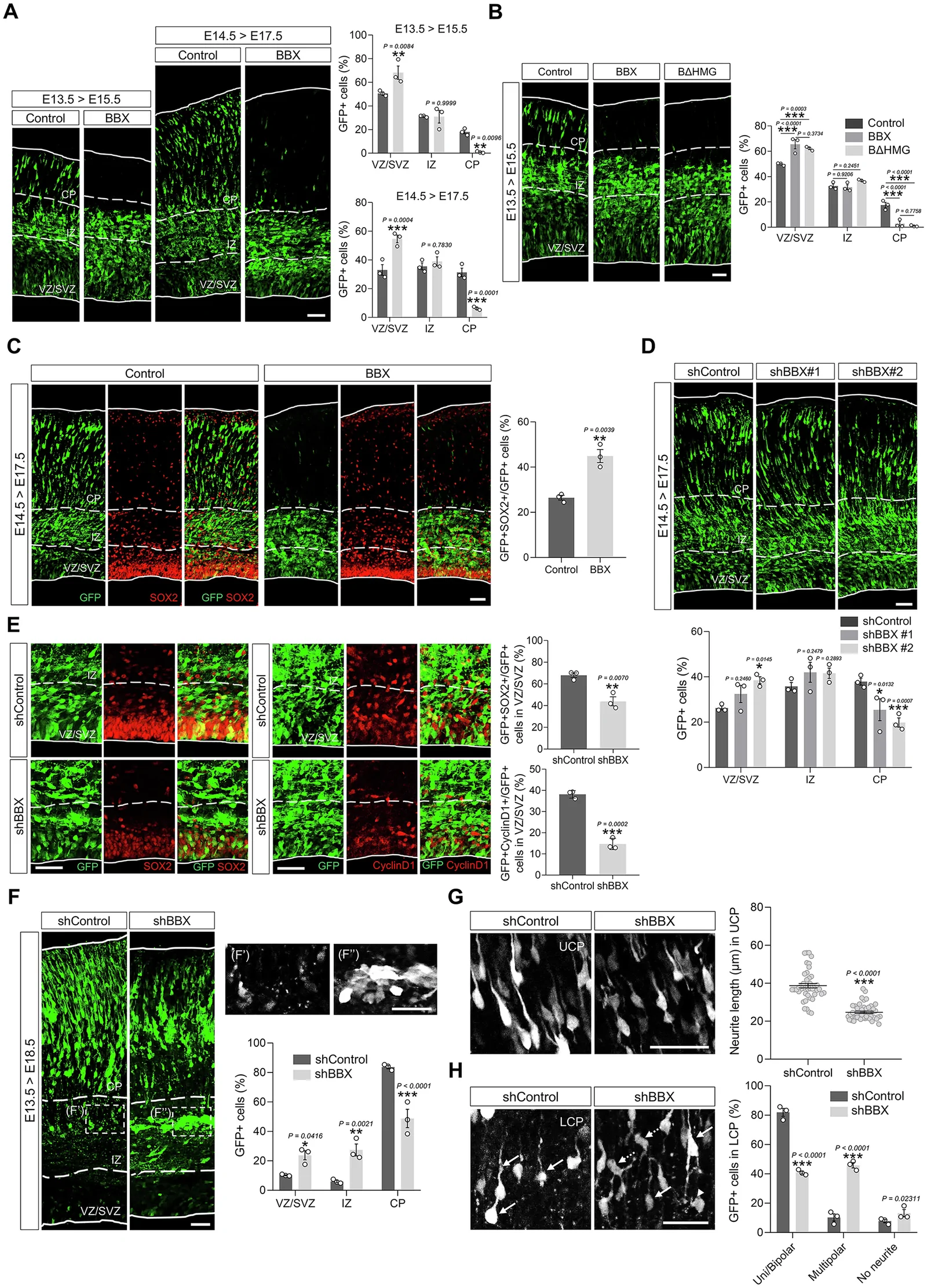
BBX knockdown in vivo reduces Sox2^+^ cells and impairs cortical neuronal migration. (**A**) GFP immunostaining of E15.5 and E17.5 mouse cortices electroporated with a BBX expression vector at E13.5 and E14.5, respectively. Scale bar, 50 μm. The graphs show immunostaining quantification (*n* = 3). (**B**) Immunostaining for GFP in E15.5 brains electroporated at E13.5 with BBX or BΔHMG expression constructs. Scale bar, 25 μm. The graph shows immunostaining quantification (*n* = 3). (**C**) Co-immunostaining for GFP (green) and SOX2 (red) in E17.5 brain sections electroporated with BBX at E14.5. Scale bar, 50 μm. The graph shows immunostaining quantification (*n* = 3). (**D**) GFP immunostaining of E17.5 mouse cortices electroporated at E14.5 with shBBX vectors. Scale bar, 50 μm. The lower graph shows quantification of GFP⁺ cell distribution across cortical layers (*n* = 3). (**E**) Co-immunostaining for GFP (green) and SOX2 (red), or CyclinD1 (red), in E17.5 cortices electroporated at E14.5 with shBBX. Scale bar, 50 μm. The graphs show immunostaining quantification (*n* = 3). (**F**) GFP immunostaining of E18.5 cortices electroporated with shBBX at E13.5. Boxed regions are shown at higher magnification in F′ and F′′. Scale bars, 50 μm. The graph shows immunostaining quantification (*n* = 3). (**G**) GFP immunostaining of the upper cortical plate (UCP) in E18.5 brains electroporated with shBBX at E13.5 in grayscale images. Scale bars, 50 μm. The graph shows immunostaining quantification (*n* = 45 cells from 3 biological replicates). (**H**) GFP immunostaining of the lower cortical plate (LCP) in E18.5 brains electroporated at E13.5 with shBBX. Scale bar, 50 μm. The graph shows the percentage of neurons exhibiting unipolar/bipolar (arrow), multipolar (dotted arrow), or no neurite (arrowhead) morphology (*n* = 3). (**A**–**F**) Solid white lines mark the apical and basal borders of the cortical tissue sections, whereas dashed white lines indicate the boundaries between the ventricular/subventricular zone (VZ/SVZ), intermediate zone (IZ), and cortical plate (CP). Data information: Data are presented as mean ± SEM from three independent biological replicates. Statistical significance was determined by the unpaired Student’s *t* test (**C**, **E**, **G**) and two-way ANOVA (**A**, **B**, **D**, **F**, **H**). *P < 0.05, **P < 0.01, ***P < 0.001. Source data are available online for this figure.

**Figure EV2:**
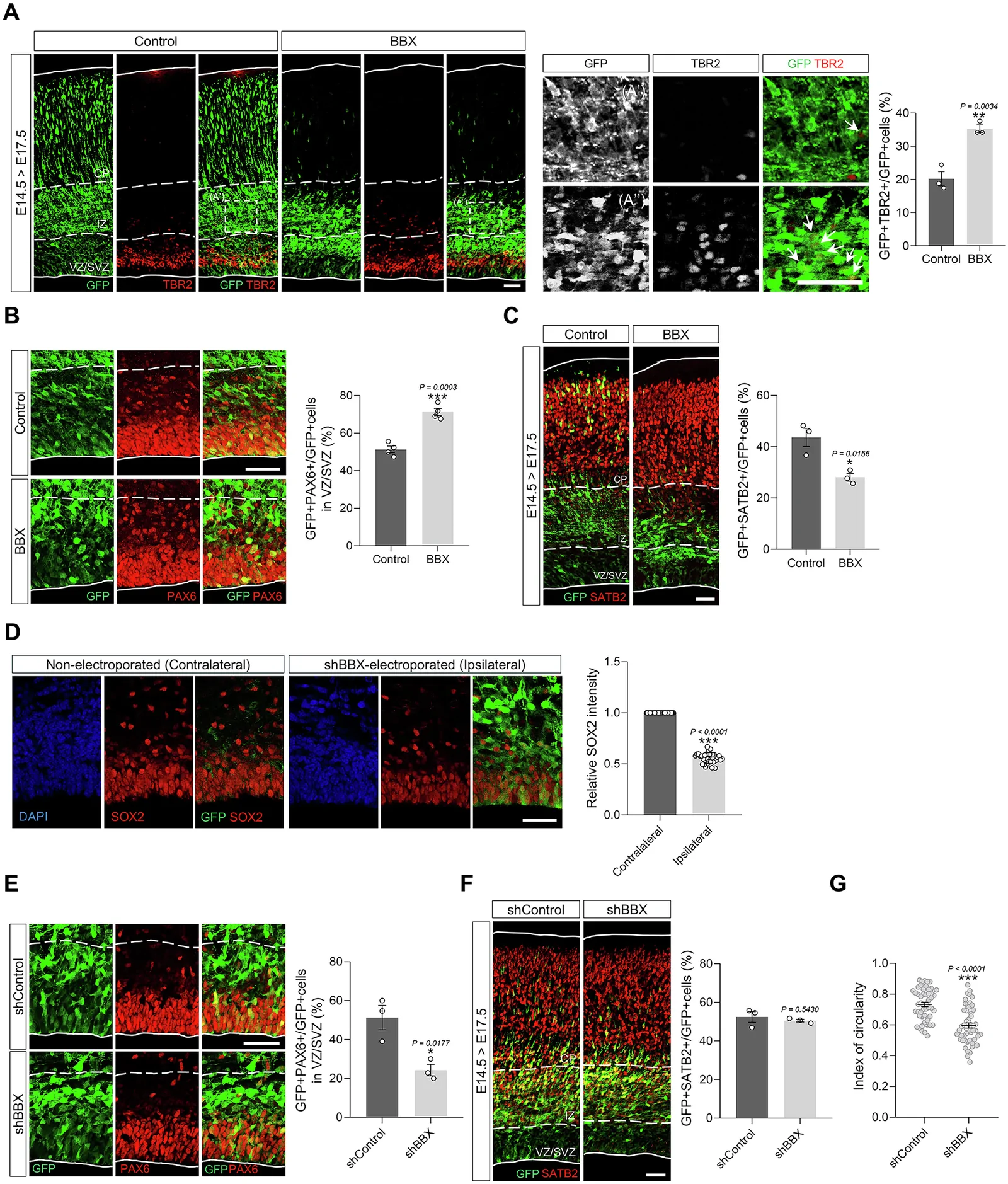
BBX modulates cortical progenitor populations and neuronal differentiation marker expression in vivo. For (**A**–**G**), embryonic cortices were electroporated with the indicated plasmids at E14.5 and analyzed at E17.5. (**A**) Immunostaining for GFP and TBR2 in brain sections electroporated with a control or BBX expression vector. Magnified views of the boxed areas are shown in (**A′**, **A′′**). Arrows indicate GFP⁺TBR2⁺ cells. The graph shows the percentage of GFP⁺TBR2⁺ cells among GFP⁺ cells (*n* = 3). Scale bars, 50 μm. (**B**, **C**) Immunostaining for PAX6 (**B**) and SATB2 (**C**) in embryonic cortices electroporated with control or BBX expression plasmids. Graphs show the frequency of PAX6⁺ or SATB2⁺ cells among GFP⁺ cells (*n* = 4 for (**B**) and *n* = 3 for (**C**)). Scale bars, 50 μm. (**D**) Comparison of SOX2 fluorescence intensity between shBBX-electroporated and non-electroporated VZ regions within the same brain section. Sections were immunostained for GFP (green) and SOX2 (red). The graph shows relative SOX2 fluorescence intensity in the ipsilateral shBBX-electroporated VZ compared with the contralateral non-electroporated VZ (*n* = 30 cells from three biological replicates). Scale bar, 50 μm. (**E**, **F**) Immunostaining for PAX6 (**E**) and SATB2 (**F**) in embryonic cortices electroporated with shControl or shBBX. GFP marks electroporated cells. The graphs show quantification of PAX6⁺ or SATB2⁺ cells among GFP⁺ cells (*n* = 3). Scale bars, 50 μm. (**G**) Analysis of GFP⁺ cell body morphology shown in Fig. 2H. Circularity was calculated using the formula 4π × area/perimeter². A value of 1 represents a perfect circle, whereas lower values indicate increasingly elongated or irregular morphologies (*n* = 50 cells from three biological replicates). In (**A**–**C**, **E**, **F**), solid white lines mark the apical and basal borders of the cortical tissue sections, whereas dashed white lines indicate the boundaries between the VZ/SVZ, IZ, and CP. Data information: Data are presented as mean ± SEM from three biological replicates. Statistical significance was determined by the unpaired Student’s *t* test. *P < 0.05, **P < 0.01, ***P < 0.001. Source data are available online for this figure.

We next investigated the in vivo function of BBX by performing in utero electroporation (Saito and Nakatsuji, 2001). BBX was introduced into the developing brain at E13.5, and the distribution of electroporated cells was analyzed at E15.5 (hereafter referred to as E13.5 > E15.5). In this setting, BBX overexpression caused a pronounced shift in the cortical laminar distribution of GFP-positive cells, with increased accumulation in the VZ/SVZ and reduced localization in the cortical plate (CP) compared with controls (Fig. 2A). A similar shift was observed in the E14.5 > E17.5 setting (Fig. 2A). Consistent with the in vitro finding that BΔHMG retained activity comparable to wild-type BBX, overexpression of BΔHMG produced a similar effect on cortical cell distribution in vivo (Fig. 2B). Immunostaining for progenitor markers in the VZ/SVZ further revealed that BBX overexpression increased the proportions of SOX2⁺ cells (Fig. 2C) and TBR2⁺ intermediate progenitor cells (Sessa et al, 2008; Hevner, 2019) (Fig. EV2A). When the analysis was restricted to GFP⁺ cells located in the VZ/SVZ, immunostaining for PAX6, another progenitor marker (Götz et al, 1998), showed that BBX overexpression also increased the proportion of PAX6⁺ cells (Fig. EV2B). In contrast, immunostaining for SATB2, a marker of upper-layer cortical neurons (Britanova et al, 2008), showed that BBX overexpression substantially reduced the proportion of SATB2⁺ cells among GFP⁺ cells (Fig. EV2C). These findings suggest that the increased VZ/SVZ distribution caused by BBX overexpression is associated with expansion or retention of progenitor-marker-positive populations and reduced progression toward a SATB2⁺ upper-layer neuronal fate at this developmental stage.

Next, we examined the in vivo effects of BBX knockdown. In the E14.5 > E17.5 setting, shBBX-transduced cells showed increased accumulation in the VZ and reduced localization in the CP relative to controls (Fig. 2D). Consistent with the stronger knockdown efficiency of shBBX #2 in vitro, shBBX #2 produced more pronounced distributional changes than shBBX #1. At first glance, the shift in cellular distribution resembled that observed upon BBX overexpression. However, immunostaining revealed a clear distinction in shBBX samples. The proportion of SOX2⁺ cells in the VZ/SVZ was reduced, and SOX2 fluorescence intensity within the remaining SOX2⁺ cells was substantially diminished (Fig. 2E). To minimize the possibility that the reduced SOX2 signal reflected technical variability in immunostaining between tissue sections, we compared SOX2 fluorescence intensity within the same brain section. Specifically, SOX2 intensity was measured in the shBBX-electroporated VZ and the contralateral non-electroporated VZ. This same-section comparison confirmed that SOX2 fluorescence intensity was substantially reduced in shBBX-electroporated cells relative to contralateral control cells (Fig. EV2D). These findings indicate that BBX knockdown reduces both the proportion of SOX2⁺ cells in the VZ/SVZ and SOX2 signal intensity within the remaining SOX2⁺ population in vivo, consistent with the reduction in SOX2 expression observed by western blot analysis in vitro. In addition, BBX knockdown reduced the proportions of PAX6⁺ and Cyclin D1⁺ cells in the VZ/SVZ to less than half of control levels (Figs. EV2E and  2E). In contrast, the proportion of SATB2⁺ cells was not significantly altered in shBBX samples (Fig. EV2F). These findings indicate that, although BBX overexpression and BBX knockdown may appear to cause similar changes in cell distribution, they are associated with distinct marker-defined cellular states. BBX overexpression increased progenitor-marker-positive populations, whereas BBX knockdown reduced them, supporting a role for BBX in maintaining neural progenitor characteristics.

In the E13.5 > E18.5 setting, these abnormalities became more evident. Whereas control cells mainly localized to the CP, a substantial number of shBBX-transduced cells remained in the intermediate zone (IZ), forming clusters (Fig. 2F–F″). Abnormalities were also observed among shBBX-transduced cells in the CP. In the upper CP, the leading processes of shBBX-transduced cells failed to reach the pial surface (Fig. 2F), and their neurite lengths were reduced (Fig. 2G). In the lower CP, shBBX-transduced cells more frequently exhibited multipolar morphology, in contrast to the predominantly uni/bipolar morphology of control cells (Fig. 2H). Moreover, BBX-knockdown cells displayed irregular morphologies, in contrast to the typically round cell bodies observed in control cells. To quantify this morphological change, we measured the circularity index, a commonly used metric for assessing cell shape (Ankam et al, 2018; Boi et al, 2024). BBX knockdown significantly reduced the circularity index of GFP⁺ cells, indicating more irregular cell morphology in BBX-depleted cells (Fig. EV2G). Collectively, these findings suggest that BBX contributes to the maintenance of neural progenitor populations in vivo and supports proper neuronal morphology and migration during cortical development.

### Combined transcriptomic and chromatin profiling identifies p21 as a candidate target of BBX

**Figure 3:**
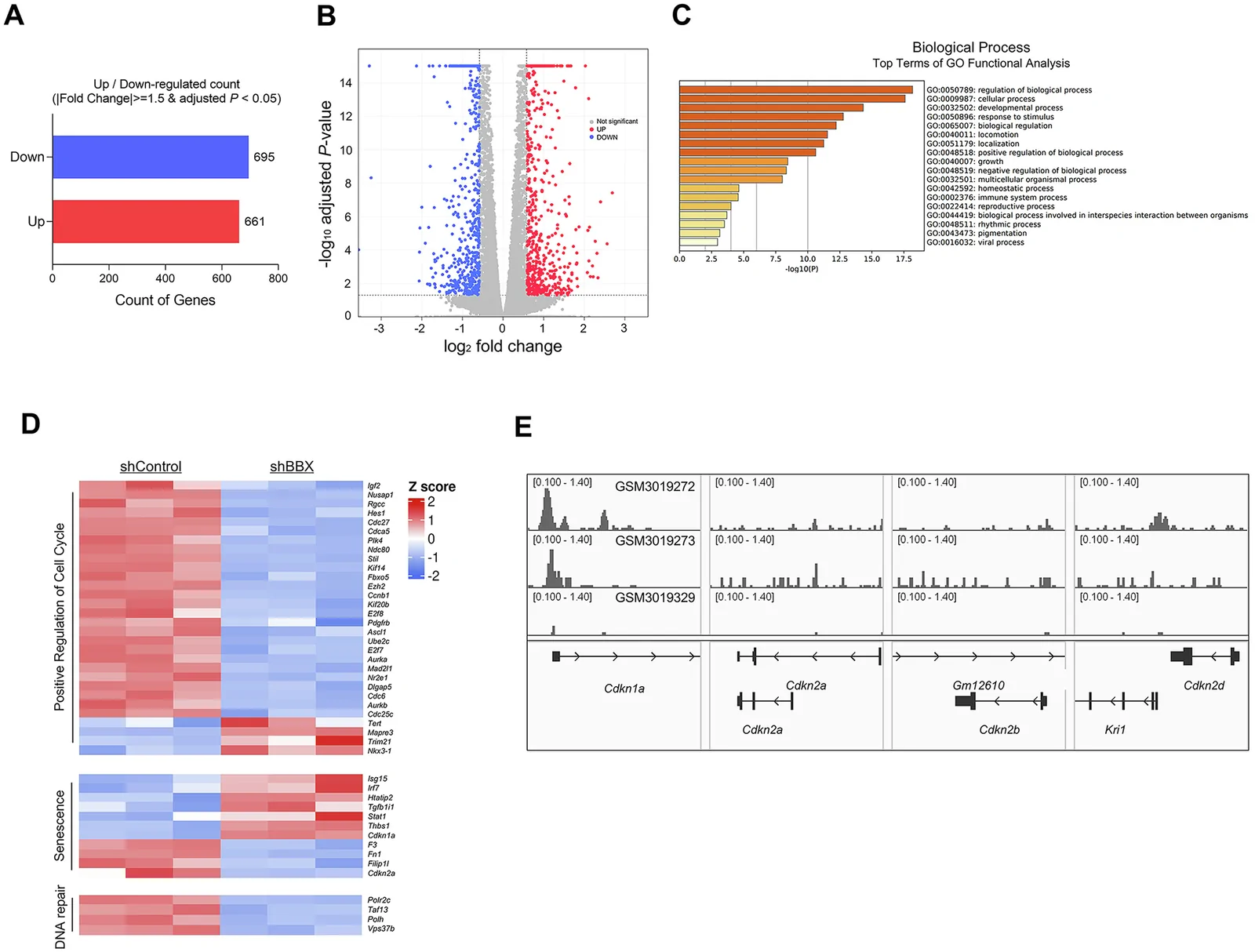
Integrated RNA-seq and ChIP-seq analyses identified p21 as a putative direct transcriptional target of BBX. (**A**, **B**) The total number of differentially expressed genes (DEGs) identified by RNA-seq in shBBX-transduced NSCs. DEGs were defined using DESeq2 with the Wald test as genes with an absolute fold change ≥1.5 and Benjamini–Hochberg-adjusted *P* < 0.05. Data are shown as a bar graph (**A**) and a volcano plot (**B**) (*n* = 3). (**C**) Gene Ontology (GO) Biological Process enrichment analysis of DEGs identified in shBBX-transduced NSCs. Enriched GO terms were identified using Metascape based on the cumulative hypergeometric test. Bars indicate −log10(*P*), and the corresponding GO identifiers and term names are listed on the right. (**D**) Heatmap of selected DEGs associated with biological processes relevant to the BBX knockdown phenotype, including cell-cycle regulation, senescence, and DNA repair. Genes were selected from these functional categories among DEGs satisfying the criteria described in (**A**, **B**). Expression values are presented as row-scaled Z-scores. (**E**) Integrative Genomics Viewer (IGV) visualization of a publicly available BBX ChIP-seq dataset from trophoblast stem cells (GSE110950; Data ref: Lee et al, 2019), showing BBX enrichment at selected *Cdkn* gene loci. GEO samples GSM3019272 and GSM3019273 were generated under proliferative conditions, whereas GSM3019329 was obtained under differentiation conditions. Gene model annotations are shown below the ChIP-seq tracks; boxes indicate exons, connecting lines indicate intronic regions, and arrows indicate transcriptional orientation.

To elucidate the molecular mechanism by which BBX regulates the NSCs, we performed RNA sequencing (RNA-seq) in E13.5 NSCs transduced with shBBX lentivirus. This analysis identified 1,356 differentially expressed genes (DEGs) using an absolute fold change cutoff of ≥1.5 and a Benjamini–Hochberg-adjusted *P* value < 0.05, including 661 upregulated and 695 downregulated genes in BBX-depleted NSCs (Fig. 3A,B).

Gene Ontology (GO) Biological Process analysis of DEGs identified in shBBX-transduced NSCs revealed enrichment of terms related to biological regulation, cellular and developmental processes, responses to stimulus, and growth- or movement-associated processes (Fig. 3C). Given that BBX knockdown reduces cell growth in the in vitro XTT assay (Fig. EV1G), decreases Cyclin D1 protein levels in vitro (Fig. 1J), and reduces the number of Cyclin D1⁺ cells in vivo (Fig. 2E), we first examined DEGs associated with cell-cycle regulation and proliferation using gene sets extracted from the Molecular Signatures Database (MSigDB). In line with these growth- and cell-cycle-related phenotypes, genes associated with positive regulation of the cell cycle were broadly downregulated upon BBX knockdown (Fig. 3D). In parallel, because BBX-depleted neurospheres were not only reduced in size but also displayed morphologically compromised features suggestive of cellular stress (Fig. 1I), despite no detectable increase in cleaved caspase-3 levels (Fig. EV1H), we considered the possibility that BBX knockdown induces a non-apoptotic stress state, such as cellular senescence. Because senescence is closely linked to DNA damage signaling (Gorbunova et al, 2007), we also examined genes associated with DNA repair processes. Consistent with this possibility, multiple genes associated with senescence and DNA damage/repair responses were differentially expressed upon BBX knockdown (Fig. 3D). Of these, cyclin-dependent kinase inhibitor 1 A (*Cdkn1a*, p21) was particularly notable because p21 is a well-established cell-cycle regulator (el-Deiry et al, 1993; Harper et al, 1995) and also serves as an important mediator of cellular senescence (Brown et al, 1997; Vogt et al, 1998; Fang et al, 1999). Among the cell-cycle inhibitory regulators examined, *Cdkn1a* was the only gene that showed robust upregulation upon BBX knockdown. Although *Cdkn2a* was also identified as a DEG, it was downregulated rather than induced upon BBX knockdown.

To explore whether BBX-regulated genes include candidate loci directly associated with BBX, we examined a publicly available BBX ChIP-seq dataset generated from trophoblast stem cells (GSE110950; Data ref: Lee et al, 2019). Because the proliferative-condition dataset included only two samples (GSM3019272 and GSM3019273), and one of them showed relatively poor overall signal quality, we limited our analysis to candidate locus-focused inspection rather than systematic genome-wide target identification. This analysis revealed BBX occupancy at the *Cdkn1a* (p21) promoter, corresponding to a gene upregulated upon BBX knockdown in our RNA-seq analysis (Fig. 3E). Notably, this occupancy was detected in both proliferative-condition samples, GSM3019272 and GSM3019273, despite the relatively poor overall signal quality of GSM3019273, but not in the differentiated trophoblast sample GSM3019329. This observation suggests that BBX may preferentially associate with the *Cdkn1a* (p21) promoter under proliferative conditions. Consistent with our RNA-seq results, other cell cycle inhibitors such as *Cdkn2a* showed no evident ChIP-seq signal at their promoter regions (Fig. 3E). Collectively, our integrated RNA-seq and public ChIP-seq analyses identified p21 as a candidate direct BBX-associated target.

### BBX reduces p21 expression by interfering with p53 recruitment to the p21 promoter

**Figure 4:**
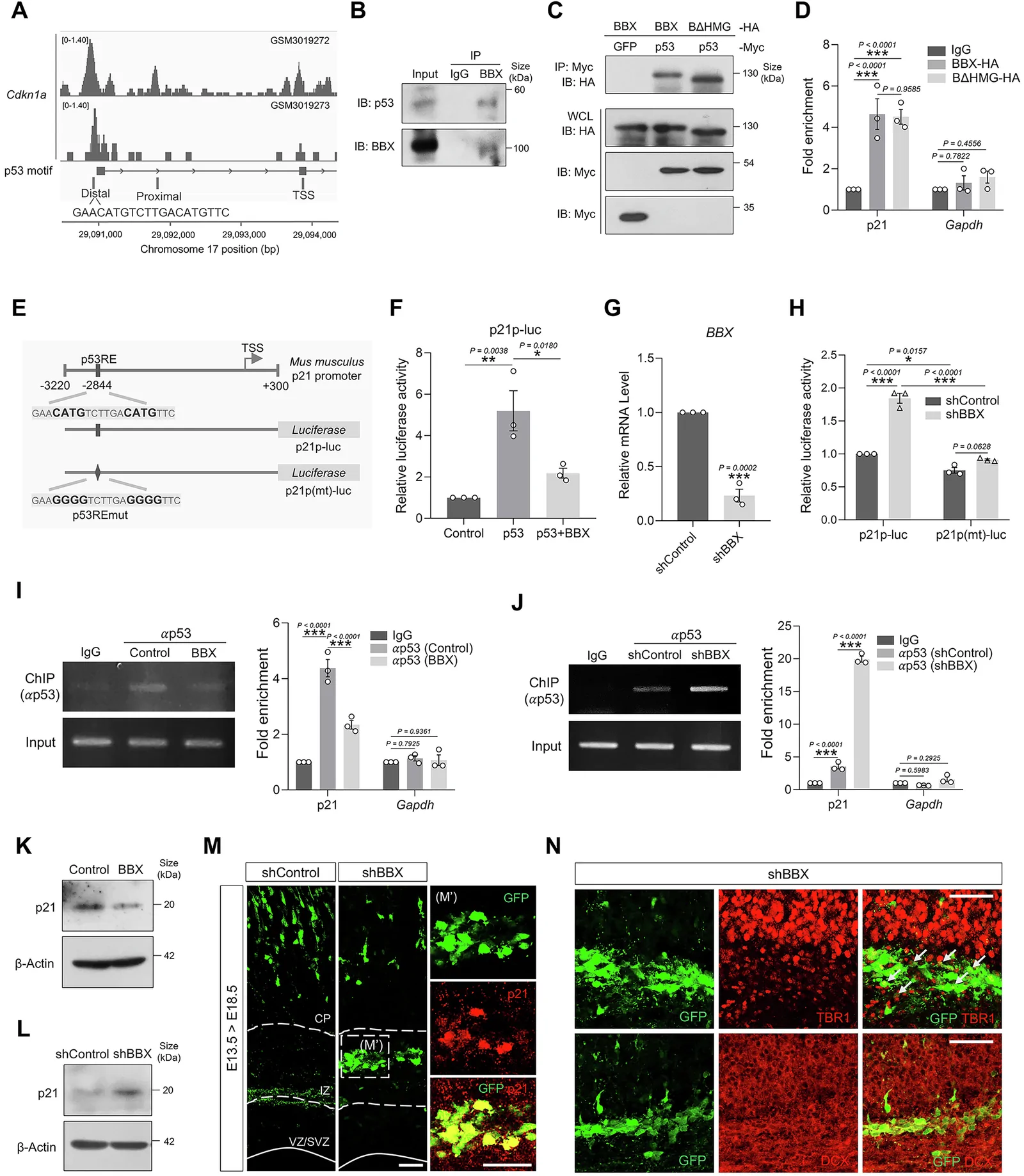
BBX regulates p21 gene expression by impairing the DNA-binding activity of p53. (**A**) Schematic showing the p53 binding site at the *Cdkn1a* promoter region, which overlaps with the strongest BBX binding peak under proliferative conditions, as revealed by ChIP-seq analysis (GSE110950). TSS, transcription start site. (**B**) CoIP analysis of endogenous BBX and p53 in E13.5 mouse cortex. Cortical lysates were immunoprecipitated with an anti-BBX antibody and immunoblotted with an anti-p53 antibody. (**C**) CoIP of Myc-tagged p53 with HA-tagged BBX or BΔHMG in HEK293T cells. (**D**) ChIP-qPCR analysis showing comparable enrichment of HA-tagged wild-type BBX and BΔHMG at the p21 promoter in NSCs. NSCs expressing HA-tagged BBX or BΔHMG were subjected to ChIP using an anti-HA antibody, followed by qPCR analysis of the p21 promoter region. The *Gapdh* locus was used as a negative control. The graph shows fold enrichment relative to IgG control (*n* = 3). (**E**) Schematic representation of the luciferase constructs used in this study. The p21p-luc vector contains the p21 promoter sequence spanning from −3220 to +300 bp relative to the transcription start site (TSS), which includes the p53 responsive element (p53RE). In the p21p (mt)-luc construct, CATG sequences within the p53RE were substituted with GGGG. (**F**) Luciferase reporter assays using the p21p-luc construct in U87MG cells transfected with p53 and/or BBX expression plasmids (*n* = 3). (**G**) qPCR analysis of *BBX* mRNA levels showing the knockdown efficiency of a human BBX-targeting shRNA vector in U87MG cells (*n* = 3). (**H**) Relative luciferase activity of the p21p-luc and p21p (mt)-luc reporters in U87MG cells expressing shControl or shBBX (*n* = 3). (**I**) ChIP-PCR analysis of the *Cdkn1a* (p21) promoter region containing a p53-binding site in BBX-overexpressing NSCs. ChIP was performed using an anti-p53 antibody. The graph quantifies p53 enrichment at the *Cdkn1a* (p21) promoter (*n* = 3). (**J**) ChIP-PCR analysis of the *Cdkn1a* (p21) promoter region in shBBX-transduced NSCs under the same experimental conditions as in (**I**). The graph quantifies p53 enrichment at the *Cdkn1a* (p21) promoter (*n* = 3). (**K**) Western blot analysis of p21 protein levels in BBX-transduced NSCs. (**L**) Detection of p21 by western blot in BBX-depleted NSCs. (**M**) Co-immunostaining of E18.5 brain sections electroporated at E13.5 with shBBX vectors using anti-GFP (green) and anti-p21 (red) antibodies. Solid white lines mark the apical border of the cortical tissue sections, whereas dashed white lines indicate the boundaries between the VZ/SVZ, IZ, and CP. The boxed area (**M’**) is shown at higher magnification in the right column, highlighting GFP and p21 double-positive cells. Scale bars, 50 μm. (**N**) Co-immunostaining of cell clusters in the IZ of shBBX samples using anti-GFP (green) together with either anti-TBR1 (red, upper panels) or anti-DCX (red, lower panels) antibodies. Arrows in the upper panels indicate GFP⁺TBR1⁺ double-labeled cells. Scale bars, 50 μm. Data information: Data are presented as mean ± SEM from three independent biological replicates. Statistical significance was determined by the unpaired Student’s *t* test (**G**), one-way ANOVA (**F**) and two-way ANOVA (**D**, **H**–**J**). *P < 0.05, **P < 0.01 ***P < 0.001. Source data are available online for this figure.

We next sought to determine how BBX regulates p21 transcription. Detailed analysis of the proliferative-condition BBX ChIP-seq data revealed that the strongest BBX-binding peak within the *Cdkn1a* (p21) promoter overlapped with a previously defined p53-binding motif, suggesting a potential link between BBX and p53 at this locus (Fig. 4A). Among the two previously defined p53 response elements in the *Cdkn1a* (p21) promoter, the distal and proximal elements (Macleod et al, 1995), the BBX ChIP-seq signal was more strongly associated with the distal element. These observations raised the possibility that BBX may be recruited to the *Cdkn1a* (p21) promoter through its association with p53. To test this possibility, we first performed endogenous co-immunoprecipitation (coIP) in E13.5 cortical lysates using an anti-BBX antibody and found that endogenous BBX was associated with p53 (Fig. 4B).

Since BΔHMG mirrored wild-type BBX activity across our experimental settings, we hypothesized that BBX may regulate p21 expression through interaction with p53, rather than through HMG box-dependent chromatin association. Consistent with this idea, coIP experiments showed that BΔHMG retained p53-binding ability comparable to that of wild-type BBX (Fig. 4C), indicating that the BBX–p53 interaction does not require the HMG box domain. We further performed ChIP-qPCR in NSCs expressing HA-tagged wild-type BBX or BΔHMG using an anti-HA antibody. Consistent with the ChIP-seq analysis, HA-tagged wild-type BBX was enriched at the *Cdkn1a* (p21) promoter, but not at the *Gapdh* control locus (Fig. 4D). Notably, BΔHMG was also enriched at the *Cdkn1a* (p21) promoter at a level comparable to that of wild-type BBX (Fig. 4D). These results indicate that the HMG box domain is dispensable for BBX association with this locus, suggesting that BBX may be recruited to the *Cdkn1a* (p21) promoter through its interaction with p53.

**Figure EV3:**
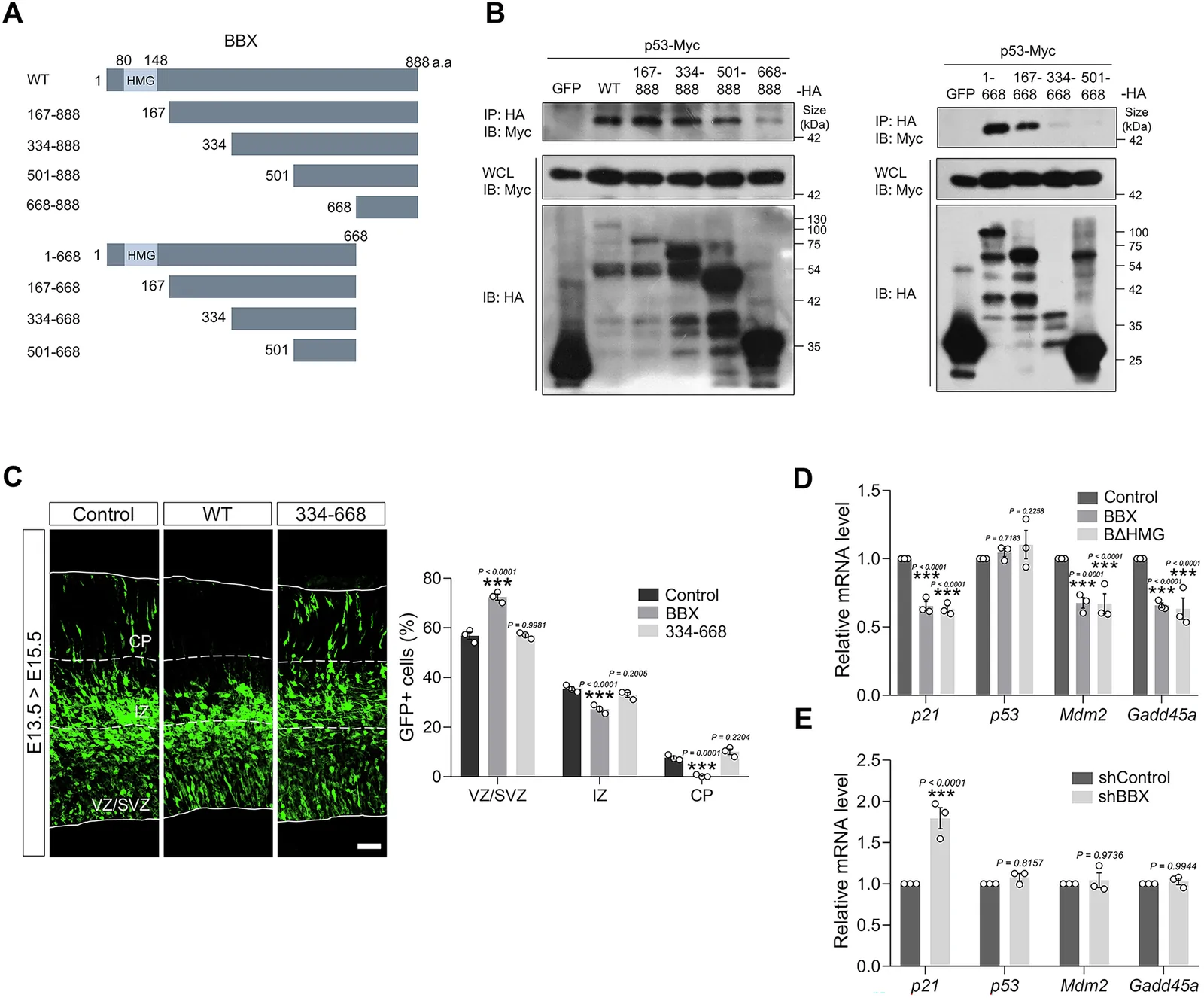
BBX function depends on its interaction with p53. (**A**) Schematic representation of wild-type BBX and the indicated BBX truncation mutants. Each mutant is named according to the amino acid residues retained in the construct. HMG box, high mobility group box domain. (**B**) CoIP analysis of p53 binding to BBX truncation mutants. Myc-tagged p53 was co-expressed with HA-tagged wild-type BBX or the indicated truncation mutants. HA immunoprecipitates were immunoblotted with an anti-Myc antibody. WCL, whole-cell lysate. (**C**) Distribution of GFP⁺ cells in embryonic cortices electroporated with control, wild-type BBX, or the BBX 334–668 mutant. Plasmids were introduced by in utero electroporation at E13.5, and brain sections were analyzed at E15.5. Solid white lines mark the apical and basal borders of the cortical tissue sections, whereas dashed white lines indicate the boundaries between the VZ/SVZ, IZ, and CP. The graph shows the distribution of GFP⁺ cells across cortical regions (*n* = 3). Scale bar, 25 μm. (**D**) qPCR analysis of mRNA levels of representative p53 target genes in BBX or BΔHMG -transduced NSCs. Cells were harvested 2 days after transduction, and mRNA levels of p21, p53, MDM2, and GADD45A were measured by qPCR (*n* = 3). (**E**) qPCR analysis of representative p53 target genes in NSCs transduced with shControl or shBBX lentivirus (*n* = 3). Data information: Data are presented as mean ± SEM from three biological replicates. Statistical significance was determined by the two-way ANOVA. ***P < 0.001. Source data are available online for this figure.

To directly test whether p53 binding is required for BBX function in cortical development, we constructed a series of truncation mutants of full-length BBX, an 888-amino-acid protein, to identify a p53-binding-defective BBX mutant (Fig. EV3A). The truncation mutants were named according to the amino acid residues retained in each construct. CoIP analysis identified the BBX 334–668 mutant as the largest retained fragment with little or no detectable p53-binding activity (Fig. EV3B). In vivo analysis showed that this p53-binding-defective mutant, unlike wild-type BBX, failed to promote VZ/SVZ retention of electroporated cells and instead exhibited a distribution pattern similar to that of control cells (Fig. EV3C). These results suggest that p53 binding is required for BBX-mediated regulation of cortical cell distribution during development.

Next, we examined whether BBX influences p53-dependent transcriptional activity using a luciferase reporter assay. Reporter plasmids contained either the wild-type *Cdkn1a* (p21) promoter or a mutant version in which two CATG sequences within the overlapping BBX ChIP-seq peak and p53 motif were replaced with GGGG (Fig. 4E). Luciferase reporter assays were performed in U87MG human glioblastoma cells. As shown in Fig. 4F, p53 overexpression strongly induced p21 promoter reporter activity, whereas co-expression of BBX suppressed this induction. To examine the effect of BBX knockdown in U87MG cells, we generated a human *BBX*-targeting shRNA vector, which reduced BBX expression by ~80% (Fig. 4G). Consistently, BBX knockdown enhanced p21 promoter reporter activity, whereas mutation of the p53-responsive element largely abolished this enhancement, suggesting that the effect of BBX on p21 transcription depends primarily on this element (Fig. 4H). Taken together, these findings indicate that BBX attenuates p53-dependent transcriptional activation of the p21 promoter.

We then performed ChIP-PCR and ChIP-qPCR in NSCs to determine whether BBX modulates p53 occupancy at the *Cdkn1a* (p21) promoter. BBX overexpression decreased p53 binding to the p21 promoter (Fig. 4I), whereas BBX depletion resulted in a pronounced increase in p53 occupancy at this site (Fig. 4J). These results indicate that BBX suppresses p53-dependent transcriptional activation of p21 by limiting p53 recruitment to the p21 promoter.

In line with the results of the luciferase and ChIP assays, qPCR analysis showed that BBX overexpression downregulated canonical p53 target genes in NSCs, including *Cdkn1a* (p21), *Mdm2* (Barak et al, 1993; Wu et al, 1993), and *Gadd45a* (Kastan et al, 1992). BΔHMG exhibited a comparable suppressive effect (Fig. EV3D). Among these genes, only *Cdkn1a* (p21) was upregulated upon BBX knockdown, further supporting p21 as a BBX-regulated p53 target gene under physiological conditions in NSCs (Fig. EV3E). Consistently, BBX-regulated changes in p21 protein expression were confirmed by western blot analysis (Fig. 4K,L). Next, we asked whether the 1,356 DEGs identified by RNA-seq analysis of BBX-knockdown NSCs included known p53 target genes. Using a curated p53 target gene set from MSigDB, we identified 23 DEGs corresponding to previously reported p53 target genes (Table EV1). These findings are consistent with the possibility that BBX depletion influences p53 transactivation activity at a subset of p53-responsive genes.

To examine whether the shBBX-induced increase in p21 expression observed in vitro is recapitulated in vivo, we performed in utero electroporation at E13.5 and analyzed p21 protein expression by immunostaining at E18.5. In shBBX-electroporated brains, the previously identified cell clusters in the intermediate zone (IZ) exhibited high levels of p21 expression (Fig. 4M). Further marker analysis revealed that the majority of these cells were DCX⁺ newborn neurons, with a small subset identified as more differentiated TBR1⁺ cells (Bulfone et al, 1995; Hevner et al, 2001) (Fig. 4N). These findings raise the possibility that aberrant p21 upregulation caused by BBX depletion disrupts the migration and morphological maturation of immature neurons, thereby contributing to their ectopic accumulation within the IZ. Collectively, these results indicate that BBX suppresses p53-dependent transcriptional activation of *Cdkn1a* (p21) by limiting p53 recruitment to the *Cdkn1a* promoter, thereby reducing p21 expression.

### BBX depletion promotes cell-cycle exit and senescence-associated changes in NSCs

**Figure 5:**
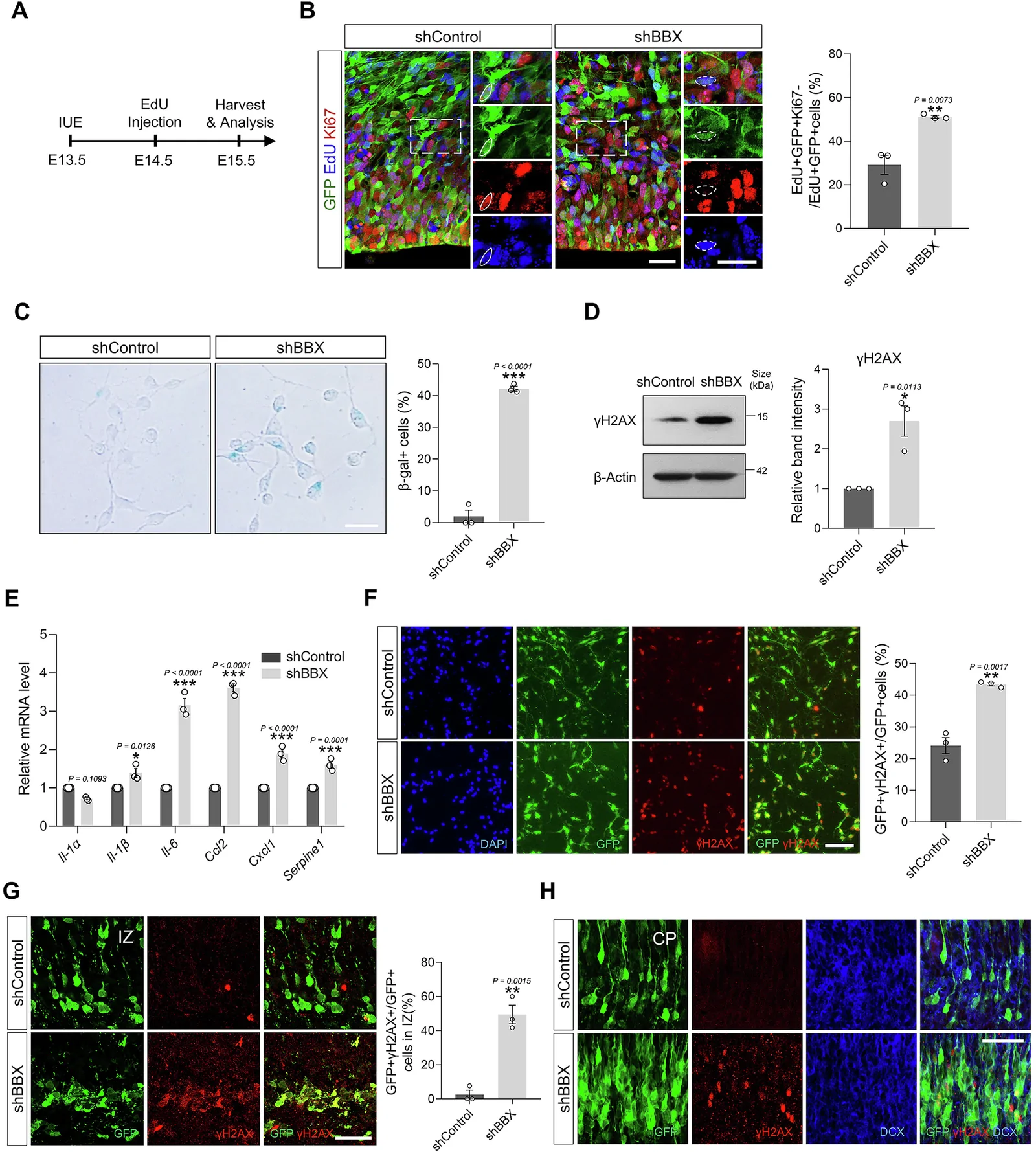
BBX knockdown accelerates cell cycle exit of NSCs and induces cellular senescence. (**A**) Schematic diagram illustrating the timeline of the cell cycle exit assay. In utero electroporation was performed at E13.5, followed by 5-ethynyl-2-deoxyuridine (EdU) injection at E14.5 and tissue harvest at E15.5 for analysis. (**B**) E15.5 cortices electroporated with shBBX at E13.5, followed by EdU injection 1 day later, and immunostained for GFP (green), Ki67 (red), and EdU (blue). The regions marked by dashed boxes are magnified and displayed in the right column. In the right column of the shControl sample, the solid-lined circle highlights a representative EdU⁺GFP⁺Ki67⁺ cell, while in the shBBX sample, the dotted-lined circle marks a Ki67− cell within the EdU⁺GFP⁺ population. Scale bars, 25 μm. The graph quantifies the proportion of cells that exited the cell cycle (*n* = 3). (**C**) Senescence-associated β-galactosidase (SA-β-gal) staining of NSCs expressing shBBX. Cells exhibiting senescence are marked by blue cytoplasmic staining. Scale bar, 50 μm. The graph quantifies the percentage of β-gal⁺ cells (*n* = 3). (**D**) Western blot analysis of γH2AX protein levels in shBBX-transduced NSCs under proliferation conditions. Cells were harvested 3 days after lentiviral transduction. The graph shows quantification of relative γH2AX band intensity (*n* = 3). (**E**) qPCR analysis of senescence-associated secretory phenotype (SASP) genes in NSCs transduced with shBBX lentiviral vectors (*n* = 3). (**F**) Immunostaining for γH2AX in BBX-depleted NSCs under differentiation conditions. shBBX-transduced NSCs were cultured without fibroblast growth factor 2 (FGF2) for 2 days and immunostained for GFP (green) and γH2AX (red). Nuclei were counterstained with DAPI (blue). Scale bar, 100 μm. The graph shows the frequency of γH2AX⁺ cells among GFP⁺ cells (*n* = 3). (**G**, **H**) Immunostaining for γH2AX in E18.5 mouse cortices electroporated with shBBX at E13.5. Images show GFP (green) and γH2AX (red) staining in the IZ (**G**), and GFP (green), γH2AX (red), and DCX (blue) staining in the CP (**H**). The graph in (**G**) shows the frequency of γH2AX⁺ cells among GFP⁺ cells in the IZ (*n* = 3). Scale bars, 50 μm. Data information: Data are presented as mean ± SEM from three independent biological replicates. Statistical significance was determined by the unpaired Student’s *t* test (**B**–**D**, **F**, **G**) and two-way ANOVA (**E**). *P < 0.05, **P < 0.01 ***P < 0.001. Source data are available online for this figure.

Next, we investigated whether the aberrant phenotypes induced by BBX knockdown are functionally associated with increased p21 expression. Given that p21 is a well-established cell-cycle inhibitor, we first examined whether p21 upregulation affects cell-cycle dynamics in BBX-depleted NSCs. To test this possibility, we performed in utero electroporation of shBBX plasmids at E13.5, followed by EdU pulse labeling (Salic and Mitchison, 2008) 24 h later (Fig. 5A). Samples were collected at E15.5 and immunostained for EdU and Ki67, a cell proliferation marker (Gerdes et al, 1984). EdU⁺Ki67⁺ cells correspond to progenitors that remained proliferative at the time of harvest, whereas EdU⁺Ki67⁻ cells represent cells that had exited the cell cycle (Siegenthaler and Miller, 2005; Kwon et al, 2023). As shown in Fig. 5B, BBX knockdown increased the proportion of EdU⁺Ki67⁻ cells, indicating enhanced cell-cycle exit.

p21 is also well recognized as a key mediator of cellular senescence (Brown et al, 1997; Vogt et al, 1998; Fang et al, 1999). To examine whether BBX knockdown induces senescence in NSCs, shBBX-transduced E14.5 NSCs were seeded on poly-L-ornithine- and laminin-coated plates and maintained for 2 days in proliferation medium supplemented with fibroblast growth factor 2 (FGF2). Senescence-associated β-galactosidase (SA-β-gal) staining revealed that positive cells were rarely detected in control samples, whereas more than 40% of cells in the shBBX group showed detectable β-gal activity (Fig. 5C). Consistent with the increase in SA-β-gal^+^ cells, western blot analysis showed that γH2AX, another well-established marker associated with cellular senescence (Bernadotte et al, 2016), was increased at the protein level in shBBX-transduced NSCs maintained under proliferation conditions (Fig. 5D). Further supporting this senescence-associated phenotype, BBX-depleted NSCs exhibited increased mRNA expression of genes associated with the senescence-associated secretory phenotype (SASP), including *Il-6*, *Ccl2*, *Cxcl1*, and *Serpine1* (Fig. 5E). We also assessed whether BBX-depleted NSCs also exhibit senescence-associated changes under differentiation conditions in vitro. NSCs were cultured in differentiation medium lacking growth factors for 2 days and immunostained for γH2AX. Consistent with the increase observed under proliferation conditions, BBX-depleted NSCs displayed increased γH2AX^+^ foci under differentiation conditions (Fig. 5F).

We next asked whether this increase in γH2AX also occurs in vivo under the E13.5 > E18.5 electroporation setting, in which shBBX-transduced cells showed abnormal cluster accumulation in the IZ. As shown in Fig. 5G, the frequency of γH2AX⁺ cells was substantially higher among shBBX-transduced cells accumulated in the IZ than among control GFP⁺ cells located in the IZ. In addition, γH2AX⁺ cells were also detected in the middle CP of shBBX-electroporated brains (Fig. 5H). Together, these findings indicate that BBX knockdown promotes senescence-associated changes in NSCs in vitro and induces both neural progenitor cell-cycle exit and a senescence-associated DNA damage response in cortical cells in vivo. These phenotypes are consistent with the known effects of elevated p21 expression.

### p21 acts as a functional downstream mediator of BBX during brain development

**Figure 6:**
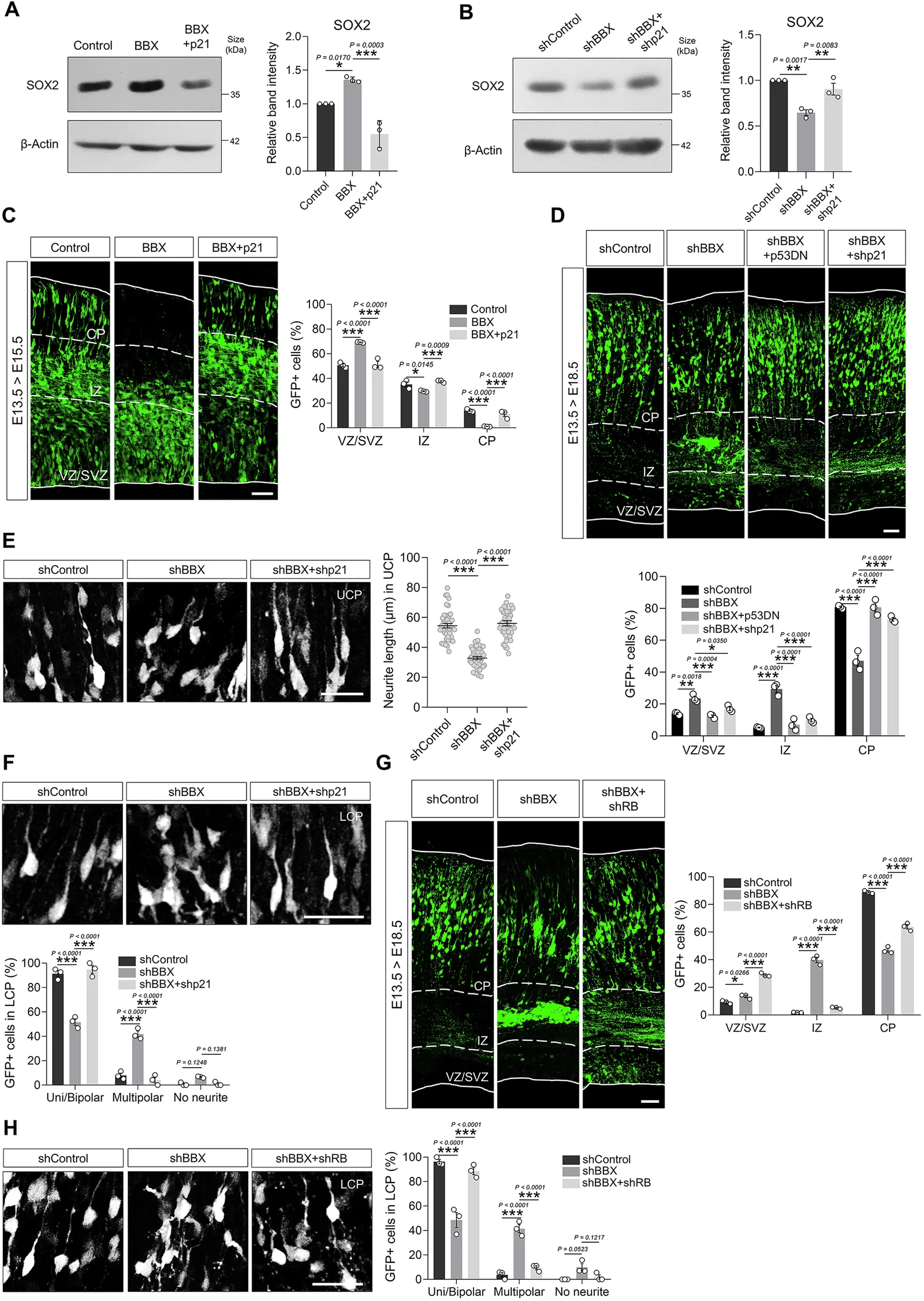
p21 mediates key BBX-dependent phenotypes in NSCs. (**A**) Western blot analysis of SOX2 expression in BBX-expressing NSCs with or without p21 co-transduction. The graph shows western blot quantification (*n* = 3). (**B**) Western blot analysis of SOX2 expression in shBBX-transduced NSCs with or without p21 co-knockdown. The graph shows western blot quantification (*n* = 3). (**C**) GFP immunostaining of E15.5 cortices electroporated at E13.5 with a BBX expression vector, with or without co-electroporation of p21. Scale bar, 50 μm. The graph shows immunostaining quantification (*n* = 3). (**D**) GFP immunostaining of E18.5 cortices electroporated at E13.5 with shBBX vectors alone or together with either a dominant-negative p53 expression vector (p53DN) or a p21 knockdown vector (shp21). Scale bar, 50 μm. The lower graph shows immunostaining quantification (*n* = 3). (**E**) GFP immunostaining of the UCP in E18.5 brains electroporated at E13.5 with shBBX alone or in combination with p21 knockdown. Scale bar, 50 μm. The graph shows immunostaining quantification (*n* = 45 cells from 3 biological replicates). (**F**) GFP immunostaining in the LCP in E18.5 brains electroporated at E13.5 with shBBX, with or without shp21 vector. Scale bar, 50 μm. The graph shows immunostaining quantification (*n* = 3). (**G**) GFP immunostaining of E18.5 cortices electroporated at E13.5 with shBBX vector alone or with Rb knockdown vector (shRB). Scale bar, 50 μm. The graph shows immunostaining quantification (*n* = 3). (**H**) GFP immunostaining in the LCP in E18.5 brains electroporated at E13.5 with shBBX, with or without shRB vector. Scale bar, 50 μm. The graph shows immunostaining quantification (*n* = 3). In (**C**, **D**, **G**), solid white lines mark the apical and basal borders of the cortical tissue sections, whereas dashed white lines indicate the boundaries between the VZ/SVZ, IZ, and CP. Data information: Data are presented as mean ± SEM from three independent biological replicates. Statistical significance was determined by the one-way ANOVA (**A**, **B**, **E**) and two-way ANOVA (**C**, **D**, **F**–**H**). *P < 0.05, **P < 0.01, ***P < 0.001. Source data are available online for this figure.

**Figure EV4:**
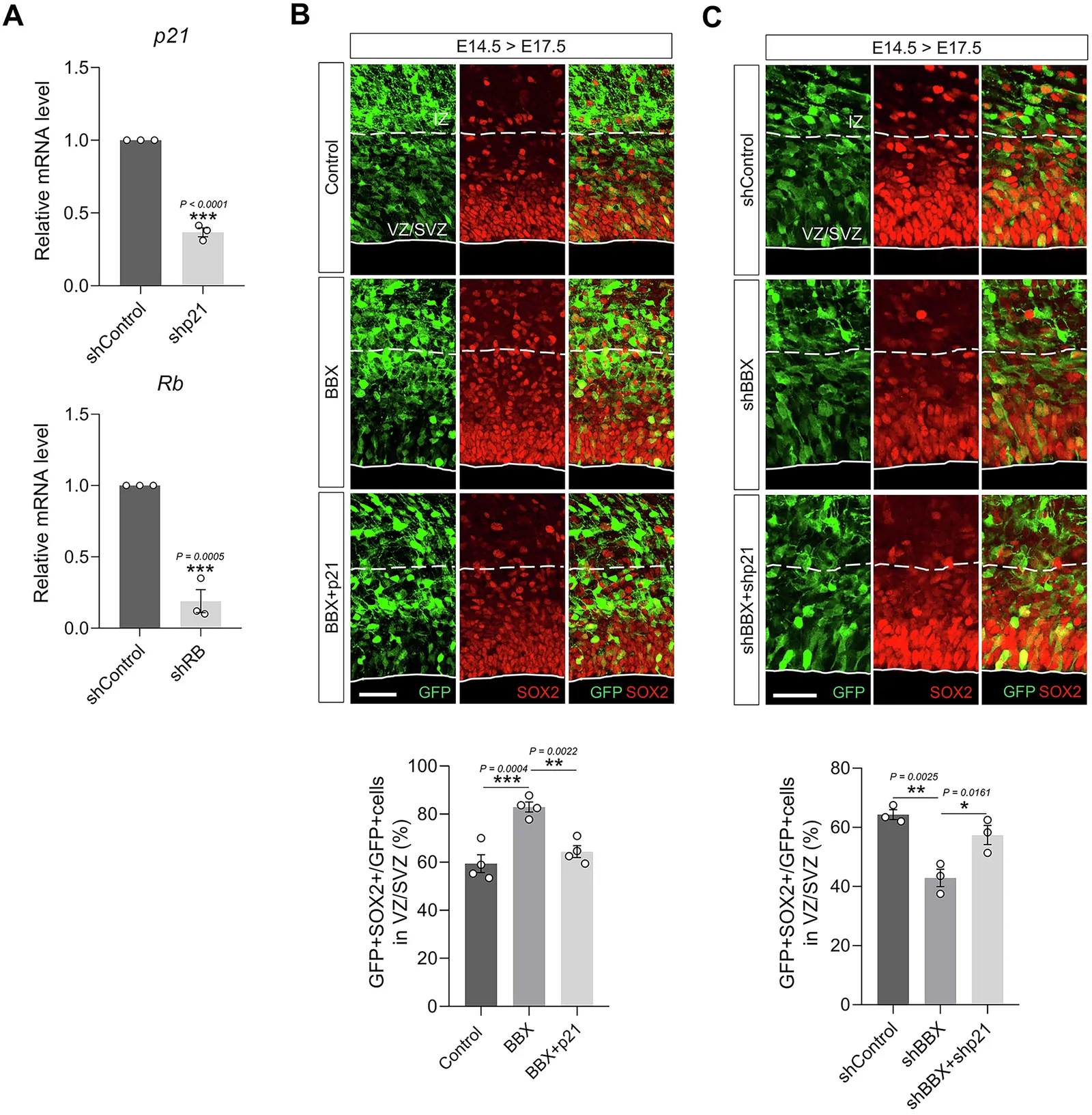
p21 mediates BBX-dependent regulation of SOX2⁺ progenitor cells in vivo. (**A**) qPCR analysis of *Cdkn1a* (p21) and *Rb* mRNA levels in NSCs transduced with the indicated lentiviral shRNAs. Cells were harvested 2 days after transduction (*n* = 3). (**B**) GFP and SOX2 immunostaining in embryonic cortices electroporated with control, BBX, or BBX and p21 expression plasmids. Plasmids were introduced by in utero electroporation at E14.5, and brain sections were analyzed at E17.5. The graph shows the percentage of GFP⁺SOX2⁺ cells among GFP⁺ cells in the VZ/SVZ (*n* = 4). Scale bar, 50 μm. (**C**) SOX2 immunostaining in embryonic cortices electroporated with shControl, shBBX, or shBBX plus shp21 plasmids. Plasmids were introduced by in utero electroporation at E14.5, and brain sections were analyzed at E17.5. The graph shows the percentage of GFP⁺SOX2⁺ cells among GFP⁺ cells in the VZ/SVZ (*n* = 3). Scale bar, 50 μm. In (**B**, **C**), solid white lines mark the apical border of the cortical tissue sections, whereas dashed white lines indicate the boundaries between the VZ/SVZ and IZ. Data information: Data are presented as mean ± SEM from at least three biological replicates. Statistical significance was determined by the unpaired Student’s *t* test (**A**) and one-way ANOVA (**B**, **C**). *P < 0.05, **P < 0.01, ***P < 0.001. Source data are available online for this figure.

Finally, we directly modulated p21 expression to determine whether it functionally mediates BBX-dependent regulation of NSC properties and cortical development. In vitro, western blot analysis showed that SOX2 upregulation induced by BBX overexpression was suppressed by p21 co-expression (Fig. 6A). We also co-depleted p21 using a p21-targeting shRNA (shp21), which reduced *Cdkn1a* (p21) mRNA levels by more than 50% (Fig. EV4A). p21 co-depletion reversed the reduction in SOX2 expression induced by BBX knockdown (Fig. 6B).

In vivo, under the E13.5 > E15.5 electroporation setting, p21 co-expression reversed the distribution shift of BBX-overexpressing cells, reducing their enrichment in the VZ/SVZ and restoring their CP localization to a pattern comparable to that of control cells (Fig. 6C). Consistently, under the E14.5 > E17.5 electroporation setting, SOX2 immunostaining revealed that p21 co-expression also reduced the BBX-induced increase in the proportion of SOX2⁺ cells among GFP⁺ cells within the VZ/SVZ (Fig. EV4B). To further test whether activation of the p53–p21 pathway mediates the BBX knockdown phenotype in vivo, we co-electroporated shBBX with either a dominant-negative p53 construct, originally designated p53DD and hereafter referred to as p53DN, or shp21. p53DN inhibits endogenous p53 function by interfering with wild-type p53 DNA binding through heterotetramerization (Shaulian et al, 1992; Irwin et al, 2000). In the E13.5 > E18.5 setting, co-electroporation with either p53DN or shp21 rescued the abnormal laminar distribution of BBX-knockdown cells (Fig. 6D). Notably, the cell clusters observed in the IZ of shBBX brains were no longer evident in either the p53DN or shp21 co-electroporated samples, supporting the idea that aberrant clustering of immature neurons results from p21 upregulation. Closer examination revealed that p21 knockdown also rescued the morphological defects caused by BBX depletion, including shortened neurite length in upper CP migratory neurons (Fig. 6E) and the abnormally increased neurite number of lower CP neurons (Fig. 6F), restoring both parameters to control levels. In addition, under the E14.5 > E17.5 setting, p21 co-knockdown restored the BBX knockdown-induced reduction in the proportion of SOX2⁺ cells within the VZ/SVZ to a level comparable to that of control cells (Fig. EV4C).

Postmitotic neurons located outside the VZ/SVZ have already exited the cell cycle. Therefore, the defects observed in BBX-depleted postmitotic neurons are unlikely to be explained solely by the cell-cycle-inhibitory function of p21, suggesting that p21-induced senescence-associated changes may contribute to these phenotypes. To test this hypothesis, we co-depleted retinoblastoma protein (RB) and BBX using shRNA vectors, as RB serves as a central mediator of p21-induced senescence (Engeland, 2022). We confirmed that the *Rb*-targeting shRNA vector (shRB) reduced *Rb* mRNA levels by nearly 80% (Fig. EV4A). As shown in Fig. 6G, co-depletion of RB together with BBX substantially reduced the abnormal cell clusters in the IZ and promoted neuronal migration toward the upper cortical layers. RB co-depletion also ameliorated the polarity defects of BBX-knockdown neurons, increasing the proportion of uni/bipolar cells while reducing the proportion of multipolar cells in the lower CP (Fig. 6H). These findings suggest that senescence-associated changes contribute to the multiple abnormalities observed in BBX-depleted postmitotic neurons.

**Figure EV5:**
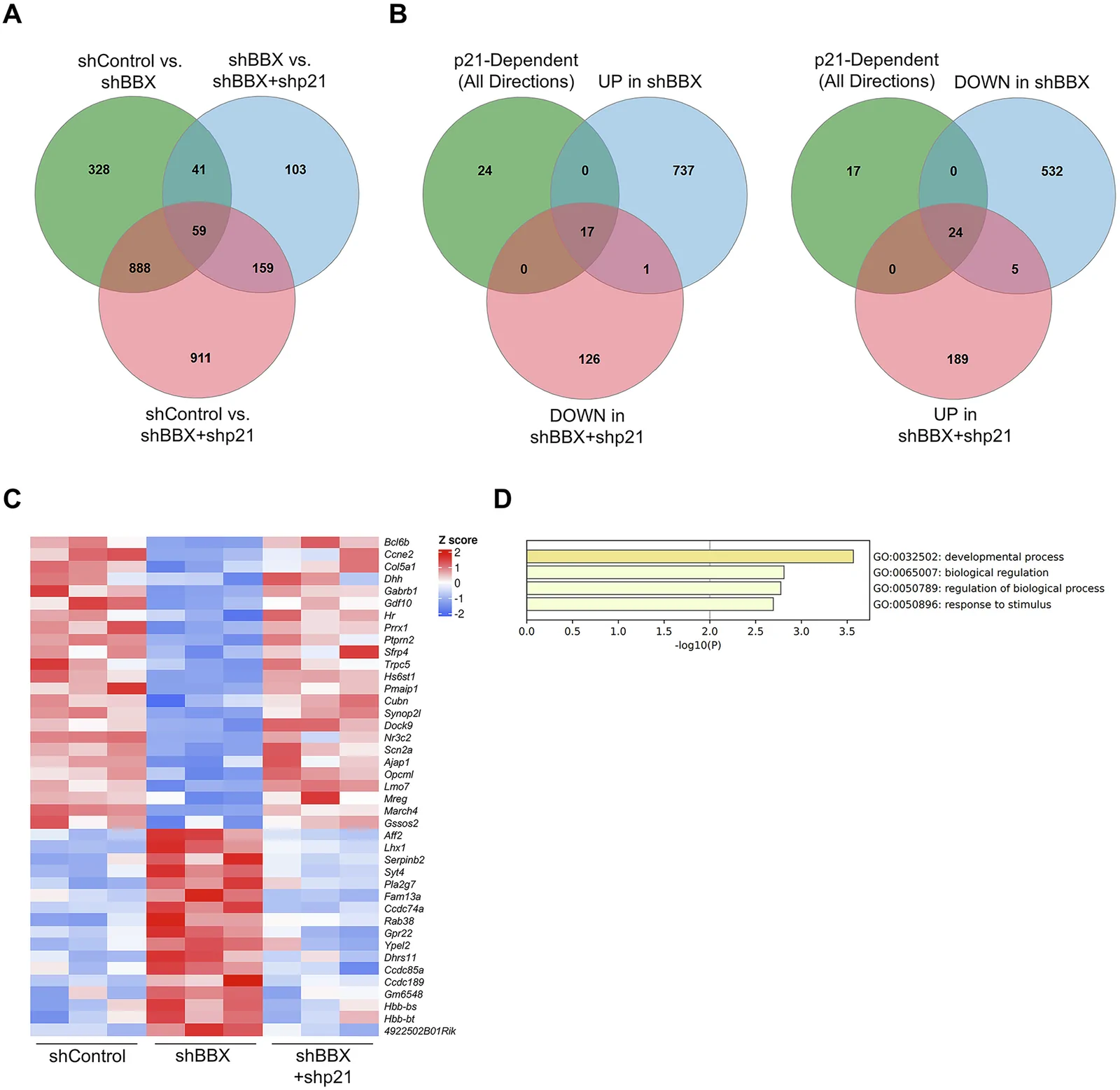
RNA-seq-based analysis of the p21 dependency of BBX knockdown-regulated genes. (**A**) Venn diagram showing the overlap of DEGs identified from the indicated pairwise comparisons of RNA-seq samples. DEGs were defined using DESeq2 with the Wald test as those with an absolute fold change ≥1.3 and Benjamini–Hochberg-adjusted *P* < 0.05 (*n* = 3). (**B**) Venn diagrams showing p21-dependent BBX-regulated DEGs. BBX-regulated DEGs whose expression changes were reversed by shp21 co-knockdown were classified as p21-dependent DEGs. The left diagram shows genes upregulated by shBBX and downregulated by shp21 co-knockdown, whereas the right diagram shows genes downregulated by shBBX and upregulated by shp21 co-knockdown. (**C**) Heatmap showing the expression patterns of p21-dependent BBX-regulated DEGs across the rescue RNA-seq conditions. Expression values are shown as Z-scores. (**D**) GO functional enrichment analysis of p21-dependent BBX-regulated DEGs. Enriched GO terms were identified using Metascape based on the cumulative hypergeometric test. Bars indicate −log10(*P*), and the corresponding GO identifiers and term names are listed on the right.

Finally, we examined which BBX-regulated transcriptional changes are p21-dependent. To this end, we performed rescue RNA-seq analysis using NSCs transduced under a dual-vector design: shControl + shControl, shBBX + shControl, or shBBX + shp21. The same dual-vector strategy was used throughout this study for experiments involving simultaneous manipulation of two effectors, allowing the input of the shBBX or BBX vector to be matched between the corresponding control and co-manipulation conditions. In the rescue RNA-seq experiment, this design allowed the shBBX viral input to be matched between the shBBX + shControl and shBBX + shp21 conditions. However, compared with the initial single-vector shControl versus shBBX RNA-seq experiment, the shBBX + shControl condition in this rescue RNA-seq design would have received a lower proportion of shBBX virus, because the total viral input was divided between two vectors. This difference may have attenuated the overall magnitude of BBX knockdown-induced transcriptional changes. Moreover, because the expression of some BBX-responsive genes may not change linearly with BBX levels but instead respond only when BBX expression falls below a specific threshold, the reduced shBBX input could also have altered the composition of the DEG set itself. Therefore, for this comparative rescue analysis, we used a cutoff of an absolute fold change ≥ 1.3 and adjusted *P* < 0.05. This analysis classified candidate BBX-regulated DEGs into 41 p21-dependent and 328 p21-independent genes (Fig. EV5A; Dataset EV1). Among the 41 p21-dependent DEGs, 17 were upregulated by shBBX and reduced by shp21, whereas 24 were downregulated by shBBX and restored by shp21 (Fig. EV5B). Figure EV5C shows a heatmap of the 41 p21-dependent DEGs across the three RNA-seq conditions. GO functional enrichment analysis of these genes revealed enrichment of terms related to developmental processes, biological regulation, and responses to stimuli (Fig. EV5D). Together, these findings indicate that p21 mediates a specific subset of BBX-regulated transcriptional changes while serving as an important functional mediator of the cellular phenotypes induced by BBX depletion.

## Discussion

In this study, we show that insufficient expression of BBX during the mid-neurogenic stage of brain development leads to premature cell-cycle exit of NSCs in the VZ and disrupted migration patterns of newborn neurons. We further demonstrate that these phenotypes result from an increase in p53-dependent p21 gene expression. Notably, we found that the BBX mutant lacking the HMG box domain and showing impaired chromatin association retained full activity across all experimental settings tested, comparable to that of the wild-type protein. Although this study initially aimed to clarify the role of BBX as a transcription factor due to its HMG box domain, our results demonstrate that BBX functions in brain development not through HMG box-dependent chromatin association, but through interaction with p53. Thus, it is more appropriate to refer to BBX as a transcriptional regulator rather than a canonical transcription factor, at least in the context of mammalian brain development.

The function of BBX as a cell cycle regulator was first identified in fission yeast (Sánchez-Díaz et al, 2001). In that study, BBX was shown to promote cell cycle progression by enhancing the G1/S phase transition, but there are a few important differences between their findings and ours. Specifically, while they stressed the contribution of the HMG box domain in cell cycle regulation, our results in the mammalian system demonstrate that the HMG box domain is dispensable for brain development. Moreover, they did not identify specific downstream target genes or interacting partners of BBX, and thus did not provide a detailed mechanism for how BBX acts as a cell cycle progressor.

Given that proliferative capacity is a defining feature of the NSCs, the cell cycle regulator p21 has been a focal point of numerous neurodevelopmental studies. For example, forebrain NSCs isolated from adult p21^−/−^-mice exhibit higher proliferation rates compared to wild-type controls (Kippin et al, 2005). More recently, Dai et al demonstrated that p21 acts as a downstream target of the nucleoporin SEH1 by showing that p21 knockdown partially rescued the impaired proliferation of *Seh1*^−/−^ NSCs (Dai et al, 2024). As exemplified by these findings, experimental approaches testing whether p21 is a major effector in brain development have primarily emphasized its role in cell proliferation. In this context, our study reveals p21-induced effects that extend beyond the reduction of cell proliferation, namely, cellular senescence. Specifically, we show that shBBX-induced p21 upregulation drives senescence-associated changes, as evidenced by increased expression of senescence-related gene signatures in our RNA-seq analysis and by elevated β-gal activity and γH2AX levels in shBBX-expressing NSCs. Consistent with these in vitro findings, γH2AX^+^ cells were prominently observed among shBBX-electroporated cells in the developing cortex, whereas they were rarely observed in control cells. Functionally, depletion of RB, a central mediator of p21-induced senescence, restored the abnormal cell morphology and positioning defects of BBX-depleted newborn neurons in vivo.

Given the known function of p21, the in vivo phenotypes observed upon BBX knockdown in the developing cortex, including reduced SOX2⁺ progenitor populations in the VZ/SVZ, decreased Cyclin D1 expression, and increased cell-cycle exit, can be readily linked to activation of a p21-dependent cell-cycle inhibitory program. In contrast, the reduced generation of DCX⁺ neurons from BBX-depleted NSCs in the in vitro differentiation assay (Fig. EV1I) may appear, at first glance, to be inconsistent with the expected consequence of enhanced cell-cycle exit. This apparent discrepancy may be explained by the specific conditions of the in vitro differentiation assay. In this assay, growth factor withdrawal suppresses proliferation and promotes differentiation in both shControl and shBBX samples. Thus, the culture condition drives both groups toward a cell-cycle-exited differentiation state, which may mask the additional cell-cycle-inhibitory effect of shBBX-induced p21 activation. Instead, the senescence-associated state induced by BBX knockdown may become the dominant factor affecting neuronal differentiation. Indeed, as shown in Fig. 5C, a substantial fraction of BBX-depleted NSCs already exhibited detectable senescence-associated β-gal activity under proliferation conditions. Such a compromised cellular state may disrupt multiple cellular programs required for efficient generation of DCX⁺ neurons and subsequent neurite maturation (Figs. EV1I and  1L).

In the E14.5 > E17.5 setting, both BBX overexpression and knockdown shifted the distribution of GFP⁺ cells away from the CP and toward the VZ/SVZ, although this redistribution was substantially more pronounced following BBX overexpression. However, marker analyses revealed that these superficially similar distributional phenotypes reflected distinct cellular states. BBX overexpression increased the proportions of SOX2⁺ and PAX6⁺ progenitor-marker-positive cells in the VZ/SVZ while reducing SATB2⁺ upper-layer neurons, suggesting progenitor retention and/or expansion with delayed neuronal differentiation or CP entry. In contrast, BBX knockdown reduced SOX2⁺, Cyclin D1⁺, and PAX6⁺ cells within the GFP⁺ VZ/SVZ population, indicating that the accumulation of BBX-depleted cells in the VZ/SVZ does not reflect enhanced progenitor maintenance. Rather, these findings raise the possibility that BBX-depleted cells undergo premature cell-cycle exit but fail to migrate efficiently out of the progenitor zone, consistent with the broader migration defects observed in newborn neurons upon BBX knockdown. Notably, BBX knockdown did not significantly increase the proportion of SATB2⁺ cells, suggesting that BBX depletion does not simply promote neuronal differentiation. This may reflect opposing consequences of p21 activation: increased cell-cycle exit could facilitate aspects of neuronal differentiation, whereas senescence-associated changes might impair maturation of DCX⁺ neurons toward a SATB2⁺ upper-layer identity, thereby contributing to the limited net change in SATB2⁺ cells.

p21 was first identified as a transcriptional target of p53 (el-Deiry et al, 1993) and has since been widely recognized as one of its canonical downstream effectors. However, p53-independent expression of p21 has also been reported, playing important roles in various cellular events and normal tissue development (Sheikh et al, 1994; Macleod et al, 1995; Jung et al, 2010). For example, in the aforementioned study on SEH1-mediated p21 regulation, p53 was found to be dispensable (Dai et al, 2024). In this study, we show that BBX associates with p53, that there is an inverse correlation between BBX expression levels and the amount of p53 recruited to the p21 promoter, and that the shBBX-induced cell clusters in the IZ disappear upon expression of p53DN, similar to what is seen with p21 knockdown. In this respect, our findings provide another example of a p53-dependent mechanism underlying the biological effects of p21.

As a transcription factor, p53 governs a wide array of cellular processes, including cell-cycle arrest, DNA repair, and apoptosis, by regulating an extensive pool of target genes (Fischer, 2017; Wang et al, 2023). Thus, given that BBX interacts with p53, we could not rule out the possibility that other downstream effectors of p53, in addition to p21, might also contribute to the functional outcomes of BBX. Indeed, our RNA-seq analysis of BBX-knockdown NSCs revealed that 23 DEGs corresponded to previously reported p53 target genes. However, most of the major phenotypic alterations observed in this study upon either BBX overexpression or knockdown were largely reversed by modulating p21 expression. Specifically, BBX knockdown-induced defects, such as altered neuronal migration patterns within the cortical laminar structure, abnormal morphology, and shortened neurites of migrating neurons, were restored to near-normal levels upon p21 co-suppression. These results indicate that, among the numerous p53 target genes, p21 serves as an important functional downstream mediator of key BBX-dependent phenotypes during brain development.

Through cross-validation of our transcriptome profiling data with the ChIP-seq dataset archived in GEO (accession no. GSE64028) (Lee et al, 2019), we identified p21 as a putative transcriptional target regulated by BBX. It is worth noting that the GEO dataset was generated from trophoblast stem cells, which arise during the earliest phases of embryogenesis and represent a cellular state far upstream of the neurogenic period analyzed in our study. The fact that BBX is recruited to the p21 promoter even in such an early embryonic cell type suggests that BBX may serve as an essential transcriptional cofactor that positively regulates cell proliferation not only during the neurogenic period but also across broader stages of development. To further substantiate this possibility, future research should determine whether BBX represses p21 expression in trophoblast or embryonic stem cells and evaluate the impact of this repression on the proliferative capacity of early embryonic cells. In parallel, BBX expression should be assessed within adult neurogenic niches, to examine if it is expressed, and elucidate how its regulation might influence adult NSC behavior.

## Methods

**Table:** Reagents and tools table

Reagent/resource	Reference or source	Identifier or catalog number
**Experimental models**		
Mouse: ICR	Koatech	Hsd:ICR(CD-1®)
Lenti-X ™ 293 T Cell Line	Takara	Cat #632180
U87MG	ATCC	Cat #HTB-14
**Recombinant DNA**		
T7-p53DD-pcDNA3	Addgene	Cat #25989
psPAX2	Addgene	Cat #12260
pMD2.G	Addgene	Cat #12259
pLKO.3 G shBBX #1	This study	N/A
pLKO.3 G shBBX #2	This study	N/A
pLKO.3 G shBBX (human)	This study	N/A
pLKO.3 G shp21	This study	N/A
pLKO.3 G shRB	This study	N/A
pWPI BBX	This study	N/A
pWPI BBX (167-888)	This study	N/A
pWPI BBX (334–888)	This study	N/A
pWPI BBX (501–888)	This study	N/A
pWPI BBX (668–888)	This study	N/A
pWPI BBX (1–668)	This study	N/A
pWPI BBX (167–668)	This study	N/A
pWPI BBX (334–668)	This study	N/A
pWPI BBX (501–668)	This study	N/A
pWPI p53	This study	N/A
pWPI p21	This study	N/A
pCAGIG BBX	This study	N/A
pCAGIG BBX (334–668)	This study	N/A
pCAGIG p21	This study	N/A
pGL3B p21p-wt	This study	N/A
pGL3B p21p-mt	This study	N/A
**Antibodies**		
Rabbit anti-Sox2	Cell Signaling Technology	Cat #23064S (WB, IF-1:1000)
Rabbit anti-Cleaved caspase 3	Cell Signaling Technology	Cat #9661S (WB- 1:500)
Rabbit anti-HA tag	Cell Signaling Technology	Cat #3724S (WB- 1:1,000, ChIP- 1:50)
Mouse anti-p53	Cell Signaling Technology	Cat #2524S (WB- 1:300, ChIP- 1:50, IP- 1:50)
Rabbit anti-Gamma H2AX	Cell Signaling Technology	Cat #9718S (WB- 1:300, IF- 1:100)
Rabbit anti-H3	Cell Signaling Technology	Cat #4620S (WB- 1:5000)
Rabbit anti-Lamin A/C	Cell Signaling Technology	Cat #2032S (WB- 1:1000)
Anti-Mouse IgG (Light Chain Specific)-HRP	Cell Signaling Technology	Cat #58802S (WB- 1:10,000)
Anti-Rabbit IgG-HRP	Santa Cruz Biotechnology	Cat #sc-2357 (WB- 1:10,000)
Anti-Mouse IgG-HRP	Santa Cruz Biotechnology	Cat #sc-516102 (WB- 1:10,000)
Mouse anti-Beta actin	Santa Cruz Biotechnology	Cat #sc-47778 (WB- 1:20,000)
Mouse anti-Myc tag	Santa Cruz Biotechnology	Cat #sc-40 x (IP- 1:50)
Mouse anti-BBX	Santa Cruz Biotechnology	Cat #sc-377041 (WB- 1:500, IP- 1:50)
Chicken anti-GFP	Abcam	Cat #ab13970 (IF- 1:1000)
Rabbit anti-DCX	Abcam	Cat #ab18723 (IF- 1:1000)
Rabbit anti-Ki67	Abcam	Cat #ab15580 (IF- 1:1000)
Rabbit anti-TBR2	Abcam	Cat #ab23345 (IF- 1:1000)
Rabbit anti-SATB2	Abcam	Cat #ab92446 (IF- 1:100)
Guinea Pig anti-DCX	Merck	Cat #AB2253 (IF- 1:500)
Rabbit anti-PAX6	Invitrogen	Cat #42-6600 (IF- 1:200)
Rabbit anti-CyclinD1	Invitrogen	Cat #MA5-14512 (WB- 1:1000, IF- 1:1000)
Anti-Rabbit Alexa Fluor 555	Invitrogen	Cat #A31572 (IF- 1:1000)
Anti-Chicken Alexa Fluor 488	Invitrogen	Cat #A11039 (IF- 1:1000)
Anti-Guinea Pig Alexa Fluor 647	Invitrogen	Cat #A21450 (IF- 1:1000)
**Oligonucleotides and other sequence-based reagents**		
**shRNAs (gene target)**		**Sequence**
BBX #1 (Mouse)		GGACTTGCAGGCCTGATATTT
BBX #2 (Mouse)		TGTGCCATCCTCACGGAATTA
p21 (Mouse)		GCAGACCAGCCTGACAGATTT
RB (Mouse)		ATTGAAGAACCCGAATTTATT
BBX (Human)		TGGAAGAATCAAAGCTAATAA
**Cloning primers**	**Forward**	**Reverse**
BBX	ACGCGTGAATTCGCCACCATGAAAGGCAGTAATAGAAATAA	GGATCCGCGGCCGCTCAAGCGTAATCTGGAACATCGTATGGGTACTGGTCAGCACAAGAAATAA
BBX (167-888)	ACGCGTGAATTCGCCACCATGAAGAAACTTTGGGCCTTCCCACCTG	GGATCCGCGGCCGCTCAAGCGTAATCTGGAACATCGTATGGGTACTGGTCAGCACAAGAAATAA
BBX (334–888)	ACGCGTGAATTCGCCACCATGGAAGAGAGAGGCACAGAAGTGGAGA	GGATCCGCGGCCGCTCAAGCGTAATCTGGAACATCGTATGGGTACTGGTCAGCACAAGAAATAA
BBX (501–888)	ACGCGTGAATTCGCCACCATGGGAGAAACCCCAAGAAAGAAAGTCC	GGATCCGCGGCCGCTCAAGCGTAATCTGGAACATCGTATGGGTACTGGTCAGCACAAGAAATAA
BBX (668–888)	ACGCGTGAATTCGCCACCATGAAGAAGTTTAAGAAGAAACTAAGGG	GGATCCGCGGCCGCTCAAGCGTAATCTGGAACATCGTATGGGTACTGGTCAGCACAAGAAATAA
BBX (1–668)	ACGCGTGAATTCGCCACCATGAAAGGCAGTAATAGAAATAA	GGATCCGCGGCCGCTCAAGCGTAATCTGGAACATCGTATGGGTAGCTCCCACTGGTCCCACTCT
BBX (167–668)	ACGCGTGAATTCGCCACCATGAAGAAACTTTGGGCCTTCCCACCTG	GGATCCGCGGCCGCTCAAGCGTAATCTGGAACATCGTATGGGTAGCTCCCACTGGTCCCACTCT
BBX (334–668)	ACGCGTGAATTCGCCACCATGGAAGAGAGAGGCACAGAAGTGGAGA	GGATCCGCGGCCGCTCAAGCGTAATCTGGAACATCGTATGGGTAGCTCCCACTGGTCCCACTCT
BBX (501–668)	ACGCGTGAATTCGCCACCATGGGAGAAACCCCAAGAAAGAAAGTCC	GGATCCGCGGCCGCTCAAGCGTAATCTGGAACATCGTATGGGTAGCTCCCACTGGTCCCACTCT
BΔHMG-mutagenesis	AGATGATGAGTCACCTGAGCAGCGATGGTGCCCTACCACAAACAAGCCTG	CAGGCTTGTTTGTGGTAGGGCACCATCGCTGCTCAGGTGACTCATCATCT
p53	ACGCGTGCCACCATGGAGGAGCCGCAGTCAGA	GGATCCTCACAGATCCTCTTCTGAGATGAGTTTTTGTTCCAAGTCCTCTTCAGAAATGA
p21	ACGCGTGAATTCGCCACCATGTCCAATCCTGGTGATGTCCGAC	GGATCCGCGGCCGCTCACGTAGAATCGAGACCGAGGAGAGGGTTAGGGATAGGCTTACCGGGTTTTCTCTTGCAGAAGACCAAT
p21p-wt	ACGCGTCTATCCTGACCCTCGTGCTTAGACCA	ACGCGTACGGACGCTCAGACCCACA
p21 (p53REmut)-mutagenesis	AGGGGTCTTGAGGGGTTCAGCCCTGGAATTGAAGAGG	CCCCTCAAGACCCCTTCCTGAAGGCCAGAAAGC
**qPCR primers**	**Forward**	**Reverse**
*β-actin*	CAAAAGCCACCCCCACTCCTAAGA	GCCCTGGCTGCCTCAACACCTC
p53	CTCTCCCCCGCAAAAGAAAAA	CGGAACATCTCGAAGCGTTTA
p21	CGAGAACGGTGGAACTTTGAC	CCAGGGCTCAGGTAGACCTT
*Mdm2*	TGTCTGTGTCTACCGAGGGTG	TCCAACGGACTTTAACAACTTCA
*Gadd45a*	CCGAAAGGATGGACACGGTG	TTATCGGGGTCTACGTTGAGC
*Il-1α*	CGAAGACTACAGTTCTGCCATT	GACGTTTCAGAGGTTCTCAGAG
*Il-1β*	GCAACTGTTCCTGAACTCAACT	ATCTTTTGGGGTCCGTCAACT
*Il-6*	TAGTCCTTCCTACCCCAATTTCC	TTGGTCCTTAGCCACTCCTTC
*Ccl2*	TTAAAAACCTGGATCGGAACCAA	GCATTAGCTTCAGATTTACGGGT
*Cxcl1*	CTGGGATTCACCTCAAGAACATC	CAGGGTCAAGGCAAGCCTC
*Serpine1*	TTCAGCCCTTGCTTGCCTC	ACACTTTTACTCCGAAGTCGGT
**Chemicals, enzymes and other reagents**		
RIPA lysis buffer	Thermo Fisher Scientific	Cat #89901
BCA Protein Assay kit	Thermo Fisher Scientific	Cat #23227
DpnI	Thermo Fisher Scientific	Cat #ER1701
Protease inhibitor cocktail	Medchem Express	Cat #HY-K0010
EdU	Sigma-Aldrich	Cat #900584
DAPI	Sigma-Aldrich	Cat #9542
Poly-L-Ornithine	Sigma-Aldrich	Cat #P4957
Heparin	Sigma-Aldrich	Cat #PHR8927
PEI Prime™ linear polyethyleneimine	Sigma-Aldrich	Cat #919012
Proteinase K	Sigma-Aldrich	Cat #SRE0105
Laminin	Roche	Cat #11243217001
Trypsin inhibitor (Ovomucoid)	Worthington	Cat #LS003085
bFGF	Gibco	Cat #100-18B
B27 Supplement	GIbco	Cat #17504044
GlutaMAX Supplement	Gibco	Cat #35050061
DMEM F-12	Gibco	Cat #C11330500BT
Fetal Bovine Serum (FBS)	Gibco	Cat #12483020
RNAiso Plus Trizol	Takara	Cat #9108
PrimeSTAR® HS DNA Polymerase	Takara	Cat #R010A
DMEM	Cytiva	Cat #SH30243.FSS
ECL	Cytiva	Cat #RPN2232
Recombinant Protein G -Sepharose™ 4B	Invitrogen	Cat #101243
ABScript III RT Master Mix for qPCR	ABclonal	Cat #RK20428
2X Universal SYBR Green Fast qPCR Mix	ABclonal	Cat #RK21203
0.25% Trypsin	Welgene	Cat #LS015-04
Penicillin-Streptomycin (100x)	Welgene	Cat #LS202-02
Buffer B	Cell Signaling Technology	Cat #7007S
Micrococcal Nuclease	Cell Signaling Technology	Cat #10011S
**Software**		
GraphPad Prism 8	http://www.graphpad.com/	N/A
Adobe Photoshop 2020	https://www.adobe.com/	N/A
ImageJ	https://www.imagej.net/ij	N/A
Zeiss Zen	http://www.zeiss.com/	N/A
**Other**		
Click-iT™ EdU Cell Proliferation Kit for Imaging, Alexa Fluor 647 dye	Invitrogen	Cat #C10340
Dual-Luciferase® Reporter Assay System 10-Pack	Promega	Cat #E1960
XTT Cell Proliferation Kit	Roche	Cat #11465015001
Beta-galactosidase Assay	Cell Biolabs	Cat #CBA-230
NE-PER™ Nuclear and Cytoplasmic Extraction Reagents	Thermo Fisher Scientific	Cat #78835
Neon™ 10 μL Kit	Thermo Fisher Scientific	Cat #MPK1096
GSE110950 ChIP Seq data	Gene Expression Omnibus	Lee et al (2019)

### Plasmid construction

To construct expression vectors for BBX, p53, and p21, total RNA was isolated from primary NSCs using RNAiso Plus reagent (Takara, Japan). The isolated RNA (2 μg) and oligo(dT) primers (Bionics, South Korea) were used to synthesize first-strand cDNA using a reverse transcription kit (ABclonal, USA) according to the manufacturer’s instructions. The cDNA was amplified by PCR using gene-specific primer pairs. The amplified sequences were cloned into pWPI (Addgene, #12254) for lentiviral transduction of primary NSCs or into pCAGIG (Addgene, #11159) for plasmid-based in utero electroporation. BΔHMG was generated by site-directed mutagenesis using a PCR-based deletion strategy. Briefly, mutagenic primers were designed to span the junction of the deleted region, thereby omitting the target sequence. Whole-plasmid amplification was performed using PrimeSTAR HS DNA Polymerase (Takara, Japan), followed by digestion of the parental wild-type template with DpnI (Thermo Fisher, USA) for 2 h at 37 °C. After DpnI digestion, the amplified products were transformed into *E. coli* DH5α competent cells, and colonies containing the desired constructs were selected. To inhibit p53 function, we used T7-p53DD-pcDNA3 (Addgene, #25989), a plasmid encoding a dominant-negative p53 mutant, originally designated p53DD, and a gift from William Kaelin. In this study, p53DD is referred to as p53DN to indicate its dominant-negative activity. For other BBX deletion mutants (167–888, 334–888, 501–888, 668–888, 1–668, 167–668, 334–668, and 501–668), target sequences were amplified using the corresponding cloning primer pairs. The amplified sequences were cloned into the appropriate vectors as described above. The precise deletion boundaries were verified by Sanger sequencing (Bionics, South Korea). For RNA interference studies, target sequences were cloned into the pLKO.3 G vector (Addgene, #14748). The pLKO.3 G vector was used as a lentiviral vector for in vitro transduction and as a plasmid for in utero electroporation. The pWPI and pCAGIG vectors contain an IRES-GFP cassette, whereas the pLKO.3 G vector expresses GFP from the hPGK promoter. Thus, all gene delivery vectors used in this study allow identification of transduced or electroporated cells by GFP expression. Negative controls received the corresponding backbone vector expressing GFP alone. All cloning primers and shRNA target sequences are listed in the “Reagents and Tools Table”. Newly generated constructs are available from the corresponding author upon reasonable request.

### Lentivirus preparation

Lentivirus was produced by transient co-transfection of lentiviral expression or shRNA plasmids together with the packaging plasmids psPAX2 (Addgene, #12260) and pMD2.G (Addgene, #12259) into Lenti-X™ 293T cells (Takara) using polyethyleneimine (Sigma-Aldrich, USA). Viral supernatants were harvested 72 h after transfection, filtered through a 0.45-μm pore-size filter (Hyundai Micro, South Korea), and concentrated using Lenti-X™ Concentrator (Takara) according to the manufacturer’s instructions. The concentrated viral pellets were resuspended in phosphate-buffered saline (PBS), aliquoted, and stored at −80 °C until use.

### Neurosphere and in vitro differentiation assays

All animal procedures were approved and supervised by the Institutional Animal Care and Use Committee at Sungkyunkwan University (SKKUIACUC2024-01-58-1 and SKKUIACUC2025-04-20-1). All experiments involving animals were conducted in compliance with the relevant institutional and national ethical regulations for the care and use of laboratory animals. Mice were housed in individually ventilated cages on HEPA-filtered ventilated racks under controlled temperature and humidity conditions, with a 12-h light/dark cycle and ad libitum access to food and water. Timed pregnant Hsd:ICR (CD-1) mice (wild-type female *Mus musculus*, outbred strain; Koatech, South Korea; RRID: IMSR_ENV:HSD-030) were used for the preparation of primary NSCs and for in utero electroporation experiments. Mating was initiated at 8 weeks of age. Embryos were considered E0.5 when a vaginal plug was detected in the morning. Primary mouse NSCs were prepared as described previously (Kwon et al, 2023). Unless otherwise specified, all in vitro NSC assays were performed 72 h after lentiviral transduction. For neurosphere assays, 3000 transduced NSCs were plated per well of a 6-well plate at low density (1.5 cells/μl) to minimize the possibility of neurosphere fusion, and neurospheres were quantified after 7 days. To assess self-renewal-associated neurosphere-forming capacity, secondary and tertiary neurosphere assays were performed by dissociating neurospheres from the preceding passage into single-cell suspensions, using the same plating density and culture protocol as for the primary neurosphere assay.

For in vitro differentiation, transduced embryonic NSCs were dissociated into single cells using trypsin and plated on poly-L-ornithine (Sigma-Aldrich)- and laminin (Roche, Switzerland)-coated plates in B27-based NSC medium without FGF. Cells were allowed to differentiate for 48 h or 72 h and were then immunostained for the neuronal marker DCX (Abcam, 1:1000). Neurite morphology was quantified only in GFP⁺DCX⁺ cells by measuring the longest neurite length and the number of primary neurite branches.

### Reverse transcription quantitative PCR (RT-qPCR)

Total RNA was prepared from mouse NSCs using RNAiso Plus reagent (Takara). cDNA was synthesized using ABScript III RT Master Mix (ABclonal, USA) according to the manufacturer’s protocol. Briefly, 500 ng of each RNA sample was reverse transcribed at 55 °C for 15 min and 85 °C for 5 min, followed by cooling at 4 °C. qPCR reactions were performed using 2× Universal SYBR Green Mix (ABclonal) on the Smart Cycler System (Takara). The cycling conditions were 95 °C for 5 min, followed by 40 cycles of 95 °C for 10 s, 60 °C for 15 s, and 72 °C for 20 s. Relative mRNA expression levels of target genes were calculated using the 2^−ΔΔCt^ method and normalized to β-actin. The primers used for qPCR are listed in the “Reagents and Tools Table”.

### XTT-based cell growth assay

Cell viability/proliferation was assessed using the Cell Proliferation Kit II (XTT) (Roche, #11465015001) according to the manufacturer’s instructions. Briefly, 10,000 NSCs transduced with the indicated lentiviral vectors were seeded into each well of a 96-well plate. XTT assays were performed on days 0, 2, and 4 after seeding. At each time point, cells were incubated with a freshly prepared mixture of XTT labeling reagent and electron-coupling reagent for 3 h at 37 °C. The absorbance of each sample was then measured at 490 nm, with a reference wavelength of 650 nm.

### Western blotting

Primary NSCs were lysed using RIPA buffer (Thermo Fisher, USA) supplemented with protease and phosphatase inhibitor cocktails (MedChemExpress, USA). Equal amounts of protein (20–40 μg, depending on the target protein) were resolved by 8–10% (w/v) sodium dodecyl sulfate-polyacrylamide gel electrophoresis (SDS-PAGE) and transferred to polyvinylidene fluoride (PVDF) membranes (Merck, USA). The membranes were blocked with TBST (150 mM NaCl, 10 mM Tris-HCl, 0.1% (v/v) Tween 20, pH 8.0) containing 5% (w/v) skim milk and incubated with the corresponding primary antibodies overnight at 4 °C. The membranes were then incubated with horseradish peroxidase-conjugated secondary antibodies for 2 h at room temperature. Protein bands were visualized using an enhanced chemiluminescence system (Cytiva) and X-ray film (Agfa, Belgium). The antibodies used for western blotting and their working dilutions are listed in the “Reagents and Tools Table”.

### RNA-seq and bioinformatics analysis

For RNA-seq, NSCs isolated from E13.5 embryonic cortices were transduced with shBBX #2 or control lentivirus, cultured for 3 days, and harvested for total RNA extraction using RNAiso Plus reagent. Both lentiviral vectors express GFP as a reporter, and transduction efficiency was assessed by monitoring GFP fluorescence using fluorescence microscopy. GFP expression was observed in nearly all cultured NSCs, indicating highly efficient lentiviral transduction. Library construction and mRNA sequencing were performed by Macrogen Inc. (South Korea). For differentially expressed gene (DEG) analysis, raw read counts were normalized using the relative log expression (RLE) method implemented in the DESeq2 R package. DEGs were defined as genes with an absolute fold change ≥1.5 and Benjamini–Hochberg-adjusted *P* < 0.05, based on the negative binomial Wald test in DESeq2. Gene Ontology (GO) Biological Process enrichment analysis was performed using Metascape (Zhou et al, 2019). Enrichment significance was calculated using the cumulative hypergeometric distribution. Enriched terms were identified using the default Metascape thresholds of *P* < 0.01, minimum count of 3, and enrichment factor >1.5. Redundant enriched terms were clustered based on Cohen’s kappa similarity, and the most statistically significant term from each cluster was displayed. Heatmaps were generated to illustrate the expression profiles of selected DEGs associated with DNA repair, cellular senescence, and cell-cycle regulation. The corresponding gene sets were obtained from MSigDB, including HALLMARK_DNA_REPAIR, FRIDMAN_SENESCENCE_UP, and GOBP_POSITIVE_REGULATION_OF_CELL_CYCLE. Functional annotation and GO enrichment analyses were performed using g:Profiler.

For rescue RNA-seq analysis, NSCs were transduced under a dual-vector design using shControl + shControl, shBBX + shControl, or shBBX + shp21. This design allowed the shBBX viral input to be matched between the shBBX + shControl and shBBX + shp21 conditions. Total RNA was extracted from each sample and subjected to RNA-seq analysis as described above. For comparative analysis of p21-dependent and p21-independent DEGs, genes were considered differentially expressed using a cutoff of absolute fold change ≥1.3 and adjusted *P* < 0.05. BBX-regulated DEGs were first defined by comparing shControl + shControl and shBBX + shControl samples. p21-dependent DEGs were defined as BBX-regulated genes whose expression changes were reversed by p21 co-knockdown in the shBBX + shp21 condition. Specifically, genes upregulated by shBBX and downregulated by shp21, or genes downregulated by shBBX and upregulated by shp21, were classified as p21-dependent DEGs. BBX-regulated genes that were not restored by p21 co-knockdown were classified as p21-independent DEGs. Heatmaps were generated using Z-score-normalized expression values. For ChIP-seq data analysis, the GSE110950 dataset, including SRX3730214, SRX3730215, and SRX3730271, was used for candidate locus-focused inspection of BBX enrichment. ChIP-seq tracks were visualized using Integrative Genomics Viewer (IGV) (Robinson et al, 2011).

### Immunofluorescence

Standard immunofluorescence procedures were performed to detect target proteins or cellular markers in electroporated brains. Briefly, electroporated embryonic brains were fixed with 4% paraformaldehyde, cryoprotected in 30% sucrose, and cryosectioned using a cryostat microtome (Leica, CM1850, Germany). Sections were washed in PBS and blocked for 1 h with PBS containing 2% fetal bovine serum and 0.2% Triton X-100. Samples were then incubated overnight at 4 °C with the corresponding primary antibodies, washed three times with PBS, and incubated for 3 h at room temperature with Alexa Fluor 488- or 555-conjugated secondary antibodies (Invitrogen) diluted in blocking solution. Images were acquired using a confocal microscope (Zeiss, LSM 700). For morphological analysis of migrating neurons, GFP-positive cells in immunostained tissue sections were analyzed using ImageJ. The cell body profile of each GFP-positive cell was manually traced, excluding neurite shafts, to measure area and perimeter. Circularity was calculated using the formula 4π × area/perimeter². The circularity index ranges from 0 to 1, with a value of 1 representing a perfect circle and lower values indicating increasingly elongated or irregular morphologies. The antibodies used for immunostaining and their working dilutions are listed in the “Reagents and Tools Table”.

### In utero electroporation and cell-cycle exit assay

In utero electroporation was performed in E13.5 or E14.5 pregnant mice as described previously (Kim et al, 2018). Briefly, pregnant mice were anesthetized with pentobarbital sodium (Hanlim Pharm, South Korea) before injection. For in utero electroporation, 1–2 μl of DNA solution (2 mg/ml) in PBS was injected into the lateral ventricle of E13.5 or E14.5 embryos. For rescue experiments, target plasmids were mixed at a 1:1 ratio by weight. Using forceps-type electrodes (Nepagene, Japan), DNA was electroporated into embryos with five 50-ms pulses separated by 950-ms intervals. Depending on the analysis, embryos were dissected at E15.5, E17.5, or E18.5. To minimize litter-to-litter variability, embryos from the same pregnant mouse were assigned to multiple plasmid conditions whenever possible. For quantitative analyses, each experimental condition included embryos obtained from at least three independent pregnant mice. For the in vivo cell-cycle exit assay, EdU (100 mg/kg body weight; Sigma-Aldrich) was injected intraperitoneally into pregnant mice 24 h after in utero electroporation. EdU incorporation was visualized 24 h after injection using the Click-iT™ EdU Imaging Kit (Invitrogen) according to the manufacturer’s instructions. Sections were further immunostained using the immunofluorescence protocol described above.

### Luciferase assay

The mouse *Cdkn1a* (p21) promoter-luciferase plasmid was generated by PCR amplification of the −3220 to +300 region relative to the transcription start site of the mouse *Cdkn1a* genomic sequence (Ensembl gene ID: ENSMUSG00000023067) using genomic DNA isolated from NSCs. The amplified fragment was cloned into the pGL3-Basic vector (Promega). The distal p53 response element (p53RE) mutation, in which CATG/TCTTGA/CATG was replaced with GGGG/TCTTGA/GGGG, was introduced by site-directed mutagenesis. The primers used for p21 promoter cloning and p53RE mutagenesis are listed in the “Reagents and Tools Table”. U87MG human glioblastoma cells, obtained from ATCC (USA) and confirmed to be negative for mycoplasma contamination, were co-transfected with the p21 promoter-luciferase reporter plasmid and a Renilla luciferase reporter plasmid, together with the indicated p53 and BBX expression vectors or BBX knockdown vector, using the Neon Transfection System (Thermo Fisher).

### Chromatin immunoprecipitation (ChIP)

Transduced NSCs were cross-linked with 1% formaldehyde for 15 min. Samples were washed three times with cold PBS and homogenized in ChIP lysis buffer containing 1% SDS, 10 mM EDTA, and 50 mM Tris-HCl (pH 8.0), supplemented with protease and phosphatase inhibitor cocktails. After incubation on ice for 10 min, lysates were sonicated on ice using a sonicator (Cole-Parmer, USA) with 20 pulses of 15 s each at 40% amplitude. Lysates were then centrifuged at 20,000×*g* for 10 min at 4 °C, and the supernatants were collected. For each sample, 10 μg of chromatin was incubated overnight at 4 °C with the indicated antibody under rotation: anti-p53 antibody (1:50; Cell Signaling Technology, #2524S), anti-HA antibody (1:50; Cell Signaling Technology, #3724S), or normal IgG control. Immunoprecipitates were isolated by incubation with Protein G Sepharose beads (Invitrogen) and then washed sequentially with low-salt buffer (0.1% SDS, 1% Triton X-100, 2 mM EDTA, 20 mM Tris-HCl [pH 8.0], 150 mM NaCl), high-salt buffer (0.1% SDS, 1% Triton X-100, 2 mM EDTA, 20 mM Tris-HCl [pH 8.0], 500 mM NaCl), LiCl buffer (0.25 M LiCl, 1% NP-40, 1% deoxycholic acid, 1 mM EDTA, 20 mM Tris-HCl [pH 8.0]), and TE buffer (1 mM EDTA, 10 mM Tris-HCl [pH 8.0]). Chromatin was eluted, and cross-linking was reversed by incubation with 0.2 M NaCl at 65 °C for 2 h. Samples were then digested with 10 μg of proteinase K (Sigma-Aldrich), and DNA was purified using a DNA purification kit (SmartGene, South Korea). Enrichment of the genomic *Cdkn1a* (p21) promoter region was determined by qPCR. The qPCR primers used to amplify the genomic p53 motif-containing region in the mouse *Cdkn1a* (p21) promoter were as follows: F, TGTCTGGGCAGCGATCTCT; R, AGCAGAGACTGGAGTCTTAG.

### Co-immunoprecipitation (coIP) assay

For coIP under overexpression conditions, HEK293T cells were transfected with plasmids encoding HA-tagged BBX and Myc-tagged p53 and lysed in immunoprecipitation (IP) lysis buffer containing 50 mM Tris-HCl (pH 8.0), 150 mM NaCl, 1 mM EDTA, and 0.5% Triton X-100, supplemented with protease and phosphatase inhibitor cocktails (MedChemExpress). Cell lysates were incubated overnight at 4 °C with anti-Myc tag antibody (1:50; Santa Cruz Biotechnology, sc-40 X) or anti-HA antibody (1:50; Cell Signaling Technology, #3724S). Recombinant Protein G Sepharose™ 4B beads (Invitrogen) were then added, and immune complexes were captured for 2 h. The beads were washed three times with IP lysis buffer, and bound proteins were eluted by boiling in loading buffer and analyzed by western blotting with anti-Myc tag antibody (1:1000; Santa Cruz Biotechnology, sc-40 X) or anti-HA antibody (1:2000; Cell Signaling Technology, #3724S). For endogenous coIP, E13.5 brain tissues were lysed in the same IP lysis buffer described above. Endogenous BBX was immunoprecipitated using an anti-BBX antibody (1:50; Santa Cruz Biotechnology, sc-377041), and co-immunoprecipitated p53 was detected by western blotting using an anti-p53 antibody (1:300; Cell Signaling Technology, #2524S).

### Chromatin fractionation assay

HEK293T cells transfected with HA-tagged wild-type BBX or BΔHMG were lysed using NE-PER™ Nuclear and Cytoplasmic Extraction Reagents (Thermo Fisher) according to the manufacturer’s instructions. Nuclear soluble fractions were collected, and the remaining insoluble chromatin pellets were resuspended in Buffer B (Cell Signaling Technology, #7007S) and digested with micrococcal nuclease (MNase; Cell Signaling Technology, #10011S) for 10 min at 37 °C. The enzymatic reaction was terminated by adding 10 μl of 1 M EDTA. After centrifugation at 20,000×*g* for 10 min at 4 °C, the supernatants were collected as chromatin-associated fractions. Nuclear soluble and chromatin-associated fractions were mixed with sample loading buffer, boiled, and subjected to western blot analysis. The anti-HA, Lamin A/C, and H3 antibodies used for western blot analysis are listed in the “Reagents and Tools Table”.

### Quantification and statistical analysis

No formal statistical method was used to predetermine sample size. Sample sizes were selected to include at least three independent biological replicates for statistical analysis where applicable, and the exact *n* values are indicated in the corresponding figure legends. For image-based analyses, sections were excluded when tissue integrity, staining quality, or GFP labeling was insufficient to identify the relevant anatomical regions or target cell populations. These criteria were established before quantification. Experimental procedures and data analyses were not performed in a blinded manner. Statistical analyses were performed using Prism 8 (GraphPad). Data from independent groups are presented as the mean ± SEM. Statistical significance was assessed using unpaired two-tailed Student’s *t* tests, one-way ANOVA, or two-way ANOVA, as appropriate.

## Supplementary information

Table EV1

Peer Review File

Data Set EV1

Source data Fig. 1

Source data Fig. 2

Source data Fig. 4

Source data Fig. 5

Source data Fig. 6

EV Figures Source Data

Expanded View Figures

## Data Availability

The RNA-seq datasets generated in this study have been deposited in GEO under accession numbers GSE297615 and GSE337091, corresponding to the BBX knockdown RNA-seq analysis and the p21 rescue RNA-seq analysis, respectively. The datasets are available at https://www.ncbi.nlm.nih.gov/geo/query/acc.cgi?acc=GSE297615 and https://www.ncbi.nlm.nih.gov/geo/query/acc.cgi?acc=GSE337091. The source data of this paper are collected in the following database record: biostudies:S-SCDT-10_1038-S44319-026-00949-4.
