# Supplementary material for: BBX maintains mammalian brain development by repressing p53-mediated p21 expression
Source: EMBO Rep. 2026 Sep 25;27(21):6223–50. doi: 10.1038/s44319-026-00949-4 (PMC13743785; doi:10.1038/s44319-026-00949-4)
Supplement: Supplementary file 1 [file 44319_2026_949_MOESM10_ESM.pdf]

Expanded View Figures

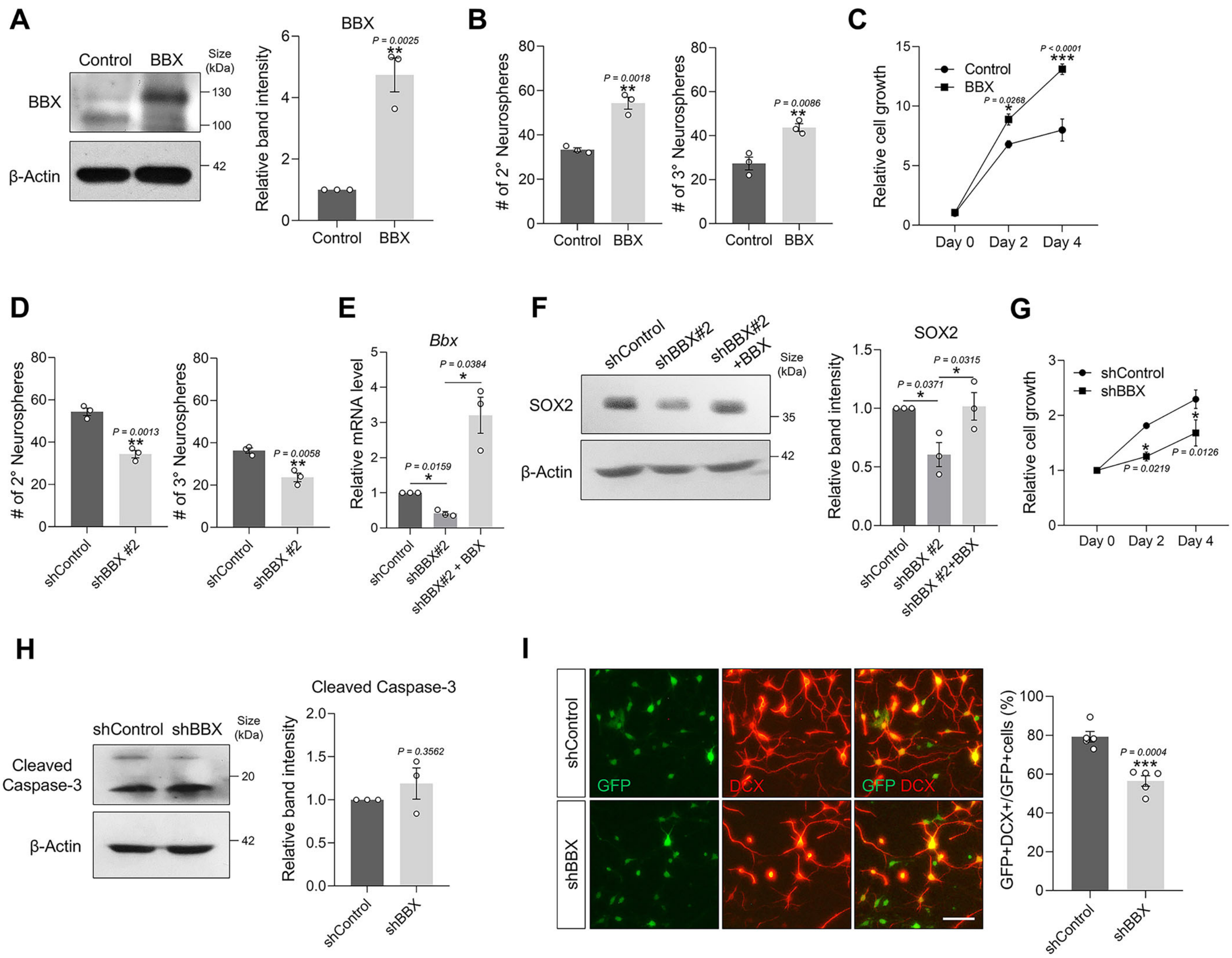

Figure EV1. BBX regulates neurosphere self-renewal and SOX2 expression in NSCs in vitro.

(**A**) Validation of BBX overexpression in NSCs following lentiviral transduction. NSCs were transduced with control or BBX-expressing lentivirus and harvested 2 days later for western blot analysis using an anti-BBX antibody. The graph shows quantification of relative BBX band intensity ($n = 3$). (**B**) Quantification of secondary and tertiary neurospheres formed by NSCs transduced with control or BBX-expressing lentiviral vectors. Primary neurospheres were dissociated and replated at 3000 cells per well to generate secondary neurospheres, and the same procedure was repeated to generate tertiary neurospheres ($n = 3$). (**C**) XTT assay showing the growth of NSCs transduced with lentiviral vectors expressing BBX. Relative cell growth was assessed on days 0, 2, and 4 after seeding ($n = 3$). (**D**) Secondary and tertiary neurosphere assays using NSCs transduced with control or shBBX #2 lentiviral vectors ($n = 3$). Assays were performed as described in (**B**). (**E**) Validation of BBX expression in rescue conditions. NSCs were transduced with shControl, shBBX #2, or shBBX #2 together with BBX-expressing lentivirus, and *Bbx* mRNA levels were measured by qPCR ($n = 3$). (**F**) Western blot analysis showing that BBX co-expression restores SOX2 protein levels reduced by shBBX #2 in NSCs. The graph shows quantification of relative SOX2 band intensity ($n = 3$). (**G**) XTT assay assessing the growth of NSCs transduced with shBBX lentivirus. Relative cell growth was measured on days 0, 2, and 4 after seeding ($n = 3$). (**H**) Western blot analysis of cleaved caspase-3 levels in NSCs transduced with shControl or shBBX lentivirus. Cells were harvested 3 days after transduction and subjected to immunoblotting with an anti-cleaved caspase-3 antibody ($n = 3$). (**I**) GFP (green) and DCX (red) immunostaining in NSCs transduced with shControl or shBBX lentivirus and cultured for 2 days under differentiation conditions. The graph shows the percentage of GFP^+DCX^+ cells among GFP^+ cells. The graph shows immunostaining quantification ($n = 5$). Scale bar, 100 μm. Data information: Data are presented as mean ± SEM from at least three independent biological replicates. Statistical significance was determined by the unpaired Student's *t* test (**A**, **B**, **D**, **H**, **I**), one-way ANOVA (**E**, **F**), and two-way ANOVA (**C**, **G**). *$P < 0.05$, **$P < 0.01$, ***$P < 0.001$. Source data are available online for this figure.

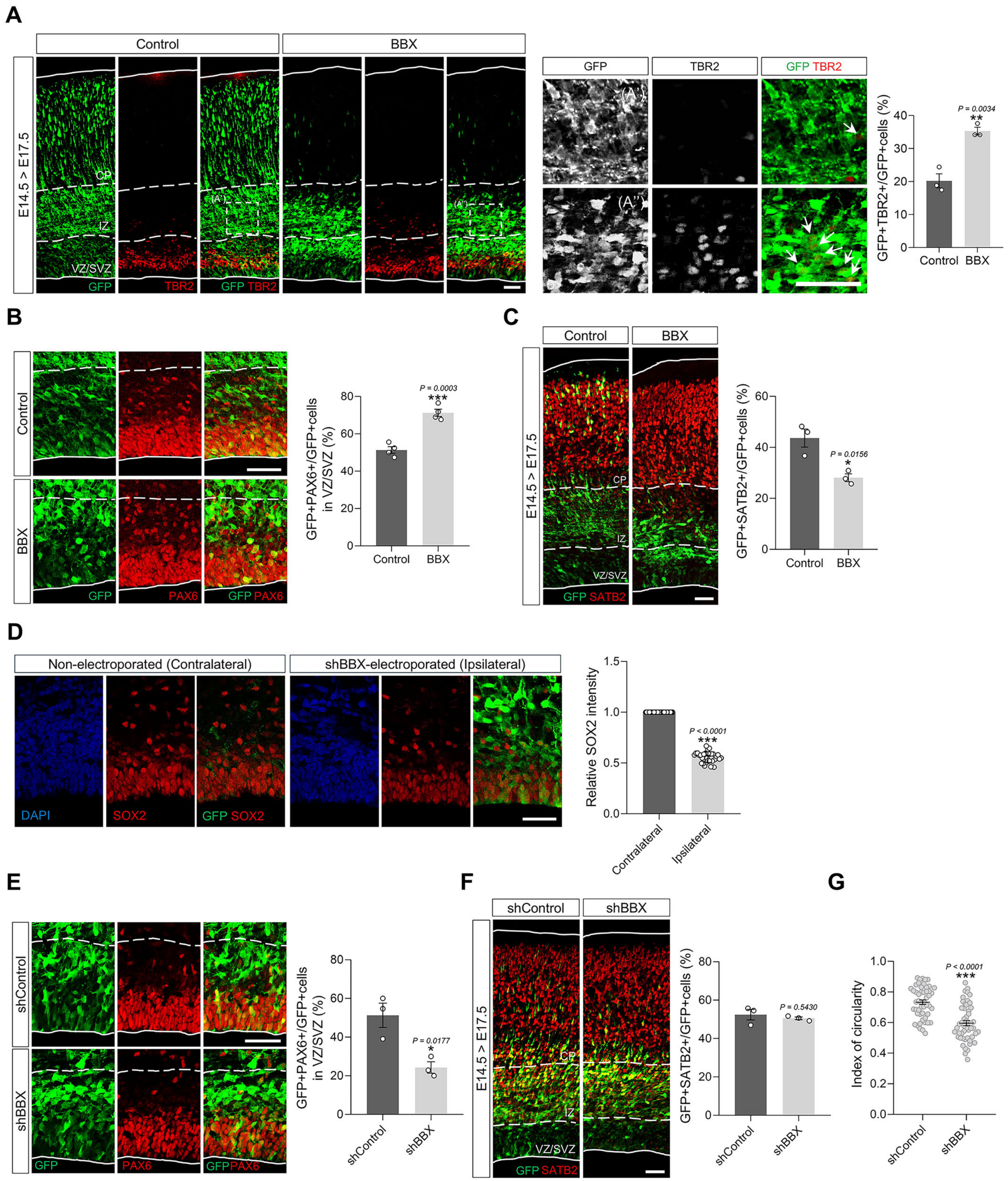
A
Control
BBX
E14.5 > E17.5
CP
IZ
VZ/SVZ
GFP
TBR2
GFP TBR2
(A')
(A'')
GFP+TBR2+/GFP+cells (%)
P = 0.0034
Control BBX
B
Control
BBX
GFP
PAX6
GFP PAX6
GFP+PAX6+/GFP+cells in VZ/SVZ (%)
P = 0.0003
C
GFP SATB2
GFP+SATB2+/GFP+cells (%)
P = 0.0156
D
Non-electroporated (Contralateral)
shBBX-electroporated (Ipsilateral)
DAPI
SOX2
GFP SOX2
Relative SOX2 intensity
P < 0.0001
Contralateral
Ipsilateral
E
shControl
shBBX
P = 0.0177
F
P = 0.5430
G
Index of circularity
P < 0.0001

Figure EV2. BBX modulates cortical progenitor populations and neuronal differentiation marker expression in vivo.

For (**A–G**), embryonic cortices were electroporated with the indicated plasmids at E14.5 and analyzed at E17.5. (**A**) Immunostaining for GFP and TBR2 in brain sections electroporated with a control or BBX expression vector. Magnified views of the boxed areas are shown in (**A**′, **A**′′). Arrows indicate $GFP^{+}TBR2^{+}$ cells. The graph shows the percentage of $GFP^{+}TBR2^{+}$ cells among GFP^{+} cells ($n = 3$). Scale bars, 50 µm. (**B**, **C**) Immunostaining for PAX6 (**B**) and SATB2 (**C**) in embryonic cortices electroporated with control or BBX expression plasmids. Graphs show the frequency of $PAX6^{+}$ or $SATB2^{+}$ cells among GFP^{+} cells ($n = 4$ for (**B**) and $n = 3$ for (**C**)). Scale bars, 50 µm. (**D**) Comparison of SOX2 fluorescence intensity between shBBX-electroporated and non-electroporated VZ regions within the same brain section. Sections were immunostained for GFP (green) and SOX2 (red). The graph shows relative SOX2 fluorescence intensity in the ipsilateral shBBX-electroporated VZ compared with the contralateral non-electroporated VZ ($n = 30$ cells from three biological replicates). Scale bar, 50 µm. (**E**, **F**) Immunostaining for PAX6 (**E**) and SATB2 (**F**) in embryonic cortices electroporated with shControl or shBBX. GFP marks electroporated cells. The graphs show quantification of $PAX6^{+}$ or $SATB2^{+}$ cells among GFP^{+} cells ($n = 3$). Scale bars, 50 µm. (**G**) Analysis of GFP^{+} cell body morphology shown in Fig. 2H. Circularity was calculated using the formula $4\pi \times \text{area}/\text{perimeter}^2$. A value of 1 represents a perfect circle, whereas lower values indicate increasingly elongated or irregular morphologies ($n = 50$ cells from three biological replicates). In (**A–C**, **E**, **F**), solid white lines mark the apical and basal borders of the cortical tissue sections, whereas dashed white lines indicate the boundaries between the VZ/SVZ, IZ, and CP. Data information: Data are presented as mean ± SEM from three biological replicates. Statistical significance was determined by the unpaired Student's *t* test. $^{*}P < 0.05$, $^{**}P < 0.01$, $^{***}P < 0.001$. Source data are available online for this figure.

© The Author(s)

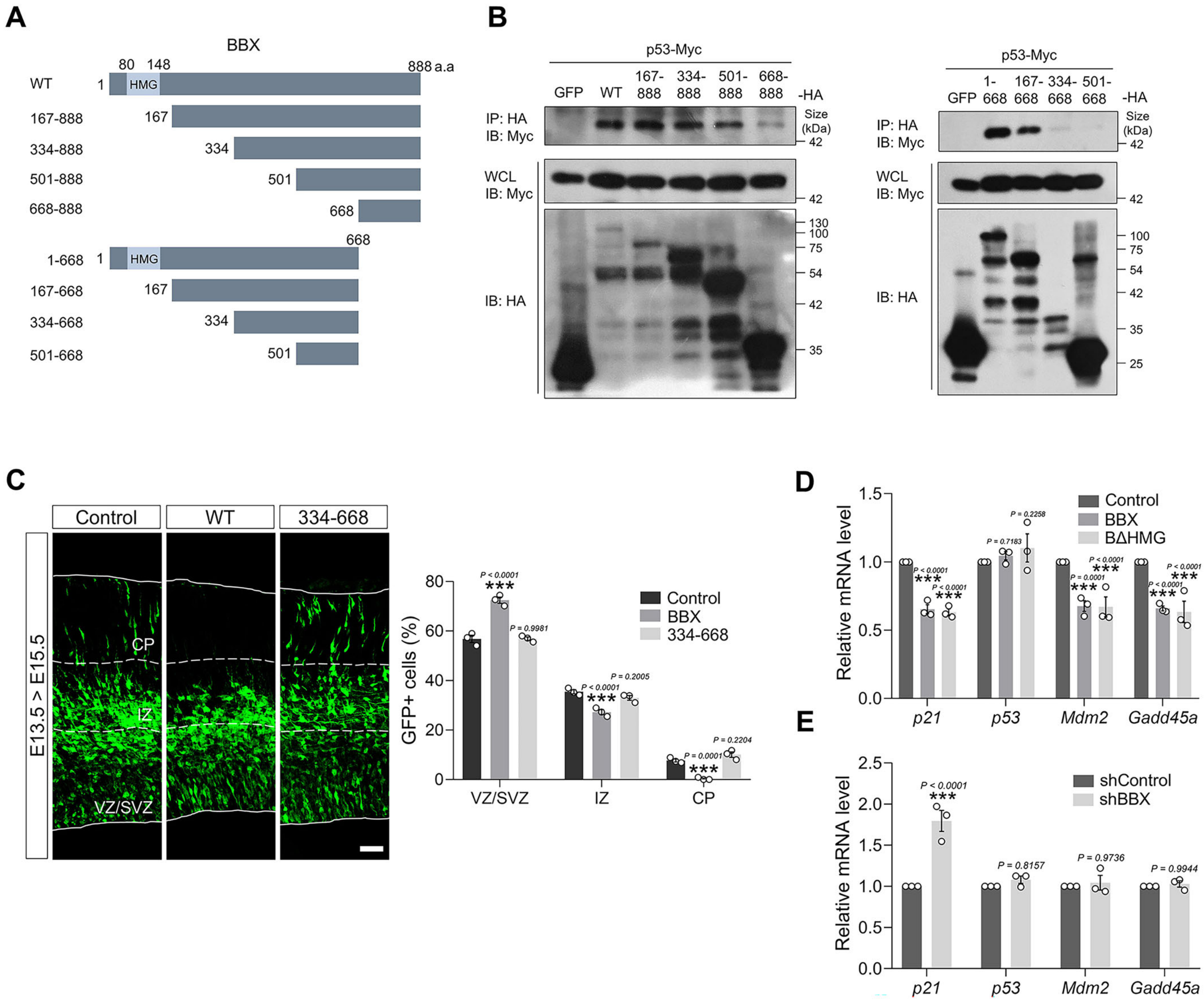

Figure EV3. BBX function depends on its interaction with p53.

(**A**) Schematic representation of wild-type BBX and the indicated BBX truncation mutants. Each mutant is named according to the amino acid residues retained in the construct. HMG box, high mobility group box domain. (**B**) CoIP analysis of p53 binding to BBX truncation mutants. Myc-tagged p53 was co-expressed with HA-tagged wild-type BBX or the indicated truncation mutants. HA immunoprecipitates were immunoblotted with an anti-Myc antibody. WCL, whole-cell lysate. (**C**) Distribution of GFP⁺ cells in embryonic cortices electroporated with control, wild-type BBX, or the BBX 334–668 mutant. Plasmids were introduced by in utero electroporation at E13.5, and brain sections were analyzed at E15.5. Solid white lines mark the apical and basal borders of the cortical tissue sections, whereas dashed white lines indicate the boundaries between the VZ/SVZ, IZ, and CP. The graph shows the distribution of GFP⁺ cells across cortical regions ($n = 3$). Scale bar, 25 μm. (**D**) qPCR analysis of mRNA levels of representative p53 target genes in BBX or BΔHMG -transduced NSCs. Cells were harvested 2 days after transduction, and mRNA levels of p21, p53, MDM2, and GADD45A were measured by qPCR ($n = 3$). (**E**) qPCR analysis of representative p53 target genes in NSCs transduced with shControl or shBBX lentivirus ($n = 3$). Data information: Data are presented as mean ± SEM from three biological replicates. Statistical significance was determined by the two-way ANOVA. ***$P < 0.001$. Source data are available online for this figure.

 © The Author(s)

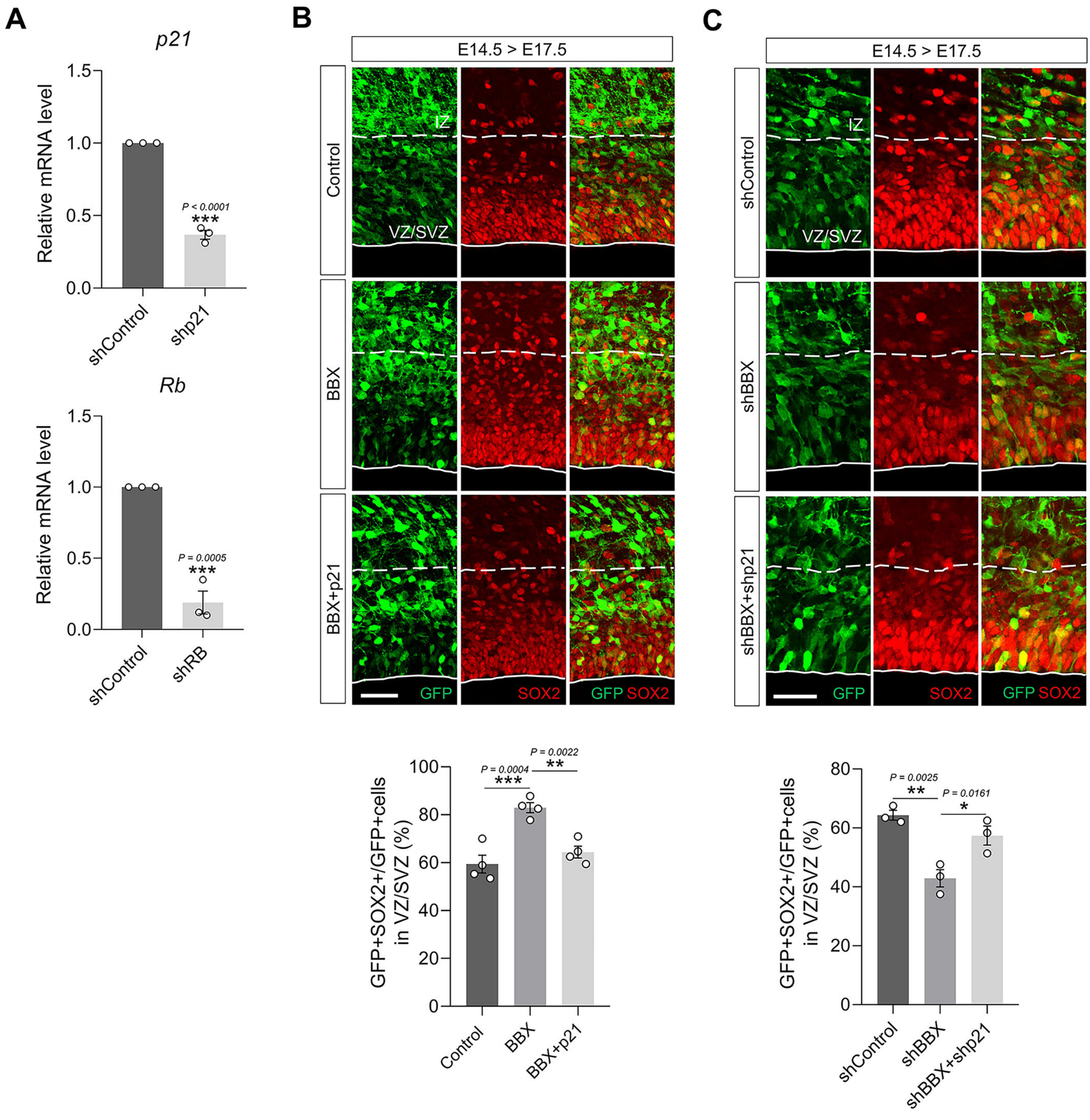

Figure EV4. p21 mediates BBX-dependent regulation of SOX2⁺ progenitor cells in vivo.

(**A**) qPCR analysis of *Cdkn1a* (p21) and *Rb* mRNA levels in NSCs transduced with the indicated lentiviral shRNAs. Cells were harvested 2 days after transduction ($n = 3$). (**B**) GFP and SOX2 immunostaining in embryonic cortices electroporated with control, BBX, or BBX and p21 expression plasmids. Plasmids were introduced by in utero electroporation at E14.5, and brain sections were analyzed at E17.5. The graph shows the percentage of $GFP^{+}SOX2^{+}$ cells among GFP^{+} cells in the VZ/SVZ ($n = 4$). Scale bar, 50 µm. (**C**) SOX2 immunostaining in embryonic cortices electroporated with shControl, shBBX, or shBBX plus shp21 plasmids. Plasmids were introduced by in utero electroporation at E14.5, and brain sections were analyzed at E17.5. The graph shows the percentage of $GFP^{+}SOX2^{+}$ cells among GFP^{+} cells in the VZ/SVZ ($n = 3$). Scale bar, 50 µm. In (**B**, **C**), solid white lines mark the apical border of the cortical tissue sections, whereas dashed white lines indicate the boundaries between the VZ/SVZ and IZ. Data information: Data are presented as mean ± SEM from at least three biological replicates. Statistical significance was determined by the unpaired Student's *t* test (**A**) and one-way ANOVA (**B**, **C**). $^{*}P < 0.05$, $^{**}P < 0.01$, $^{***}P < 0.001$. Source data are available online for this figure.

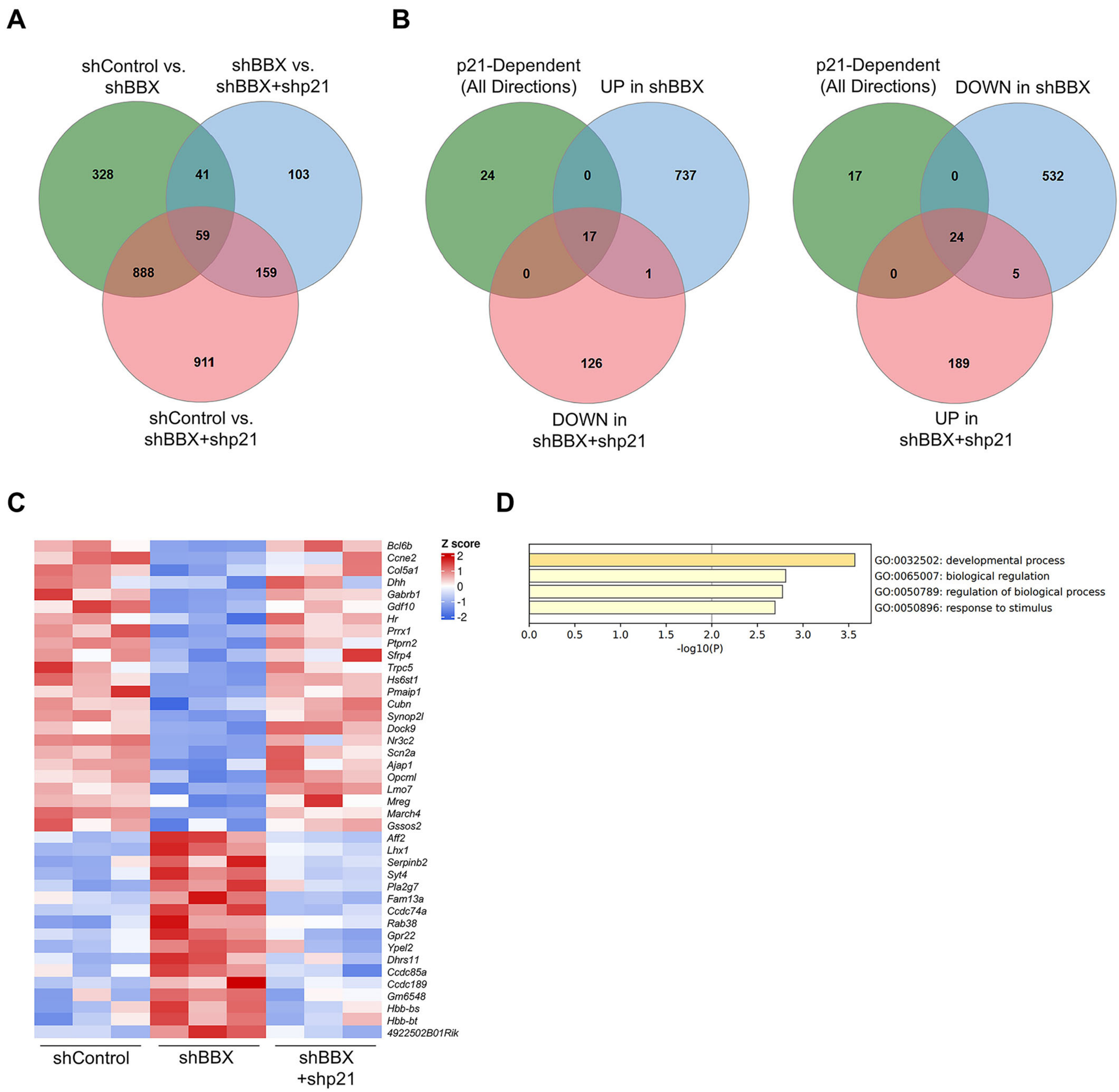

Figure EV5. RNA-seq-based analysis of the p21 dependency of BBX knockdown-regulated genes.

(**A**) Venn diagram showing the overlap of DEGs identified from the indicated pairwise comparisons of RNA-seq samples. DEGs were defined using DESeq2 with the Wald test as those with an absolute fold change ≥1.3 and Benjamini–Hochberg-adjusted $P < 0.05$ ($n = 3$). (**B**) Venn diagrams showing p21-dependent BBX-regulated DEGs. BBX-regulated DEGs whose expression changes were reversed by shp21 co-knockdown were classified as p21-dependent DEGs. The left diagram shows genes upregulated by shBBX and downregulated by shp21 co-knockdown, whereas the right diagram shows genes downregulated by shBBX and upregulated by shp21 co-knockdown. (**C**) Heatmap showing the expression patterns of p21-dependent BBX-regulated DEGs across the rescue RNA-seq conditions. Expression values are shown as Z-scores. (**D**) GO functional enrichment analysis of p21-dependent BBX-regulated DEGs. Enriched GO terms were identified using Metascape based on the cumulative hypergeometric test. Bars indicate −log10(P), and the corresponding GO identifiers and term names are listed on the right.

 © The Author(s)
