# Supplementary material for: BBX maintains mammalian brain development by repressing p53-mediated p21 expression
Source: EMBO Rep. 2026 Sep 25;27(21):6223–50. doi: 10.1038/s44319-026-00949-4 (PMC13743785; doi:10.1038/s44319-026-00949-4)
Supplement: Supplementary file 2 [file 44319_2026_949_MOESM2_ESM.pdf]

EMBO reports

BBX maintains mammalian brain development by repressing p53-mediated p21 expression

Youngik Yoon, Gwang Yeong Kim, Woo Young Lim, Saeah Hwang, Solji Lee, Dongrok Yoon, Sam Pleasure, and Keejung Yoon

Corresponding author(s): Keejung Yoon (keejung@skku.edu)

Review Timeline:

Submission Date:	27th Jul 25
Editorial Decision:	6th Aug 25
Appeal Received:	11th Dec 25
Editorial Decision:	3rd Feb 26
Revision Received:	24th Jul 26
Editorial Decision:	31st Aug 26
Revision Received:	4th Sep 26
Accepted:	11th Sep 26

Editor: Esther Schnapp

Transaction Report:

(Note: With the exception of the correction of typographical or spelling errors that could be a source of ambiguity, letters and reports are not edited. Depending on transfer agreements, referee reports obtained elsewhere may or may not be included in this compilation. Referee reports are anonymous unless the Referee chooses to sign their reports.)

1st Editorial Decision **6th Aug 2025**

Dear Prof. Yoon,

Thank you for the submission of your manuscript to EMBO reports. I have now read and discussed it with my colleagues here, and I am sorry to say that we all agree that it is not well suited for us.

We note that your study reports that depletion of BBX causes defects in newborn neuron migration and cell morphology in mice. You show that BBX overexpression in NSCs derived from E14.5 brain tissues increases neurosphere frequency and SOX2 protein expression, that a mutant lacking the HMG box domain (BΔHMG) has the same effect, that BBX depletion has opposite effects, that BBX overexpressing cells accumulated in the VZ/SVZ in vivo and are depleted from the cortical plate, that BBX depletion reduces neurite outgrowth and changes neuron morphology, that BBX reduces p21 expression and interacts with p53 and decreases p53 binding to the p21 promoter, and that co-depletion of p21 rescues in vivo phenotypes caused by BBX knockdown. We recognize that this will be of interest to researchers in the field.

However, we also note that it remains unclear how p21, a cell cycle regulator, can rescue morphology and cell distribution phenotypes and Sox2 expression. We think that the manuscript is not sufficiently developed and does not provide a sufficient advance for consideration for publication here, and we have therefore decided not to proceed with in-depth review.

Having said that, we hope you consider our not-for-profit open access sister journal, Life Science Alliance (LSA), as a venue for your work (https://www.life-science-alliance.org/about-journal). We have not discussed the paper with the LSA editorial team, but we believe that your work fits the scope of the journal and encourage you to transfer your manuscript to LSA using the link below. Should you transfer, LSA editors will send your manuscript out for peer-review. All files and manuscript details will be automatically transferred, and you will have the opportunity to include specific comments or requests for the LSA editors before finalizing transfer. Please copy our decision letter text in the comments to the LSA editors, so that they are aware that we have committed peer-review on behalf of LSA for this manuscript. Feel free to reach out to the LSA executive editor, Tim Fessenden (t.fessenden@life-science-alliance.org) if you have any questions about the LSA journal or the transfer process.

For EMBO reports, I am sorry that I cannot be more positive this time, and I thank you once more for your interest in our journal.

Yours sincerely,

Esther Schnapp, PhD
Senior Editor
EMBO reports

** As a service to authors, EMBO Press provides authors with the ability to transfer a manuscript that one journal cannot offer to publish to another journal, without the author having to upload the manuscript data again. To transfer your manuscript to another EMBO Press journal using this service, please click on
Link Not Available

Editor

EMBO reports

Dear Editor:

I am pleased to submit a manuscript by Yoon *et al*. entitled “BBX regulates mammalian brain development by repressing p53-mediated p21 expression” for consideration as a Research Article in *EMBO reports*.

In this study, we identify BBX as an important transcriptional regulator during brain development. We show that depletion of BBX, a previously uncharacterized nuclear protein, leads to a significant reduction in neural stem cell stemness, as well as pronounced defects in neuronal migration and cell morphology. Furthermore, we reveal the molecular mechanism underlying these abnormalities, demonstrating that they are caused by increased expression of p53-induced p21.

In our previous submission of this work to EMBO reports (manuscript ID EMBOR-2025-62413V1), the Editor, Dr. Esther Schnapp, raised a concern regarding the mechanistic basis by which knockdown of p21, a cell-cycle regulator, could rescue abnormal neuronal morphology and cell distribution phenotypes observed upon BBX depletion. This unresolved issue was a critical factor in the previous editorial evaluation.

In this resubmission, we have directly addressed this point by investigating an additional, well-established function of p21—its role in cellular senescence. We show that BBX-depleted NSCs undergo senescence, and that the migratory neuron defects observed upon BBX knockdown are attributable to the activation of p21-driven cellular senescence. These new results further reveal that precise suppression of senescence is essential for proper brain development, and that BBX plays a central role in preventing inappropriate activation of senescence. We are grateful for the Editor’s insightful comments, which substantially refined the scope and depth of our study.

We believe our findings will interest researchers across the fields of developmental biology, neuroscience, and stem cell research. Furthermore, as p53 is a protein studied

across various biological fields, the identification of a novel p53 regulator in our study is likely to attract broad interest from researchers in multiple disciplines.

Thank you for considering our manuscript. We look forward to your evaluation.

Sincerely yours,

Keejung Yoon, Ph.D.

Professor

Department of Integrative Biotechnology

Sungkyunkwan University, South Korea

Dear Prof. Yoon,

Thank you for the submission of your manuscript to EMBO reports. We have now received the full set of referee reports that is pasted below.

As you will see, the referees acknowledge that the findings are potentially interesting. However, they also all point out that significant revisions will be required and that the current data are not yet sufficiently strong to support the main conclusions. The referees note a number of technical concerns that would all need to be addressed. The last point by referee 2 will not have to be addressed, i.e. the generation of conditional knockout mouse models, however, if you have such data or are willing to provide these, they will be very welcome, of course.
Please let me know whether or not you will be able to address all other referee comments. We can also discuss the exact revision requirements further, also in a video chat, if you wish.

If we can agree on a set of revisions, I would like to invite you to revise your manuscript with the understanding that the referee concerns must be fully addressed and their suggestions taken on board. Please address all referee concerns in a complete point-by-point response. Acceptance of the manuscript will depend on a positive outcome of a second round of review. It is EMBO reports policy to allow a single round of major revision only and acceptance or rejection of the manuscript will therefore depend on the completeness of your responses included in the next, final version of the manuscript.

We realize that it is difficult to revise to a specific deadline. In the interest of protecting the conceptual advance provided by the work, we recommend a revision within 3 months (6th May 2026). Please discuss the revision progress ahead of this time with the editor if you require more time to complete the revisions.

IMPORTANT NOTE: we perform an initial quality control of all revised manuscripts before re-review. Your manuscript will FAIL this control and the handling will be DELAYED if the following APPLIES:
1) A data availability section providing access to data deposited in public databases is missing. If you have not deposited any data, please add a sentence to the data availability section that explains that.
2) Your manuscript contains statistics and error bars based on n=2. Please use scatter blots in these cases. No statistics should be calculated if n=2.

When submitting your revised manuscript, please review the submission guidelines (https://link.springer.com/journal/44319/submission-guidelines#cms-Revised-submissions) and carefully review the instructions that follow below. Failure to include requested items will delay the evaluation of your revision.

1) a .docx formatted version of the manuscript text (including legends for main figures, EV figures and tables). Please make sure that the changes are highlighted to be clearly visible.

2) individual production quality figure files as .eps, .tif, .jpg (one file per figure). See hhttps://media.springernature.com/original/springer-cms/rest/v1/content/27825798/data/v1 for more info on how to prepare your figures.

3) We replaced Supplementary Information with Expanded View (EV) Figures and Tables that are collapsible/expandable online. A maximum of 5 EV Figures can be typeset. EV Figures should be cited as 'Figure EV1, Figure EV2" etc... in the text and their respective legends should be included in the main text after the legends of regular figures.

- For the figures that you do NOT wish to display as Expanded View figures, they should be bundled together with their legends in a single PDF file called *Appendix*, which should start with a short Table of Content. Appendix figures should be referred to in the main text as: "Appendix Figure S1, Appendix Figure S2" etc. See 'Expanded View' section of the submission guidelines.

- Additional Tables/Datasets should be labeled and referred to as Table EV1, Dataset EV1, etc. Legends have to be provided in a separate tab in case of .xls files. Alternatively, the legend can be supplied as a separate text file (README) and zipped together with the Table/Dataset file.

4) a .docx formatted letter INCLUDING the reviewers' reports and your detailed point-by-point responses to their comments. As part of the EMBO Press transparent editorial process, the point-by-point response is part of the Review Process File (RPF), which will be published alongside your paper.

5) a complete author checklist, which you can download from our author guidelines. Please insert information in the checklist that is also reflected in the manuscript. The completed author checklist will also be part of the RPF.

6) Please note that all corresponding authors are required to supply an ORCID ID for their name upon submission of a revised manuscript (<https://orcid.org/>), which can be linked to your profile in the manuscript submission system.

7) Before submitting your revision, primary datasets produced in this study need to be deposited in an appropriate public database. Please remember to provide a reviewer password if the datasets are not yet public. The accession numbers and database should be listed in a formal "Data Availability" section placed after Methods. Please note that the Data Availability Section is restricted to new primary data that are part of this study. * Note - All links should resolve to a page where the data can be accessed. *

If your study has not produced novel datasets, please mention this fact in the Data Availability Section.

8) At EMBO Press we ask authors to provide source data for the main manuscript figures. You will receive a separate email with instructions for providing source data with your revised manuscript, including how to upload and organize the files.

9) Our journal also encourages inclusion of *data citations in the reference list* to directly cite datasets that were re-used and obtained from public databases. Data citations in the article text are distinct from normal bibliographical citations and should directly link to the database records from which the data can be accessed. In the main text, data citations are formatted as follows: "Data ref: Smith et al, 2001" or "Data ref: NCBI Sequence Read Archive PRJNA342805, 2017". In the Reference list, data citations must be labeled with "[DATASET]". A data reference must provide the database name, accession number/identifiers and a resolvable link to the landing page from which the data can be accessed at the end of the reference.

10) Regarding data quantification, the following points must be specified in each figure legend:

- the name of the statistical test used to generate error bars and P values,

- the number (n) of independent experiments (please specify technical or biological replicates) underlying each data point,

- the nature of the bars and error bars (s.d., s.e.m.),

- If the data are obtained from n Program fragment delivered error ``Can't locate object method "less" via package "than" (perhaps you forgot to load "than"?) at //ejpvfs23/sites23b/embor_www/letters/embor_decision_revise_and_review.txt line 54.' 2, use scatter blots showing the individual data points.

Discussion of statistical methodology can be reported in the materials and methods section, but figure legends should contain a basic description of n, P and the test applied.

- Please also include scale bars in all microscopy images.

11) The journal requires a statement specifying whether or not authors have competing interests (defined as all potential or actual interests that could be perceived to influence the presentation or interpretation of an article). In case of competing interests, this must be specified in your disclosure statement.

12) All Materials and Methods need to be described in the main text using our 'Structured Methods' format, which is required for all research articles. According to this format, the Methods section includes a separate Reagents and Tools Table file (listing key reagents, experimental models, software and relevant equipment and including their sources and relevant identifiers) and a Methods and Protocols section describing the methods using a step-by-step protocol format. The aim is to facilitate adoption of the methodologies across labs. More information on how to adhere to this format as well as a downloadable template (.docx) for the Reagents and Tools Table can be found in our author guidelines.

We would also welcome the submission of cover suggestions, or motifs to be used by our Graphics Illustrator in designing a cover.

As part of the EMBO publication's Transparent Editorial Process, EMBO reports publishes online a Review Process File (RPF) to accompany accepted manuscripts. This File will be published in conjunction with your paper and will include the referee reports, your point-by-point response and all pertinent correspondence relating to the manuscript.

You are able to opt out of this by letting the editorial office know (emboreports@embo.org). If you do opt out, the Review Process File link will point to the following statement: "No Review Process File is available with this article, as the authors have chosen not to make the review process public in this case."

I look forward to seeing a revised form of your manuscript when it is ready.

Kind regards,
Esther

Esther Schnapp, PhD
Senior Editor
EMBO reports

Referee #1:

The manuscript by Yoon et al describes the role of BBX, a nuclear protein with HMG domain during cortical development. The authors propose that the function of BBX during cortical development does not rely on its role as transcription factor but is mediated through its interaction with p53 which leads to suppression of p21expression. Using in vitro neurosphere assays and in utero electroporation (IUE), the authors overexpress and knock-down the expression of BBX and access the progenitor numbers, positioning of targeted cells and their morphology. Using construct that are lacking the HMG domain of BBX, Yoon and colleagues show that the observed effects does not rely on the DNA binding capacity of BBX. Next, using knock down of BBX in neurospheres, the authors identify the differentially regulated genes upon BBX knock-down and by cross referencing this dataset with a publicly available ChIP-seq dataset derived from trophoblast stem cells, the authors identify p21 as a BBX direct target. By analyzing the BBX binding region, they see that BBX binding region overlaps with p53 binding region and using co-IP and luciferase reporter assays, they show that p53 and BBX interact and this interaction which does not depend on the HMG box domain, regulates the p21 promoter activity. Using IUE, the authors manipulate p21 levels to rescue the in vivo phenotypes observed upon overexpression or knock down of BBX. Finally given the role of p21 in senescence, the authors co-knock down BBX and RB, a mediator of p21 induced senescence, and show that the migration related phenotypes are mediated by p21 induced senescence.
The role of BBX has not been assessed during cortical development. Moreover, the authors propose an interesting mechanism by providing convincing set of experiments overall. Yet, the authors need to address some concerns to strengthen their manuscript. In addition, the method part of the manuscript is very poorly written and it makes it very hard to judge the experiments and it will be impossible to be repeated by the scientific community.

1/ Methods: Methods should be written properly as it is very hard to follow what was done in the way they are written now. Moreover, it won't be possible for someone from the scientific community to repeat any of the experiments done in the paper.
In the NSC protocol: Authors refer to Kwon at al.; 2022 (wrongly cited as 2023) for the neurosphere assay. Yet in the paper they cite there is no information regarding the plating density of cells. In higher densities cells are more prone to fuse and this leads to wrong interprations about the neurosphere formation capacity. Their control condition: is it transduced with an empty plasmid or it is not transduced?
There is no information about the constructs and the sequences of shRNAs used. In Figure EV1C, we see GFP expression but it is not possible to understand where does the GFP come from? Do they use a sh construct co-expressing GFP or do they do a co-transduction with a GFP virus.
IUE/ IU transduction: Almost all IUEs were done with 3 embryos. Do all embryos come from one mother? How many independent mothers were electroporated?
In that part also, no information regarding the plasmids used. Is it a co-electroporation with GFP, is it a GFP tagged construct?
Co-IP and ChIP: Information regarding the lysis buffer composition, antibody concantrations used which concentration and sample amounts loaded should be added.

2/ Authors refer to Dixon et al., 2013 for Bbx expression. While in the paper that they cite, the expression pattern of BBX is better visible and studied, the expression pattern in the cortex is not clear. Authors should provide a detailed expression analysis of BBX by co-immunostainings or in minimum they should benefit from public databases to clarify and detail expression of Bbx during cortical development as they claim to have a progenitor specific expression.

3/ Data related to neurospheres: The authors claim BBX is important for maintaining stemness. To be able to do claim using neurospheres, they should do secondary /tertiary neurosphere assays. In this case, it is more appropriate to say BBX increase number of progenitor cells/ their proliferation.
The neurospheres after knock-down of BBX do not seem healthy at all. Neurosphere data should be strengthened by MTT assay (to show cells do not have changes related to survival) and by Ki67, BrdU and neurospehere size measurements (to analyze changes related to cell cycle dynamics).
For the rescue experiment with BBX re-expression, the authors should show resistance of the rescue construct to the shRNAs.

4/ Data related to IUE:
a/ Again, the claim of "increased stemness" does not look appropriate. We can talk about increased # of progenitor cells due to changes in their proliferation/cell cycle dynamics.
b/ In figure 2C, there are a lot of Sox2+ cells in the cortical plate in the control. Normally we don't see progenitor cells in the cortical plate. How does the authors explain this?
Given that in Figure 2E, the authors also see a decreased intensity of Sox2 staining (and do not provide an explanation for this), I think authors should use another NSC marker like Pax6 to quantify those cells.
c/ The authors nicely provide data regarding the induction of senescence phenotype upon BBX knock down in neurospeheres. It

is important to also provide yAH-X and B-gal stainings in vivo after IUE of knock down constructs to confirm that senecesence phenotype is indeed also induced in vivo.

5/ RNA-seq/ChIP-seq data:
For the ChIP-seq from trophoblasts, a supplementary figure showing the number/identity of direct BBX target genes that are deregulated upon BBX knock down should be added together with a list of deregulated and direct target genes.
As the main claim of the paper is that in the context of cortical development, BBX does not act as a transcription factor but a transcriptional regulator through its interaction with p53, among the 400 DEGs upon BBX knock-down, how many were p53 targets? This should be discussed and a list should be provided if available.

6/ Experiments related to HMG box domain and p53 interaction: As one of the main claims of the paper is that BBX function during cortical development is independent of its DNA binding ability, the authors should show that the delta HMG construct does indeed not bind to DNA.
The interaction between BBX and p53 is shown in HEK cells overexpressing the constructs. This data should be supported by showing that the interaction takes place in vivo using cortical samples or in neurospheres cultures either by Co-IP or a PLA assay.
It would be very nice if the authors could identify the region of BBX that interacts with p53 and show that BBX overexpression that cannot bind p53 does not effect cortical development. Yet, given the size of the two proteins, it could be hard to define the region of interaction using deletion constructs.

Referee #2:

This study reveals the essential role of BBX in the maintenance of embryonic neural stem cells (NSCs), cortical neuron migration, and laminar organization, and proposes a relatively novel mechanism whereby BBX suppresses p21 transcription through interaction with p53, thereby preventing premature cell-cycle exit and senescence of NSCs. Overall, the experimental strategy is well designed, combining both in vitro and in vivo approaches, with a clear logical progression from functional characterization to mechanistic analysis and rescue experiments. However, several key scientific issues remain insufficiently clarified or require further strengthening:

1)Insufficient mechanistic explanation for the dispensability of the HMG domain in BBX. Perform ChIP-qPCR analysis in NSCs comparing wild-type BBX and the HMG-deleted mutant (BΔHMG) to determine whether their occupancy at the p21 promoter is similarly dependent on p53 rather than direct DNA binding.

2)Insufficient evidence that p21 is the "primary" or "exclusive" functional downstream target of BBX. Use RNA-seq data to distinguish p21-dependent versus p21-independent differentially expressed genes (DEGs), for example by performing rescue RNA-seq analysis under shBBX + shp21 conditions.

3)Potential confusion caused by apparently similar phenotypes induced by BBX knockdown and BBX overexpression. Include quantitative analyses of cell markers (e.g., NeuN, SATB2, CTIP2) to clearly distinguish the fundamentally different biological outcomes of BBX overexpression versus depletion.

4)Insufficient direct evidence for true senescence in postmitotic neurons. Although the RB rescue experiments support the involvement of senescence, there is currently no direct evidence demonstrating that postmitotic neurons undergo bona fide senescence. Perform co-localization analysis of SA-β-gal activity or p21/γH2AX expression with DCX^+ or $TBR1^+$ neurons.

5)In Figure 1, detection of endogenous BBX protein levels should be included for both BBX overexpression constructs and BBX-targeting shRNAs to validate the efficiency of overexpression and knockdown.

6)In Figure 2, panel 2B lacks quantitative statistical analysis, which should be provided.

7)In Figure 3, clarification is required regarding the RNA-seq sampling strategy: Were samples collected from electroporated cortical regions? What proportion of the tissue consisted of GFP^+ cells? What was the estimated percentage of BBX-depleted cells?

8)In addition, conditional knockout mouse models would provide more robust and reliable results than mosaic knockdown approaches.

Referee #3:

The authors propose that BBX functions as a differentiation suppressor among transcription factors involved in the fate determination of neural stem cells. Through in vitro primary culture experiments involving overexpression and knockdown of BBX, they demonstrate that BBX promotes neural stem cell properties such as neurosphere formation and Sox2 expression. In vivo, using in utero electroporation, they show that BBX is important for maintaining Sox2+ cells in the VZ and also for neurite formation after differentiation. Analyses of RNA-seq and public ChIP-seq data indicated that p21 might be a target, and mechanistically, the authors propose, based on co-IP and reporter assays, that BBX inhibits p53 activity in a DNA-binding-independent manner.
Overall, the manuscript provides many experimental data and propose interesting conclusions. However, several important experimental conditions and protocols are insufficiently described, and the current data do not fully support the conclusions. Therefore, substantial revisions are necessary before the manuscript can be considered for publication.

Major comments
1. There is a lack of detailed description of experimental timelines and procedures, making it difficult to determine whether the authors' conclusions are appropriately supported. The authors should provide detailed methods sufficient for reproducibility. The following specific points should be addressed:
o The experimental timelines and methodologies - for example, the timing of gene introduction and induction of differentiation in Fig. 1G, which is critical for distinguishing effects on NSC fate determination from those on neurite outgrowth - should be explicitly described for each experiment.
o Details regarding plasmid construction for BBX, BΔHMGs, and shRNA are missing (e.g., primers used, backbone plasmids, cloning strategy, or references to commercially available plasmids). The Methods should clarify how GFP is incorporated into samples in each experiment.
o The methods used for DEG analysis should be described in detail, including the software employed, normalization methods, etc. The criteria for selecting the presented GO terms for figures (Fig 3C-D) should also be indicated.
o The catalog number and the provider should be given for all antibodies used, such as Sox2 and γH2AX.
o The Methods should specify the cell type used for luciferase and ChIP assays, specifically in the Results section corresponding to Fig. 4.
o Please explain the nature of the p53DN construct.
o For RT-qPCR, describe the reverse transcription conditions and methodology.
2. As BBX knockdown phenotypes, the authors report: 1) reduced VZ cells, 2) reduced Sox2+ cells, 3) reduced CycD1, 4) increased cell cycle exit, 5) decreased Dcx+, and 6) immature neurites. However, phenotypes 1-4 appear inconsistent with phenotype 5. The authors should discuss explicitly how each cell population at various stages of differentiation is affected.
3. Experiments with the HMG box mutant are critical to support the authors' claims about BBX molecular function. Thus, a precise description of the mutant (is it a point mutation, an HMG box deletion, and if so, which residues?) is essential. It is also necessary to demonstrate that this mutant truly lacks DNA-binding activity.
4. As also noted above, the experiments in Fig. EV1C and Fig. 1G need to distinguish clearly between suppression of differentiation versus suppression of neurite outgrowth. In Fig. 1G, it appears that NSCs as well as neurons are present in the images; thus, neuronal neurites should be quantified using neuronal markers. Furthermore, when quantifying dendritic branches, how are dendrites distinguished from axons?
5. Regarding Fig. 3D: among the genes included, which are DEGs? There are more genes involved in cell proliferation/inhibition, DNA damage response, and senescence-what criteria were used to select genes shown in this figure? What does "a clear correlation with BBX depletion" mean? Also, showing Cdkn1a twice in the same figure is misleading.
6. The Results section should include interpretation of findings rather than only phenotypic descriptions.
7. For Fig. 4E, please indicate the p-value between control and knockdown for the p21p-mt sample. If it is significantly increased, this would suggest that the effect of BBX is only partial, so this possibility should be discussed thoroughly.
8. The phrase "the physiological integrity of immature neurons" is unclear-what aspect of "integrity" is intended here?

Minor comments
9. In the Abstract, "BBX suppression of p21" is ambiguous; it should be "suppression of p21 by BBX".
10. The authors state that BBX is expressed only in the VZ; have they confirmed this themselves? Figs. 1 and 2 of Dixon, 2013 do not make it clear whether BBX is specifically expressed in the neocortex VZ.
11. The descriptors "round" and "irregular" in Fig. EV2B are too subjective; more precise definitions and clear quantification criteria should be provided.
12. Was the GO analysis in Fig. 3C performed on up- or down-regulated genes? What does "shBBX / shControl" refer to in the figure?
13. The statement "Given that BBX knockdown reduces cyclin D1 expression both in vitro and in vivo,"-is this previously known or shown in the RNA-seq results? If it is known, a citation is required.
14. Gene names should be italicized throughout, e.g., in Figs. 3D and 3E.
15. In Figs. 3E and 4A, gene structures such as exons, introns, and transcriptional orientation should be annotated. In Fig. 3E, a diagram of a known BBX-binding locus as positive control would be helpful. Were the "proliferation genes" in Fig. 3D examined for BBX binding?
16. U87MG, used in the reporter assay, is a human cell line; can mouse knockdown vectors function effectively in this context?
17. The manuscript suggests that BBX acts as a p53 cofactor and that overexpression results indicate MDM2 and GADD45A may also be targets. Is ChIP evidence for BBX binding at the MDM2 and GADD45A loci available?

18. The efficiency of the knockdown vector for p21 should be shown.
19. In the experiments with p53DN, p21, and RB knockdown, there is a marked reduction of cells accumulating in the IZ. What is the relationship between these IZ-accumulating cells, cell cycle exit, and senescence?
20. Since knockdown experiments were performed in vivo, it would be important to examine γH2AX staining in these samples to support the conclusions.
21. In Fig. 6A-B, please show SOX2 immunostaining on tissue sections rather than relying on Western blotting alone, since this would provide more direct evidence.

Response to Referees' Comments

Referee #1:

The manuscript by Yoon et al describes the role of BBX, a nuclear protein with HMG domain during cortical development. The authors propose that the function of BBX during cortical development does not rely on its role as transcription factor but is mediated through its interaction with p53 which leads to suppression of p21expression. Using in vitro neurosphere assays and in utero electroporation (IUE), the authors overexpress and knock-down the expression of BBX and access the progenitor numbers, positioning of targeted cells and their morphology. Using construct that are lacking the HMG domain of BBX, Yoon and colleagues show that the observed effects does not rely on the DNA binding capacity of BBX. Next, using knock down of BBX in neurospheres, the authors identify the differentially regulated genes upon BBX knock-down and by cross referencing this dataset with a publicly available ChIP-seq dataset derived from trophoblast stem cells, the authors identify p21 as a BBX direct target. By analyzing the BBX binding region, they see that BBX binding region overlaps with p53 binding region and using co-IP and luciferase reporter assays, they show that p53 and BBX interact and this interaction which does not depend on the HMG box domain, regulates the p21 promoter activity. Using IUE, the authors manipulate p21 levels to rescue the in vivo phenotypes observed upon overexpression or knock down of BBX. Finally given the role of p21 in senescence, the authors co-knock down BBX and RB, a mediator of p21 induced senescence, and show that the migration related phenotypes are mediated by p21 induced senescence.

The role of BBX has not been assessed during cortical development. Moreover, the authors propose an interesting mechanism by providing convincing set of experiments overall. Yet,

the authors need to address some concerns to strengthen their manuscript. In addition, the method part of the manuscript is very poorly written and it makes it very hard to judge the experiments and it will be impossible to be repeated by the scientific community.

> Before providing our point-by-point responses, we sincerely thank the referee for the constructive comments and helpful suggestions. The referee's comments helped us improve the manuscript by expanding the Methods section, clarifying the experimental basis for our conclusions, replacing broad or potentially ambiguous terms such as "stemness" with more data-driven descriptions, and adding analyses that strengthen the in vivo relevance of the study. We are grateful for these insightful suggestions, which have significantly improved the clarity and rigor of the revised manuscript. Our point-by-point responses are provided below.

1/ Methods: Methods should be written properly as it is very hard to follow what was done in the way they are written now. Moreover, it won't be possible for someone from the scientific community to repeat any of the experiments done in the paper.

In the NSC protocol: Authors refer to Kwon at al.; 2022 (wrongly cited as 2023) for the neurosphere assay. Yet in the paper they cite there is no information regarding the plating density of cells. In higher densities cells are more prone to fuse and this leads to wrong interprations about the neurosphere formation capacity.

> We apologize that the Methods section in the original manuscript did not provide sufficient experimental detail. In the revised manuscript, we have substantially expanded the Methods section to describe the experimental procedures more clearly.

> We appreciate the referee's attention to detail regarding the citation year. We would like to clarify that while the article by Kwon et al. was first published online in 2022 (Epub ahead of

print), it was officially published with a volume and page/article numbers in 2023. Therefore, we initially cited it as 2023, as the publisher itself explicitly indicates "EMBO Rep (2023) 24: e55313" on the first page of the article.

> As commented, the cited Kwon et al. paper did not include the plating density used for the neurosphere assay, and we agree that plating density is an important parameter because excessive cell density can increase neurosphere fusion and confound the interpretation of neurosphere-forming capacity. In our neurosphere assay, NSCs were seeded at 3,000 cells per well in 6-well plates. This relatively low plating density was used to minimize neurosphere fusion and to better assess neurosphere-forming capacity. We have now clarified the plating density in the revised Methods section.

Their control condition: is it transduced with an empty plasmid or it is not transduced?

> As commented, the vector structures and control conditions were not clearly described in the original manuscript. In the revised manuscript, we have summarized the plasmids used for each experimental purpose, including pWPI, pCAGIG, and pLKO.3G, in the “Plasmid construction” section of the Methods. All gene delivery vectors used in this study contain a GFP expression cassette. In all experiments, the control condition was transduced or electroporated with the corresponding backbone vector, rather than left untransduced. Thus, control cells expressed GFP alone from the same vector backbone used for the experimental constructs. We have also clarified this point in the revised Results and Methods sections.

There is no information about the constructs and the sequences of shRNAs used.

> We thank the referee for pointing out this omission. Information on the constructs and

shRNA sequences was not sufficiently provided in the original manuscript. In addition, the Reagents and Tools Table, which should have accompanied the original submission, was inadvertently omitted from the submitted files. In the revised manuscript, we have added a detailed description of plasmid construction to the "Plasmid construction" section of the Methods, and we have provided the primer sequences used for gene amplification and the shRNA sequences used in this study in the Reagents and Tools Table.

In Figure EV1C, we see GFP expression but it is not possible to understand where does the GFP come from? Do they use a sh construct co-expressing GFP or do they do a co-transduction with a GFP virus.

> As described above, the lentiviral shRNA vector used in this study, pLKO.3G, contains both the shRNA expression cassette and a GFP reporter cassette. Thus, GFP expression in Fig. EV1C originated from the same shRNA vector, and no separate GFP virus was used. Negative control cells were transduced with the empty pLKO.3G backbone and expressed GFP alone. We have clarified this point in the revised Results and Methods sections.

IUE/ IU transduction: Almost all IUEs were done with 3 embryos. Do all embryos come from one mother? How many independent mothers were electroporated?

> As commented, the number of independent pregnant mice used for the IUE experiments was not clearly described in the original manuscript. To minimize litter-to-litter variability, embryos from the same pregnant mouse were assigned to multiple plasmid conditions whenever possible, rather than assigning each pregnant mouse to a single plasmid condition. For all quantified IUE experiments, each experimental condition included embryos obtained from at least three independent pregnant mice. Thus, the quantified

embryos for any given condition were not derived from a single litter. We have clarified this information in the revised Methods section.

In that part also, no information regarding the plasmids used. Is it a co-electroporation with GFP, is it a GFP tagged construct?

> As described above, the overexpression vector pCAGIG and knockdown vector pLKO.3G used for in utero electroporation both contain a GFP reporter cassette. Thus, GFP was expressed from the same vector backbone carrying the inserted coding sequence or shRNA cassette, and was not delivered by co-electroporation of a separate GFP plasmid. The constructs were also not GFP-tagged. Negative controls were electroporated with the corresponding empty backbone vectors, which express GFP alone and therefore allow identification of electroporated cells. We have clarified this point in the revised Results and Methods sections.

Co-IP and ChIP: Information regarding the lysis buffer composition, antibody concantrations used which concentration and sample amounts loaded should be added.

> As commented, the original Methods section did not provide sufficient experimental details for the Co-IP and ChIP assays. In the revised Methods section, we have added the composition of the lysis and wash buffers, the amount of chromatin used for immunoprecipitation, and the antibody dilutions or amounts used for each assay.

2/ Authors refer to Dixon et al., 2013 for Bbx expression. While in the paper that they cite, the expression pattern of BBX is better visible and studied, the expression pattern in the cortex is not clear. Authors should provide a detailed expression analysis of BBX by co-

immunostainings or in minimum they should benefit from public databases to clarify and detail expression of Bbx during cortical development as they claim to have a progenitor specific expression.

> We thank the referee for raising this important point regarding the expression pattern of BBX in the developing brain. To better establish the rationale for this study, we have revised the beginning of the Results section to include an in situ hybridization image of *Bbx* obtained from the GenePaint database (http://www.genepaint.org) as Fig. 1A. This image shows prominent *Bbx* expression in the germinal zone of the developing cortex, including the VZ/SVZ.

3/ Data related to neurospheres: The authors claim BBX is important for maintaining stemness. To be able to do claim using neurospheres, they should do secondary /tertiary neurosphere assays. In this case, it is more appropriate to say BBX increase number of progenitor cells/ their proliferation.

> We agree with the referee that primary neurosphere formation alone is not sufficient to make a definitive claim regarding stemness. In the revised manuscript, we have therefore avoided overstatement and revised the relevant text to more precisely describe the experimental findings in terms of neurosphere-forming capacity and stem/progenitor characteristics. To address the referee's comment, we performed secondary and tertiary neurosphere assays under both BBX overexpression and knockdown conditions. BBX overexpression increased primary, secondary, and tertiary neurosphere formation, whereas BBX knockdown reduced neurosphere formation across passages (Fig. 1B and EV1B; Fig. 1I and EV1D). Together with the corresponding changes in SOX2 expression (Fig. 1C and J), these results support the conclusion that BBX enhances self-renewal-associated neurosphere-forming capacity and helps maintain stem/progenitor characteristics in NSCs.

We have added these new data to Fig. EV1 and revised the Results section accordingly.

The neurospheres after knock-down of BBX do not seem healthy at all. Neurosphere data should be strengthened by MTT assay (to show cells do not have changes related to survival) and by Ki67, BrdU and neurospehere size measurements (to analyze changes related to cell cycle dynamics).

> Following the referee's suggestion, we performed an XTT assay, which is based on the same principle as the MTT assay, and added the results to Fig. EV1C and EV1G. BBX overexpression increased viable/metabolically active cell growth, whereas BBX knockdown reduced it. In addition, BBX knockdown reduced neurosphere size (Fig. 1I), consistent with impaired proliferative expansion. To further assess cell-cycle progression under proliferative conditions, we also performed an EdU incorporation assay and found that BBX knockdown reduced EdU incorporation in NSCs (Fig. 1K).

> To address the referee's concern regarding cell survival, we examined cleaved caspase-3 levels and found no significant increase upon BBX knockdown (Fig. EV1H). This result suggests that the reduction in neurosphere formation and XTT signal is not primarily due to increased apoptotic cell death, but is more consistent with impaired NSC growth and proliferative expansion.

> We agree with the referee's observation that BBX-depleted neurospheres appeared morphologically compromised. In light of the additional data presented later in the revised manuscript, this phenotype may reflect, at least in part, senescence-associated changes induced by BBX knockdown through p21 upregulation. This observation therefore prompted us to examine whether senescence-related genes were altered in the RNA-seq analysis, as now shown in Fig. 3D. However, because the molecular evidence for p21 induction and

senescence-associated changes is introduced in subsequent Results sections, we avoided discussing senescence at this earlier point and instead first presented the direct growth- and cell-cycle-related analyses.

For the rescue experiment with BBX re-expression, the authors should show resistance of the rescue construct to the shRNAs.

> To address this point, we measured BBX mRNA levels in NSCs co-transduced with shBBX and the BBX overexpression lentiviral vector (Fig. EV1E). The BBX coding sequence used for re-expression was the wild-type sequence and was not specifically engineered to be shRNA-resistant. Nevertheless, BBX mRNA expression in co-transduced cells was restored to a level higher than that in control cells, indicating that sufficient BBX re-expression was achieved under the rescue condition. We have clarified this point in the revised Results section.

4/ Data related to IUE:

a/ Again, the claim of "increased stemness" does not look appropriate. We can talk about increased # of progenitor cells due to changes in their proliferation/cell cycle dynamics.

> We agree with the referee that the phrase “enhanced neural stemness” in the original manuscript was too broad in this context. The SOX2 and TBR2 immunostaining data more directly indicate an increase in progenitor-marker-positive cells in the VZ/SVZ, rather than providing definitive evidence for increased stemness. In this revision, we performed additional marker analyses and found that BBX overexpression increased the proportion of PAX6$^+$ cells within the VZ/SVZ, while reducing the proportion of SATB2$^+$ cells among BBX-delivered cells (Fig. EV2B and EV2C). Based on these data, together with the original

SOX2 and TBR2 results, we have revised the text to avoid overstatement and now describe the phenotype as follows: “the increased VZ/SVZ distribution caused by BBX overexpression is associated with expansion or retention of progenitor-marker-positive populations and reduced progression toward a SATB2^{+} upper-layer neuronal fate at this developmental stage.”

> We also revised the related statement on the in vitro experiments. For example, in describing the stronger effect of shBBX #2 compared with shBBX #1, “stronger impairment of neural stemness” was replaced with “stronger impairment of neurosphere-forming capacity and SOX2/Cyclin D1 expression” to avoid overstatement and more accurately reflect the experimental data.

b/ In figure 2C, there are a lot of Sox2+ cells in the cortical plate in the control. Normally we don't see progenitor cells in the cortical plate. How does the authors explain this?

> We agree with the referee that neural progenitor cells are not normally expected to be abundant in the cortical plate, and therefore SOX2-positive signals observed in this region should be interpreted with caution. We do not interpret SOX2 immunoreactivity in the CP as evidence for ectopic localization of neural progenitor cells. However, similar SOX2 immunostaining patterns, with SOX2-positive signals extending beyond the VZ/SVZ into more superficial cortical regions, have been observed in multiple previous studies using untreated or wild-type embryonic brains; representative examples from published and preprint studies are shown below (Hirai et al., 2012; Hagey et al., 2020; Paez-Beltran et al., 2026). Thus, this staining pattern is not unique to our samples and may reflect antibody-dependent staining properties or background immunoreactivity under certain experimental conditions.

> To address this concern through an independent approach, and in response to the referee's subsequent request for PAX6 staining, we performed additional PAX6 immunostaining as a complementary progenitor marker and included the results in the revised manuscript (Fig. EV2B and EV2E)

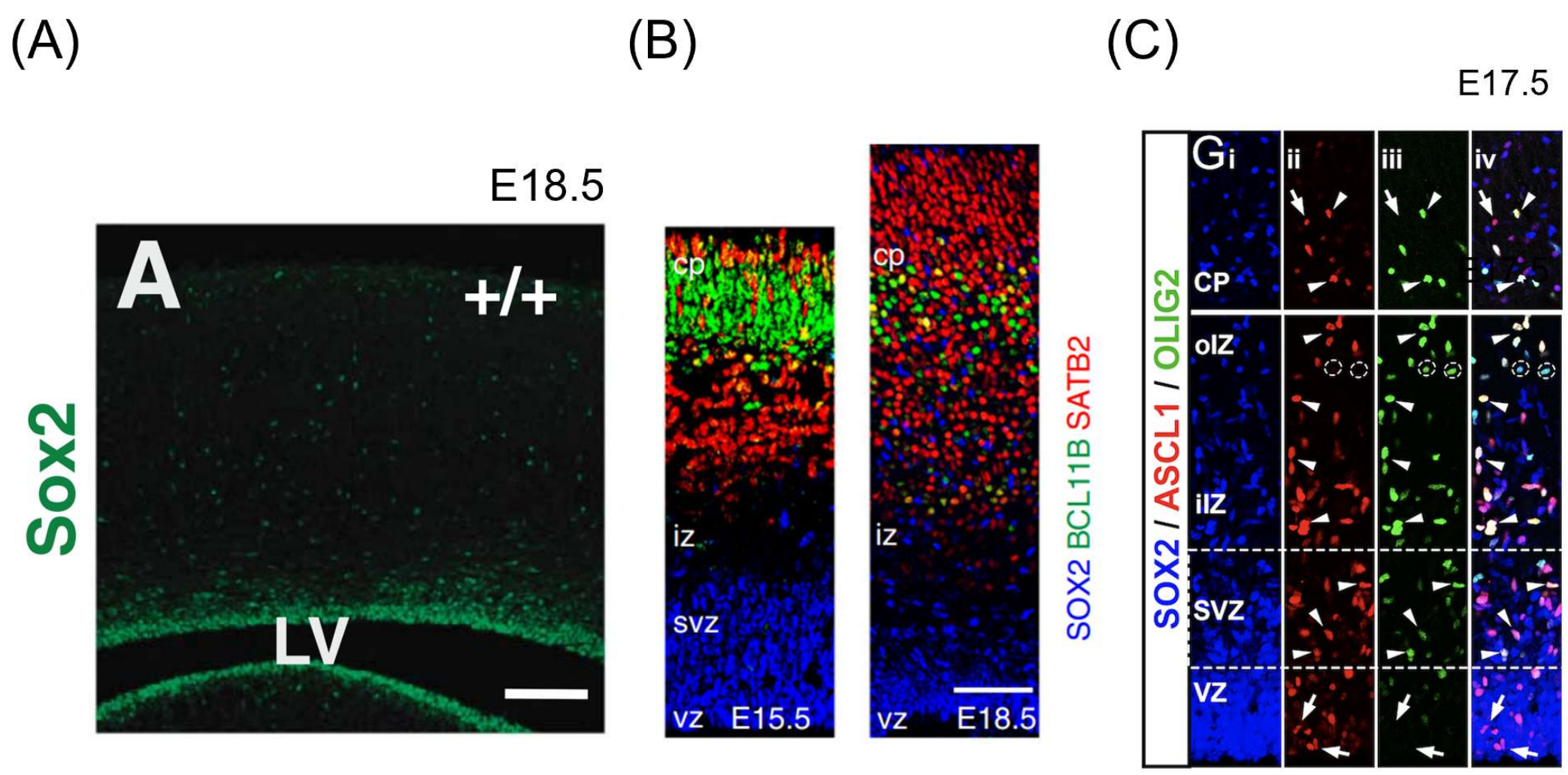

Representative examples of SOX2 immunostaining patterns reported in previous studies using wild-type embryonic brains, showing SOX2-positive signals outside the VZ/SVZ, including more superficial cortical regions. Sources: (A) Hirai et al., 2012, EMBO J 31: 1190–1202, Fig. 1A; (B) Hagey et al., 2020, Nat Commun 11: 2898, Fig. 1a; (C) Paez-Beltran et al., 2026, bioRxiv, DOI: 10.64898/2026.02.02.703158, Fig. 1G.

Given that in Figure 2E, the authors also see a decreased intensity of Sox2 staining (and do not provide an explanation for this), I think authors should use another NSC marker like Pax6 to quantify those cells.

> We appreciate the referee's careful observation. We agree that SOX2 staining intensity appeared consistently weaker in shBBX-electroporated regions. Based on our in vitro data showing reduced SOX2 protein levels upon BBX knockdown, we interpreted this in vivo phenotype as reflecting not only a reduction in the number of $SOX2^+$ cells, but also a

decrease in SOX2 protein levels at the single-cell level.

> To examine this more directly, in the revised manuscript we quantified SOX2 fluorescence intensity in shBBX-electroporated regions and compared it with the contralateral non-electroporated VZ region within the same brain section. This within-section comparison allowed us to minimize potential variation arising from differences in staining or imaging conditions between sections. This analysis confirmed that SOX2 fluorescence intensity was reduced in shBBX-electroporated cells, and we have included these results in Fig. EV2D and the revised Results section.

> Following the referee's suggestion, we also performed PAX6 immunostaining using both BBX overexpression and BBX knockdown samples and added these results to Fig. EV2B and EV2E in the revised manuscript. Consistent with the SOX2 staining results, BBX knockdown reduced the proportion of PAX6^{+} cells among GFP^{+} cells in the VZ/SVZ.

c/ The authors nicely provide data regarding the induction of senescence phenotype upon BBX knock down in neurospeheres. It is important to also provide yAH-X and B-gal stainings in vivo after IUE of knock down constructs to confirm that senecesence phenotype is indeed also induced in vivo.

> We agree with the referee that it is important to examine whether a senescence-associated phenotype is also induced in vivo. To address this point, we performed γH2AX staining after in utero electroporation of the shBBX construct and added the results to Fig. 5G and H. In the original manuscript, we showed that the abnormal cell clusters accumulating in the IZ of shBBX-electroporated cortices were composed largely of DCX^{+} cells. In the revised manuscript, we now show that γH2AX signals were detected at a markedly higher frequency in these IZ cell clusters compared with control samples (Fig.

5G). In addition, γH2AX-positive signals were also observed in DCX^+ migratory neurons located in the CP region (Fig. 5H). These findings provide in vivo support for the induction of a senescence-associated DNA damage response in shBBX-electroporated cortical cells.

> We also attempted β-galactosidase colorimetric staining and observed increased signals in shBBX-electroporated tissues, as shown below. However, when this staining was combined with GFP immunostaining to identify electroporated cells, tissue morphology and integrity were not sufficiently preserved for inclusion in the revised manuscript. Although we were therefore unable to include the β-galactosidase staining data in the revised manuscript, the added γH2AX staining provides in vivo support for the induction of a senescence-associated phenotype upon BBX knockdown.

Figure for referee with unpublished data and its description has been removed upon request by the authors.

5/ RNA-seq/ChIP-seq data:

For the ChIP-seq from trophoblasts, a supplementary figure showing the number/identity of direct BBX target genes that are deregulated upon BBX knock down should be added together with a list of deregulated and direct target genes.

> We agree with the referee that a genome-wide analysis integrating BBX ChIP-seq peaks with genes deregulated upon BBX knockdown would be informative. We initially considered including this type of analysis in the original manuscript. However, the publicly available

BBX ChIP-seq dataset generated under proliferative conditions in trophoblast stem cells contains only two GEO samples, GSM3019272 and GSM3019273. As shown in the representative comparison below, GSM3019273 showed substantially weaker and less consistent BBX ChIP-seq signals than GSM3019272 across the examined loci. Because only one sample, GSM3019272, was suitable for reliable peak-based analysis, we did not consider it appropriate to generate a genome-wide list of direct BBX target genes or to present a quantitative overlap analysis with BBX knockdown-regulated DEGs based on this dataset alone.

> Instead, we took a more focused and conservative approach by examining whether selected genes identified from our RNA-seq analysis were associated with BBX occupancy in the relatively higher-quality ChIP-seq dataset, GSM3019272. Using this approach, we found that *Cdkn1a* (p21) showed BBX occupancy at its promoter. Notably, *Cdkn1a* (p21) was one of the few loci at which BBX enrichment was observed in both proliferative-condition samples, GSM3019272 and GSM3019273, despite the overall poor signal quality of GSM3019273. These observations support the relevance of *Cdkn1a* (p21) as a candidate BBX-associated target. We have clarified this limitation and the rationale for our focused analysis in the revised Results section.

Column 1 = GSM3019272; Column 2 = GSM3019273

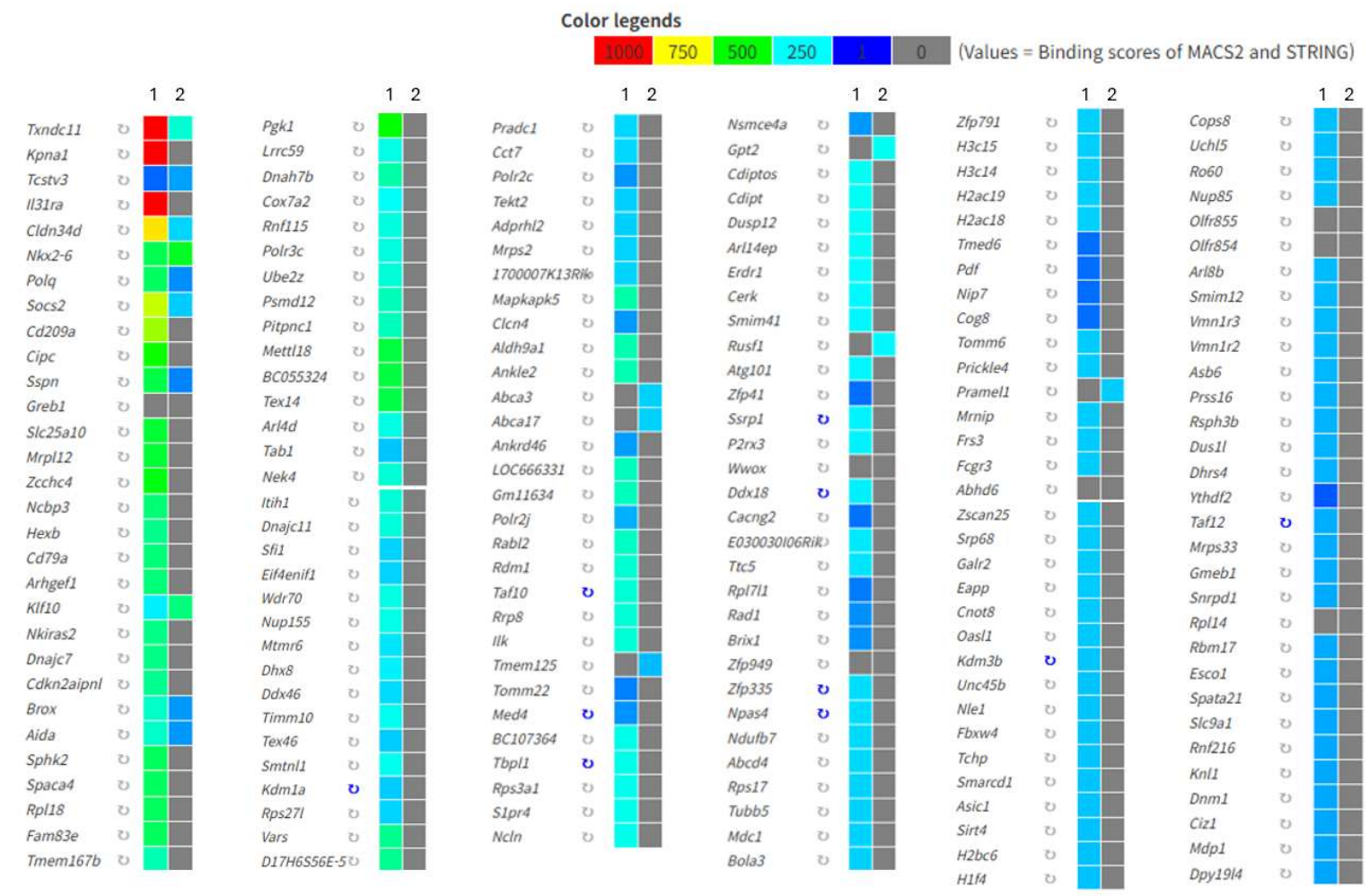

Representative comparison of BBX ChIP-seq signal quality between the two proliferative-condition samples, GSM3019272 and GSM3019273. Each gene is shown with two columns: column 1 represents GSM3019272, and column 2 represents GSM3019273. Compared with GSM3019272, GSM3019273 showed substantially weaker and less consistent BBX ChIP-seq signals across the examined loci.

As the main claim of the paper is that in the context of cortical development, BBX does not act as a transcription factor but a transcriptional regulator through its interaction with p53, among the 400 DEGs upon BBX knock-down, how many were p53 targets? This should be discussed and a list should be provided if available.

> We agree that this is an important and useful suggestion for further examining the relationship between BBX and p53. In the original manuscript, we identified 408 differentially expressed genes (DEGs) using the criteria of absolute fold change ≥ 2 and raw *P* < 0.05. In the revised manuscript, to more comprehensively evaluate the overlap between BBX-regulated genes and annotated p53 target genes, we reanalyzed the RNA-seq data using a less stringent but still biologically meaningful fold-change cutoff, while applying multiple-testing correction: an absolute fold change ≥ 1.5 and a Benjamini–Hochberg-adjusted *P* value < 0.05. We considered the original 2-fold cutoff to be relatively

conservative for this purpose, because RNA-level changes of 1.5-fold can represent meaningful transcriptional alterations, particularly when assessing pathway-related gene sets such as p53 target genes. Using these revised criteria, we identified 1,356 DEGs, among which 23 were annotated p53 target genes listed in the FISCHER_DIRECT_P53_TARGETS_META_ANALYSIS gene set from MSigDB.

> We have included these results in the revised Results section and provided the list of these 23 genes in Table EV1. We have also incorporated this point into the revised Results and Discussion sections, noting that these findings are consistent with the possibility that BBX depletion influences p53 transactivation activity at a subset of p53-responsive genes.

6/ Experiments related to HMG box domain and p53 interaction: As one of the main claims of the paper is that BBX function during cortical development is independent of its DNA binding ability, the authors should show that the delta HMG construct does indeed not bind to DNA.

> We agree with the referee that it is important to experimentally assess whether deletion of the predicted HMG box domain of BBX affects the DNA-associated properties of BBX. HMG box-containing proteins can interact with DNA or chromatin through diverse modes, including recognition of distorted DNA structures, association with promoter regions without clearly defined sequence specificity, or sequence-specific DNA binding. Although the precise mode by which the BBX HMG box domain engages DNA remains unclear, these possibilities all imply chromatin association. We therefore first examined whether the predicted HMG box domain contributes to the chromatin association of BBX.

> To address this point, we performed cellular fractionation analysis and found that HMG box-deleted BBX was substantially reduced in the chromatin-associated fraction compared

with wild-type BBX (Fig. 1D). These results indicate that the HMG box domain of BBX is required, at least in part, for efficient chromatin association. Accordingly, we have revised the relevant text to avoid overstatement and now describe the effect of HMG box deletion as impaired chromatin association rather than as direct loss of DNA binding.

The interaction between BBX and p53 is shown in HEK cells overexpressing the constructs. This data should be supported by showing that the interaction takes place in vivo using cortical samples or in neurospheres cultures either by Co-IP or a PLA assay.

> We agree with the referee that it is important to determine whether the BBX–p53 interaction occurs at the endogenous level in a physiologically relevant context. To address this point, we performed endogenous coIP using E13.5 mouse cortical tissue lysates. BBX was immunoprecipitated with an anti-BBX antibody, and co-immunoprecipitated p53 was detected by Western blotting with an anti-p53 antibody. This analysis confirmed endogenous BBX–p53 interaction in the developing cortex, and we have included these data in Fig. 4B of the revised manuscript.

It would be very nice if the authors could identify the region of BBX that interacts with p53 and show that BBX overexpression that cannot bind p53 does not effect cortical development. Yet, given the size of the two proteins, it could be hard to define the region of interaction using deletion constructs.

> Following the referee's suggestion, we generated a series of BBX truncation mutants and examined their interaction with p53 by coIP (Fig. EV3A and B). This analysis identified the BBX 334–668 mutant as the largest retained BBX fragment with little or no detectable p53-binding activity. We then tested this p53-binding-defective mutant by in utero electroporation

and found that, unlike wild-type BBX, it did not induce retention of electroporated cells in the VZ/SVZ and instead showed a distribution pattern comparable to that of control cells (Fig. EV3C). These results indicate that the interaction with p53 is required for BBX-mediated regulation of cortical cell distribution. We have added these data to Fig. EV3 and revised the Results section accordingly.

Referee #2:

This study reveals the essential role of BBX in the maintenance of embryonic neural stem cells (NSCs), cortical neuron migration, and laminar organization, and proposes a relatively novel mechanism whereby BBX suppresses p21 transcription through interaction with p53, thereby preventing premature cell-cycle exit and senescence of NSCs. Overall, the experimental strategy is well designed, combining both in vitro and in vivo approaches, with a clear logical progression from functional characterization to mechanistic analysis and rescue experiments. However, several key scientific issues remain insufficiently clarified or require further strengthening:

> Before addressing the individual comments, we greatly appreciate the referee's thoughtful comments and constructive suggestions. The referee's comments helped us substantially strengthen the manuscript by prompting additional transcriptomic analysis, clearer interpretation of the BBX overexpression and knockdown phenotypes, further in vivo validation of senescence-associated changes, and more detailed Methods descriptions. We are grateful for these insightful suggestions, which have significantly improved the mechanistic depth, clarity, and overall rigor of the revised manuscript. Our point-by-point responses are provided below.

1)Insufficient mechanistic explanation for the dispensability of the HMG domain in BBX. Perform ChIP-qPCR analysis in NSCs comparing wild-type BBX and the HMG-deleted mutant (BΔHMG) to determine whether their occupancy at the p21 promoter is similarly dependent on p53 rather than direct DNA binding.

> We thank the referee for raising this important mechanistic point. Following the referee's suggestion, we performed ChIP-qPCR analysis in NSCs to compare the occupancy of wild-type BBX and the HMG-deleted mutant BΔHMG at the p21 promoter. As shown in Fig. 4D,

BΔHMG was recruited to the p21 promoter at a level comparable to that of wild-type BBX. These results indicate that BBX occupancy at the p21 promoter does not require the HMG domain, supporting the idea that BBX is recruited to this locus through a mechanism distinct from direct HMG domain–mediated chromatin binding

2)Insufficient evidence that p21 is the "primary" or "exclusive" functional downstream target of BBX. Use RNA-seq data to distinguish p21-dependent versus p21-independent differentially expressed genes (DEGs), for example by performing rescue RNA-seq analysis under shBBX + shp21 conditions.

> Following the referee's suggestion, we performed rescue RNA-seq analysis to distinguish p21-dependent and p21-independent BBX-regulated DEGs. Because this rescue experiment required simultaneous BBX knockdown and p21 co-knockdown, we used a dual-vector lentiviral design and compared shControl + shControl, shBBX + shControl, and shBBX + shp21 samples. This strategy was also used throughout the study for other experiments involving simultaneous manipulation of two effectors, allowing the input of the shBBX or BBX vector to be matched between the corresponding control and co-manipulation conditions. In the rescue RNA-seq experiment, this design allowed the shBBX viral input to be matched between the shBBX + shControl and shBBX + shp21 conditions.

> We note, however, that this rescue RNA-seq design differs from the original single-vector shControl versus shBBX RNA-seq experiment. In the dual-vector design, the relative amount of shBBX virus delivered to the shBBX + shControl sample would be expected to be lower than that used in the original single-vector shBBX condition, which may have attenuated the magnitude of BBX knockdown-induced transcriptional changes. Moreover, because the expression of some BBX-responsive genes may not change linearly with BBX

levels but instead respond only when BBX expression falls below a specific threshold, the reduced shBBX input could also have altered the composition of the DEG set itself.

> In the revised manuscript, we reanalyzed the original single-vector RNA-seq dataset using a less conservative but still biologically meaningful fold-change cutoff of absolute fold change ≥ 1.5, together with a Benjamini–Hochberg-adjusted P value < 0.05 (Fig. 3A-D). This revised threshold allowed us to perform a broader assessment of BBX knockdown-associated transcriptional changes while maintaining statistical stringency through multiple-testing correction.

> Consistent with the differences between the single-vector and dual-vector RNA-seq designs described above, application of these revised DEG criteria—absolute fold change ≥ 1.5 and adjusted P value < 0.05—yielded a relatively small number of DEGs in the rescue RNA-seq dataset. Therefore, for this dual-vector-based comparative rescue analysis, we used a less stringent fold-change cutoff of absolute fold change ≥ 1.3 while retaining an adjusted P value < 0.05.

> Using this approach, we first identified genes altered by BBX knockdown in the shControl + shControl versus shBBX + shControl comparison. Among these BBX-regulated genes, those whose expression changes were reversed by p21 co-knockdown were classified as p21-dependent DEGs. This analysis identified 41 p21-dependent DEGs in total (Fig. EV5A and B). Among these, 17 genes were upregulated by shBBX and reduced by shp21 co-knockdown, whereas 24 genes were downregulated by shBBX and restored by shp21 co-knockdown. The expression patterns of these 41 p21-dependent DEGs are shown as a heatmap in Fig. EV5C. GO functional enrichment analysis of these genes revealed enrichment of terms related to developmental processes, biological regulation, and responses to stimuli (Fig. EV5D). In parallel, BBX-regulated genes whose altered

expression was not reversed by p21 co-knockdown were classified as p21-independent DEGs. Using this classification, we identified 328 p21-independent DEGs (Fig. EV5A).

> Importantly, this analysis indicates that BBX knockdown induces both p21-dependent and p21-independent transcriptional changes. We therefore agree with the referee that p21 should not be described as the primary or exclusive downstream target of BBX. Accordingly, we have revised the text from "a major downstream mediator" to "an important functional downstream mediator" and changed the Figure 6 title from "p21 serves as a critical downstream target of BBX" to "p21 mediates key BBX-dependent phenotypes." These revisions acknowledge that, although p21 mediates key functional consequences of BBX depletion, it mediates only a specific subset of BBX-regulated transcriptional changes. We have added the rescue RNA-seq analysis to Fig. EV5 and revised the Results section accordingly.

3)Potential confusion caused by apparently similar phenotypes induced by BBX knockdown and BBX overexpression. Include quantitative analyses of cell markers (e.g., NeuN, SATB2, CTIP2) to clearly distinguish the fundamentally different biological outcomes of BBX overexpression versus depletion.

> We agree with the referee that the superficially similar distributional phenotypes induced by BBX overexpression and BBX knockdown could cause confusion and should be distinguished more clearly. Because our data indicate that BBX regulates p21-dependent cell-cycle and progenitor-state changes, we focused primarily on progenitor markers in the proliferative VZ/SVZ, including SOX2 and PAX6. We also performed SATB2 staining as an upper-layer CP neuronal marker, as suggested by the referee.

> Although both BBX overexpression and BBX knockdown shifted GFP$^+$ cells away from

the CP and toward the VZ/SVZ in the E14.5 > E17.5 setting, marker analyses indicated that the underlying cellular states were distinct. BBX overexpression increased the proportions of SOX2$^+$ and PAX6$^+$ progenitor-marker-positive cells in the VZ/SVZ and reduced SATB2$^+$ upper-layer neurons, consistent with progenitor retention and/or expansion and delayed neuronal differentiation or CP entry. In contrast, BBX knockdown reduced SOX2$^+$, Cyclin D1$^+$, and PAX6$^+$ cells within the GFP$^+$ VZ/SVZ population, indicating that the accumulation of BBX-depleted cells in the VZ/SVZ does not reflect enhanced progenitor maintenance. Rather, these findings suggest that BBX-depleted cells undergo premature cell-cycle exit but fail to migrate efficiently out of the progenitor zone, consistent with the broader migration defects observed upon BBX knockdown. In addition, BBX knockdown did not significantly increase the proportion of SATB2$^+$ cells. This may reflect opposing consequences of p21 activation: increased cell-cycle exit could facilitate aspects of neuronal differentiation, whereas senescence-associated changes may impair maturation of immature DCX$^+$ neurons toward a SATB2$^+$ upper-layer identity, resulting in little net change in SATB2$^+$ cells. Thus, BBX overexpression and knockdown produce superficially similar distributional phenotypes through distinct biological mechanisms.

> We have added these new marker analyses and the corresponding interpretation to the revised Results and Discussion sections (Fig. EV2B–F).

> We also performed CTIP2 staining, as suggested by the referee. However, under the E14.5 > E17.5 electroporation setting used in this analysis, CTIP2 showed little overlap with GFP$^+$ electroporated cells in either the BBX overexpression or knockdown condition. This is likely because CTIP2 primarily marks earlier-born deep-layer projection neurons, whereas cells electroporated at E14.5 predominantly contribute to later-born upper-layer neuronal populations, for which SATB2 is a more informative marker. Therefore, CTIP2 staining did not provide a useful basis for distinguishing the BBX overexpression and knockdown

phenotypes in this experimental setting, and we focused our quantitative analysis on progenitor markers in the VZ/SVZ and SATB2 as an upper-layer neuronal marker.

4)Insufficient direct evidence for true senescence in postmitotic neurons. Although the RB rescue experiments support the involvement of senescence, there is currently no direct evidence demonstrating that postmitotic neurons undergo bona fide senescence. Perform co-localization analysis of SA-β-gal activity or p21/γH2AX expression with DCX^{+} or $TBR1^{+}$ neurons.

> We agree with the referee that it is important to determine whether senescence-associated changes occur in postmitotic neuronal populations in vivo. To address this point, we performed γH2AX staining after in utero electroporation of the shBBX construct, as suggested by the referee.

> In the original manuscript, we showed that the abnormal cell clusters accumulating in the IZ of shBBX-electroporated cortices were composed largely of DCX^{+} cells. In the revised manuscript, we now show that γH2AX signals were readily detected in these IZ cell clusters in shBBX-electroporated cortices, whereas such signals were rarely observed in control samples (Fig. 5G). In addition, γH2AX-positive signals were also observed in the CP region (Fig. 5H). These findings provide in vivo evidence that BBX-depleted cortical cells exhibit a senescence-associated DNA damage response. We have added these data to Fig. 5G and H and revised the Results section accordingly.

5)In Figure 1, detection of endogenous BBX protein levels should be included for both BBX overexpression constructs and BBX-targeting shRNAs to validate the efficiency of overexpression and knockdown.

> Following the referee's suggestion, we included Western blot analyses to validate BBX protein levels under both overexpression and knockdown conditions. BBX overexpression was confirmed at the protein level in Fig. EV1A, and the knockdown efficiency of the BBX-targeting shRNAs was validated at the protein level in Fig. 1H. We have revised the Results section accordingly.

6)In Figure 2, panel 2B lacks quantitative statistical analysis, which should be provided.

> Following the referee's comment, we quantified the phenotype shown in Fig. 2B and added the corresponding graph and statistical analysis to the revised figure.

7)In Figure 3, clarification is required regarding the RNA-seq sampling strategy: Were samples collected from electroporated cortical regions? What proportion of the tissue consisted of GFP^{+} cells? What was the estimated percentage of BBX-depleted cells?

> We apologize for the lack of clarity. The RNA-seq samples used in Fig. 3 were not collected from electroporated cortical regions. Instead, RNA-seq was performed using E13.5 NSCs transduced *in vitro* with shBBX lentivirus. Although this was stated in the original Results and Methods sections, we agree that the sampling strategy was not sufficiently clear. We have therefore revised the Methods section to state more explicitly that NSCs isolated from E13.5 embryonic cortices were transduced with shBBX #2 or control lentivirus, cultured for 3 days, and harvested for total RNA extraction using RNAiso Plus reagent. Thus, the proportion of GFP^{+} cells within electroporated cortical tissue is not applicable to this experiment.

> Because both the control shRNA and shBBX lentiviral vectors express GFP as a reporter, we confirmed efficient transduction by fluorescence microscopy. Using concentrated high-

titer lentiviral vectors, GFP expression was observed in nearly all cultured NSCs, indicating that the RNA-seq samples were highly enriched for vector-transduced cells. To make this point clearer, we have revised the Methods subsection title from “Bioinformatics analysis” to “RNA-seq and bioinformatics analysis” and added a more explicit description of the RNA-seq sampling strategy in the revised Methods section.

8)In addition, conditional knockout mouse models would provide more robust and reliable results than mosaic knockdown approaches.

> We appreciate the referee’s point and agree that conditional knockout mouse models would provide robust and reliable genetic evidence. At the same time, in utero electroporation remains a useful mosaic approach for assessing cell-autonomous effects of gene manipulation in defined populations of cortical cells during embryonic development. Although generating and validating a conditional knockout mouse model is not feasible within the time frame of the current revision, we have strengthened the study by adding rescue and mechanistic experiments that support the specificity and mechanistic interpretation of the BBX knockdown phenotype.

Referee #3:

The authors propose that BBX functions as a differentiation suppressor among transcription factors involved in the fate determination of neural stem cells. Through in vitro primary culture experiments involving overexpression and knockdown of BBX, they demonstrate that BBX promotes neural stem cell properties such as neurosphere formation and Sox2 expression. In vivo, using in utero electroporation, they show that BBX is important for maintaining Sox2+ cells in the VZ and also for neurite formation after differentiation. Analyses of RNA-seq and public ChIP-seq data indicated that p21 might be a target, and mechanistically, the authors propose, based on co-IP and reporter assays, that BBX inhibits p53 activity in a DNA-binding-independent manner.

Overall, the manuscript provides many experimental data and propose interesting conclusions. However, several important experimental conditions and protocols are insufficiently described, and the current data do not fully support the conclusions. Therefore, substantial revisions are necessary before the manuscript can be considered for publication.

> As an overall response, we would first like to sincerely thank the referee for the careful evaluation of our manuscript and for the helpful suggestions. These comments helped us substantially expand the main text and Methods section by adding more detailed descriptions of the experimental procedures and reagents used in this study. The referee's suggestions also helped us replace simple descriptive statements and ambiguous wording with more data-driven explanations, including quantitative analysis of cell morphology and additional in vivo validation of senescence-associated changes. We appreciate these insightful comments, which have improved the methodological detail, analytical rigor, and clarity of the revised manuscript.

Major comments

1. There is a lack of detailed description of experimental timelines and procedures, making it difficult to determine whether the authors' conclusions are appropriately supported. The authors should provide detailed methods sufficient for reproducibility. The following specific points should be addressed:

o The experimental timelines and methodologies - for example, the timing of gene introduction and induction of differentiation in Fig. 1G, which is critical for distinguishing effects on NSC fate determination from those on neurite outgrowth - should be explicitly described for each experiment.

> We agree with the referee that the experimental timelines and methodologies should be described more explicitly, particularly for the neuronal differentiation experiment shown in Fig. 1G. In the revised Methods section, we have provided a more detailed description of the NSC lentiviral transduction and neuronal differentiation protocols, including the timing of gene introduction, the duration of culture after transduction, and the induction of differentiation by FGF2 withdrawal.

> We have also expanded the Methods section for other experiments to improve clarity and reproducibility.

o Details regarding plasmid construction for BBX, BΔHMGs, and shRNA are missing (e.g., primers used, backbone plasmids, cloning strategy, or references to commercially available plasmids). The Methods should clarify how GFP is incorporated into samples in each experiment.

> We apologize that these details were not sufficiently described in the original manuscript. Following the referee’s comment, we have expanded the Methods section to include

additional information on the cloning strategies for BBX, BΔHMG, and other constructs, as well as the shRNA vectors and backbone plasmids used in this study. The primers used for plasmid construction and the shRNA target sequences are also listed in the Reagents and Tools Table.

> We have also clarified how GFP was incorporated into each experimental system in the revised Results and Methods sections. The pWPI and pCAGIG vectors contain an IRES-GFP cassette, whereas the pLKO.3G vector expresses GFP from the hPGK promoter. Thus, all gene delivery vectors used in this study allow gene-transferred cells to be identified by GFP expression from the same vector, without the use of a separate GFP-expressing plasmid or virus.

o The methods used for DEG analysis should be described in detail, including the software employed, normalization methods, etc. The criteria for selecting the presented GO terms for figures (Fig 3C-D) should also be indicated.

> We thank the referee for pointing out that the RNA-seq analysis methods and GO term selection criteria were not described in sufficient detail. In the original manuscript, DEGs were identified using the criteria of fold change ≥ 2 and raw $P < 0.05$. In the revised analysis, we used a less conservative but still biologically meaningful fold-change cutoff of absolute fold change ≥ 1.5, together with a Benjamini–Hochberg-adjusted P value < 0.05. This revised threshold allowed us to perform a broader assessment of BBX knockdown-associated transcriptional changes while maintaining statistical stringency through multiple-testing correction.

> Using these revised criteria, we identified 1,356 DEGs. GO Biological Process enrichment analysis was then performed using these 1,356 DEGs. To improve readability and facilitate

comparison among enriched GO terms, we revised Fig. 3C from a dot plot to a bar graph showing enrichment significance as −log10(P). This format allows the relative significance of the enriched biological processes to be more directly visualized.

> For Fig. 3D, the displayed functional categories were selected based on the phenotypes observed upon BBX knockdown. Cell-cycle regulation and proliferation-related genes were examined because BBX knockdown reduced in vitro cell growth (Fig. EV1G), decreased Cyclin D1 protein levels (Fig. 1J), and reduced the number of Cyclin D1^{+} cells in vivo (Fig. 2E). Senescence-related genes, including those involved in DNA damage and repair responses, were examined because BBX-depleted neurospheres were not only reduced in size but also displayed morphologically compromised features suggestive of cellular stress (Fig. 1I), despite no detectable increase in cleaved caspase-3 levels (Fig. EV1H). Within these categories, only genes that satisfied the revised DEG criteria were displayed in Fig. 3D.

o The catalog number and the provider should be given for all antibodies used, such as Sox2 and γH2AX.

> This information was not sufficiently accessible in the original submission because the Reagents and Tools Table was inadvertently omitted. In the revised manuscript, we have added a complete Reagents and Tools Table that includes the provider, catalog number, and working dilution for all antibodies used in this study, including those against SOX2 and γH2AX.

o The Methods should specify the cell type used for luciferase and ChIP assays, specifically in the Results section corresponding to Fig. 4.

> We have revised the relevant Results sections, figure legends, and Methods descriptions to explicitly specify the cell types used for the luciferase and ChIP assays. The revised manuscript now states that the luciferase reporter assays were performed in U87MG cells, whereas the ChIP assays were performed in NSCs.

o Please explain the nature of the p53DN construct.

> We agree that the nature of the p53DN construct should be explained more clearly. In the revised manuscript, we clarified that this construct was originally designated p53DD, but is referred to as p53DN in this study to indicate its dominant-negative activity. We also added a description explaining that p53DN inhibits endogenous p53 function by interfering with wild-type p53 DNA binding through heterotetramerization, thereby suppressing p53-dependent target gene activation. The relevant references and plasmid source, including the Addgene catalog number, have also been added to the Methods section.

o For RT-qPCR, describe the reverse transcription conditions and methodology.

> Following the referee's comment, we have expanded the RT-qPCR Methods section to describe the reverse transcription conditions and experimental procedure in greater detail. The revised Methods now specify the amount of RNA used for cDNA synthesis, the reverse transcription reagent and reaction conditions, and the qPCR procedure used to quantify gene expression. We have also clarified the normalization method used for RT-qPCR analysis.

2. As BBX knockdown phenotypes, the authors report: 1) reduced VZ cells, 2) reduced Sox2+ cells, 3) reduced CycD1, 4) increased cell cycle exit, 5) decreased Dcx+, and 6)

immature neurites. However, phenotypes 1-4 appear inconsistent with phenotype 5. The authors should discuss explicitly how each cell population at various stages of differentiation is affected.

> We agree that the apparent inconsistency between the reduced progenitor-related phenotypes (#1-4) and the reduced DCX⁺ neuronal differentiation (#5) required a clearer explanation. Phenotypes 1–4 were obtained in the in vivo cortical context, whereas the reduction in DCX⁺ cells was observed in the in vitro differentiation assay. Thus, these results should not be interpreted as direct one-to-one counterparts.

> In the developing cerebral cortex, increased p21 expression has been shown to promote cell-cycle exit (Siegenthaler and Miller, 2005). Therefore, our in vivo findings—reduced VZ cells, reduced SOX2⁺ progenitors, reduced Cyclin D1 expression, and increased cell-cycle exit—are consistent with activation of a p21-dependent cell-cycle inhibitory program upon BBX knockdown. These findings indicate that BBX depletion reduces the proliferative progenitor population and promotes premature cell-cycle exit in the cortical germinal zone.

> By contrast, in the in vitro differentiation assay, removal of growth factors suppresses proliferation and promotes differentiation in both shControl and shBBX samples. Thus, both groups are functionally driven toward a cell-cycle-exited differentiation state by the culture condition itself, which may mask the additional cell-cycle-inhibitory effect of shBBX-induced p21 activation. Instead, the senescence-associated state induced by BBX knockdown may become the dominant factor affecting neuronal differentiation. Indeed, as shown in Fig. 5C, a substantial fraction of BBX-depleted NSCs already exhibited detectable SA-β-gal activity under proliferation conditions. Such a compromised cellular state may impair the transcriptional, translational, metabolic, and differentiation programs required for efficient generation of DCX⁺ neurons and subsequent neurite maturation.

> We have added this interpretation to the revised Discussion section to distinguish the in vivo progenitor phenotypes from the in vitro differentiation phenotype.

3. Experiments with the HMG box mutant are critical to support the authors' claims about BBX molecular function. Thus, a precise description of the mutant (is it a point mutation, an HMG box deletion, and if so, which residues?) is essential. It is also necessary to demonstrate that this mutant truly lacks DNA-binding activity.

> We apologize that the description of the BBX HMG box deletion mutant, BΔHMG, was not sufficiently clear in the original manuscript. BΔHMG is an HMG box deletion mutant, not a point mutant, in which amino acids 80–148 were removed from full-length BBX. We have now specified this information in the Results section and added the corresponding construction strategy to the Methods section.

> We also agree with the referee that it is important to experimentally assess whether this mutant is impaired in DNA-associated activity. HMG box-containing proteins can interact with DNA through diverse modes, including recognition of distorted DNA structures, association with promoter regions without clearly defined sequence specificity, or sequence-specific DNA binding. Because the precise DNA-interaction mode of the BBX HMG box domain remains undefined, and because these modes of DNA interaction are expected to involve chromatin association in the cellular context, we examined whether deletion of this domain affects the association of BBX with chromatin. We have added this rationale to the Results section of the revised manuscript.

> To address this point, we performed cellular fractionation analysis and found that HMG box-deleted BBX was substantially reduced in the chromatin-associated fraction compared with wild-type BBX (Fig. 1D). These results indicate that the HMG box domain of BBX is

required, at least in part, for efficient chromatin association. Accordingly, we have revised the relevant text to avoid overstatement and now describe the effect of HMG box deletion as impaired chromatin association rather than as direct loss of DNA binding. We believe that this revised description more accurately reflects the experimental evidence provided by the fractionation assay.

4. As also noted above, the experiments in Fig. EV1C and Fig. 1G need to distinguish clearly between suppression of differentiation versus suppression of neurite outgrowth.

> We agree with the referee that neuronal differentiation and neurite outgrowth should be distinguished more clearly. Fig. EV1I (originally Fig. EV1C) assesses the generation of DCX^+ neuronal cells under in vitro differentiation conditions, whereas Fig. 1L (originally Fig. 1G) examines neurite length and branching in DCX-stained neuronal cells. We have therefore revised the relevant Results text to state that BBX supports neuronal differentiation and subsequent neurite outgrowth, rather than referring broadly to "neurogenic potential."

In Fig. 1G, it appears that NSCs as well as neurons are present in the images; thus, neuronal neurites should be quantified using neuronal markers. Furthermore, when quantifying dendritic branches, how are dendrites distinguished from axons?

> Following the referee's suggestion, we repeated this experiment by co-immunostaining for GFP, which marks gene-transferred cells, and DCX. We then quantified neurite morphology specifically in GFP^+/DCX^+ neuronal cells. These revised data are now presented in Fig. 1L (originally Fig. 1G).

> We also agree with the referee that dendrites and axons cannot be reliably distinguished in this assay without axon- or dendrite-specific marker staining. In our original analysis, neurite morphology was assessed based on GFP^+ processes, and the longest neurite was used as a readout of overall neurite extension. However, because we did not perform axon- or dendrite-specific marker staining in this experiment, we have revised the terminology in the manuscript and figure labels from “dendritic branches” to “primary neurite branches”. The revised analysis therefore describes overall neurite outgrowth and branching rather than dendrite-specific morphology.

5. Regarding Fig. 3D: among the genes included, which are DEGs? There are more genes involved in cell proliferation/inhibition, DNA damage response, and senescence-what criteria were used to select genes shown in this figure? What does "a clear correlation with BBX depletion" mean? Also, showing Cdkn1a twice in the same figure is misleading.

> We appreciate the referee’s careful comment on the gene selection criteria and presentation of Fig. 3D. In the original version, we displayed representative genes associated with cell proliferation, cell-cycle inhibition, DNA damage response, and senescence, but we agree that this did not clearly explain why those genes were selected.

> In the revised manuscript, we reanalyzed the RNA-seq data and redrew Fig. 3D to display all genes within the indicated functional categories that satisfied the DEG criteria of absolute fold change ≥ 1.5 and adjusted $P < 0.05$, rather than a manually selected subset of representative genes. Specifically, we extracted gene sets associated with the BBX knockdown phenotypes, including cell-cycle regulation, senescence, and DNA damage response, from the Molecular Signatures Database (MSigDB). We have clarified these selection criteria in the revised Results section and figure legend.

> We also agree that the phrase "a clear correlation with BBX depletion" was not sufficiently precise. We intended to indicate that *Cdkn1a* (p21) showed prominent induction upon BBX knockdown, consistent with the observed decrease in Cyclin D1 expression and reduced cell proliferation. To avoid ambiguity, we have revised the text by replacing the phrase "a clear correlation with BBX depletion" with the more precise statement, "Among the cell-cycle inhibitory regulators examined, *Cdkn1a* was the only gene that showed robust upregulation upon BBX knockdown."

> Finally, we agree that showing *Cdkn1a* twice in the same figure could be misleading. In the revised Fig. 3D, we removed the separate cell-cycle inhibitor category and no longer display *Cdkn1a* more than once.

6. The Results section should include interpretation of findings rather than only phenotypic descriptions.

> We agree with the referee that the Results section should provide clearer interpretation of the findings, rather than presenting only phenotypic descriptions. In the original manuscript, we tried to avoid overstatement and therefore described several results in a conservative manner. However, we recognize that this made the biological implications of some findings less clear. In the revised manuscript, we have added interpretive statements throughout the Results section to clarify how each set of data supports our conclusions regarding BBX function in NSCs, cortical development, and the p53–p21 pathway.

7. For Fig. 4E, please indicate the p-value between control and knockdown for the p21p-mt sample. If it is significantly increased, this would suggest that the effect of BBX is only partial, so this possibility should be discussed thoroughly.

> Following the referee's suggestion, we indicated the *P* value for the comparison between shControl and shBBX in the p21p(mt) condition in Fig. 4H (originally Fig. 4E). Although the p21p(mt) reporter showed a slight trend toward increased activity upon BBX knockdown, this difference was not statistically significant (*P* = 0.0628). Thus, mutation of the p53-binding motif largely abolished the effect of BBX knockdown on p21 promoter activity, supporting our conclusion that BBX regulation of the p21 promoter depends primarily on the p53 response element within this promoter region.

8. The phrase "the physiological integrity of immature neurons" is unclear-what aspect of "integrity" is intended here?

> We agree with the referee that the phrase "physiological integrity of immature neurons" was unclear. We intended to refer to the cellular programs required for immature neurons to undergo proper migration, polarization, and neurite remodeling. To avoid ambiguity, we have revised the text to describe these processes more directly.

> The original sentence read: "Aberrant p21 upregulation caused by BBX depletion disrupts the physiological integrity of immature neurons, impairing their migration toward the CP and resulting in their ectopic accumulation within the IZ."

The revised sentence now reads: "Aberrant p21 upregulation caused by BBX depletion disrupts the migration and morphological maturation of immature neurons, thereby contributing to their ectopic accumulation within the IZ."

Minor comments

9. In the Abstract, "BBX suppression of p21" is ambiguous; it should be "suppression of p21 by BBX".

> We agree that the phrase “BBX suppression of p21” was ambiguous. In response to the referee’s comment, we substantially rewrote the Abstract to incorporate the newly added data and to minimize ambiguity. For example, we revised “the diverse phenotypic alterations caused by BBX knockdown” to “BBX knockdown-induced defects in NSC maintenance, neuronal distribution, and morphology.” As part of this revision, the ambiguous phrase was removed rather than directly replaced with “suppression of p21 by BBX.” The revised Abstract now describes BBX-mediated regulation of p21 more clearly in the context of the p53–p21 pathway.

10. The authors state that BBX is expressed only in the VZ; have they confirmed this themselves? Figs. 1 and 2 of Dixon, 2013 do not make it clear whether BBX is specifically expressed in the neocortex VZ.

> We thank the referee for this careful comment. We agree that the expression pattern of BBX in the developing neocortex should be clearly documented to establish the rationale for this study. Although Dixon et al. (2013) reported BBX expression in the developing brain, the figures in that study do not unambiguously demonstrate VZ-specific expression in the neocortex. To address this point, we have included an in situ hybridization image of *Bbx* from the GenePaint database in Fig. 1A. This image shows prominent *Bbx* expression along the germinal zones of the developing cortex, including the VZ and SVZ. Accordingly, we have revised the Introduction and other relevant text to describe BBX expression as enriched in the germinal zones of the developing cortex, including the VZ/SVZ, rather than as being restricted to the VZ.

11. The descriptors "round" and "irregular" in Fig. EV2B are too subjective; more precise

definitions and clear quantification criteria should be provided.

> We agree with the referee that the criteria for classifying “round” and “irregular” morphologies should be defined more clearly. To address the referee’s concern regarding subjectivity, we replaced the categorical classification with a quantitative analysis using the circularity index, a well-established metric for assessing cell or nuclear shape (Ankam et al., 2018; Boi et al., 2023).

> Morphological analysis was performed on GFP-immunostained tissue sections. GFP^+ cell bodies were traced in ImageJ, and circularity was calculated as $4\pi \times area/perimeter^2$. This index ranges from 0 to 1, with a value of 1 representing a perfect circle and lower values indicating increasingly elongated or irregular shapes. Using this objective measurement, we found that BBX knockdown significantly reduced the circularity index of GFP^+ cells, supporting the conclusion that BBX-depleted cells exhibit a more irregular cell body morphology. We have added this quantification to Fig. EV2G and clarified the analysis criteria in the revised Methods section and figure legend.

12. Was the GO analysis in Fig. 3C performed on up- or down-regulated genes? What does "shBBX / shControl" refer to in the figure?

> We appreciate the referee’s request for clarification regarding the GO analysis shown in Fig. 3C. In the original manuscript, GO enrichment analysis was performed using all DEGs identified in shBBX-transduced NSCs compared with shControl-transduced NSCs, including both upregulated and downregulated genes that satisfied the cutoff of absolute fold change ≥ 2 and raw $P < 0.05$.

> We agree that the original x-axis label “shBBX / shControl” was ambiguous and could cause confusion. This label was intended to indicate the gene ratio, namely the proportion

of DEGs associated with each GO term, but this was not clearly described. We also agree that the figure legend should have explicitly stated that the plot showed the top 10 enriched GO Biological Process terms ranked by adjusted *P* value, with dot size indicating the number of DEGs associated with each term and dot color indicating the adjusted *P* value.

> As described above, using the revised DEG criteria of absolute fold change ≥ 1.5 and adjusted $P < 0.05$, we identified 1,356 DEGs. GO Biological Process enrichment analysis was then performed using these 1,356 DEGs, including both upregulated and downregulated genes. To improve readability and facilitate comparison among enriched GO terms, we revised Fig. 3C from a dot plot to a bar graph showing enrichment significance as −log10(P). This format allows the relative significance of the enriched biological processes to be more directly visualized. We have also revised the figure legend accordingly to clarify the DEG set used for the analysis and the basis for displaying the enriched GO terms.

13. The statement "Given that BBX knockdown reduces cyclin D1 expression both in vitro and in vivo,"-is this previously known or shown in the RNA-seq results? If it is known, a citation is required.

> This statement was not sufficiently clear in the original manuscript. It was not based on previously published findings or inferred from the RNA-seq data. Rather, it referred to our own experimental results. Specifically, Fig. 1J (originally Fig. 1F) showed that BBX knockdown reduced Cyclin D1 protein levels in NSCs in vitro, and Fig. 2E showed that BBX knockdown reduced the number of Cyclin D1$^+$ cells in the embryonic cortex in vivo. To avoid ambiguity, we have revised the text to state that “Given that BBX knockdown reduces Cyclin D1 protein levels in vitro and decreases the number of Cyclin D1$^+$ cells in vivo”

14. Gene names should be italicized throughout, e.g., in Figs. 3D and 3E.

> We have revised the manuscript to italicize gene symbols where appropriate, including the gene names shown in Figs. 3D and 3E.

15. In Figs. 3E and 4A, gene structures such as exons, introns, and transcriptional orientation should be annotated. In Fig. 3E, a diagram of a known BBX-binding locus as positive control would be helpful. Were the "proliferation genes" in Fig. 3D examined for BBX binding?

> Following the referee's comment, we have revised Figs. 3E and 4A to include gene model annotations showing exon/intron organization and transcriptional orientation. In these genome browser views, boxes indicate exons, connecting lines indicate intronic regions, and arrows indicate the direction of transcription. This information has also been clarified in the corresponding figure legends.

> We agree with the referee that including a known BBX-binding locus as a positive control would be helpful. However, to our knowledge, despite the presence of an HMG box domain, BBX has not been well characterized as a transcription factor; consequently, its sequence-specific DNA-binding properties remain unclear, and no consensus BBX-binding motif has been defined. Accordingly, we are not aware of a validated BBX-binding locus that could serve as a generally accepted positive control for this analysis.

> We also examined BBX occupancy at the proliferation-related genes shown in Fig. 3D, but did not detect BBX occupancy at any of the examined loci.

16. U87MG, used in the reporter assay, is a human cell line; can mouse knockdown vectors

function effectively in this context?

> We apologize that this point was not clearly described in the original manuscript. Because U87MG is a human cell line, we did not use the mouse *Bbx*-targeting shRNA vector for the reporter assay. Instead, we used a separate human *BBX*-targeting shRNA vector, which reduced BBX expression by approximately 80% in U87MG cells (Fig. 4G). In the revised manuscript, we have clarified this point in the Results and Methods sections and provided the nucleotide sequence of the human *BBX* shRNA in the Reagents and Tools Table.

17. The manuscript suggests that BBX acts as a p53 cofactor and that overexpression results indicate MDM2 and GADD45A may also be targets. Is ChIP evidence for BBX binding at the MDM2 and GADD45A loci available?

> We agree that this is an important point. We inspected the publicly available BBX ChIP-seq dataset generated from trophoblast stem cells for BBX occupancy at the *Mdm2* and *Gadd45a* loci. However, we did not find sufficiently strong or reproducible BBX ChIP-seq evidence at these loci to support including them in the main mechanistic analysis.

> The proliferative-condition BBX ChIP-seq dataset contains only two GEO samples, GSM3019272 and GSM3019273. As shown below, GSM3019273 showed poor signal quality across most examined loci compared with GSM3019272, limiting reliable locus-by-locus interpretation. Under these conditions, *Cdkn1a* (p21) was one of the few loci showing BBX enrichment in both samples (Fig. 4A). In contrast, even in the relatively higher-quality GSM3019272 dataset, BBX ChIP-seq signals at the *Gadd45a* and *Mdm2* loci were weaker than those at the *Cdkn1a* (p21) locus. As shown in the representative tracks below, displayed at the same y-axis scale, the *Gadd45a* and *Mdm2* loci did not show prominent promoter-proximal enrichment comparable to that observed at the *Cdkn1a* (p21) locus.

Moreover, the corresponding signals at the *Gadd45a* and *Mdm2* loci were not sufficiently reliable in GSM3019273.

> In addition, although *Mdm2* and *Gadd45a* mRNA levels, similar to *Cdkn1a* (p21), were reduced upon BBX overexpression, they did not show a consistent reciprocal response upon BBX knockdown, which more directly assesses the requirement for endogenous BBX activity (Fig. EV3D and EV3E). In contrast, *Cdkn1a* (p21) was consistently upregulated upon BBX knockdown. Therefore, we focused our mechanistic analysis on *Cdkn1a* (p21), for which both transcriptional regulation and ChIP-seq occupancy were more consistently supported.

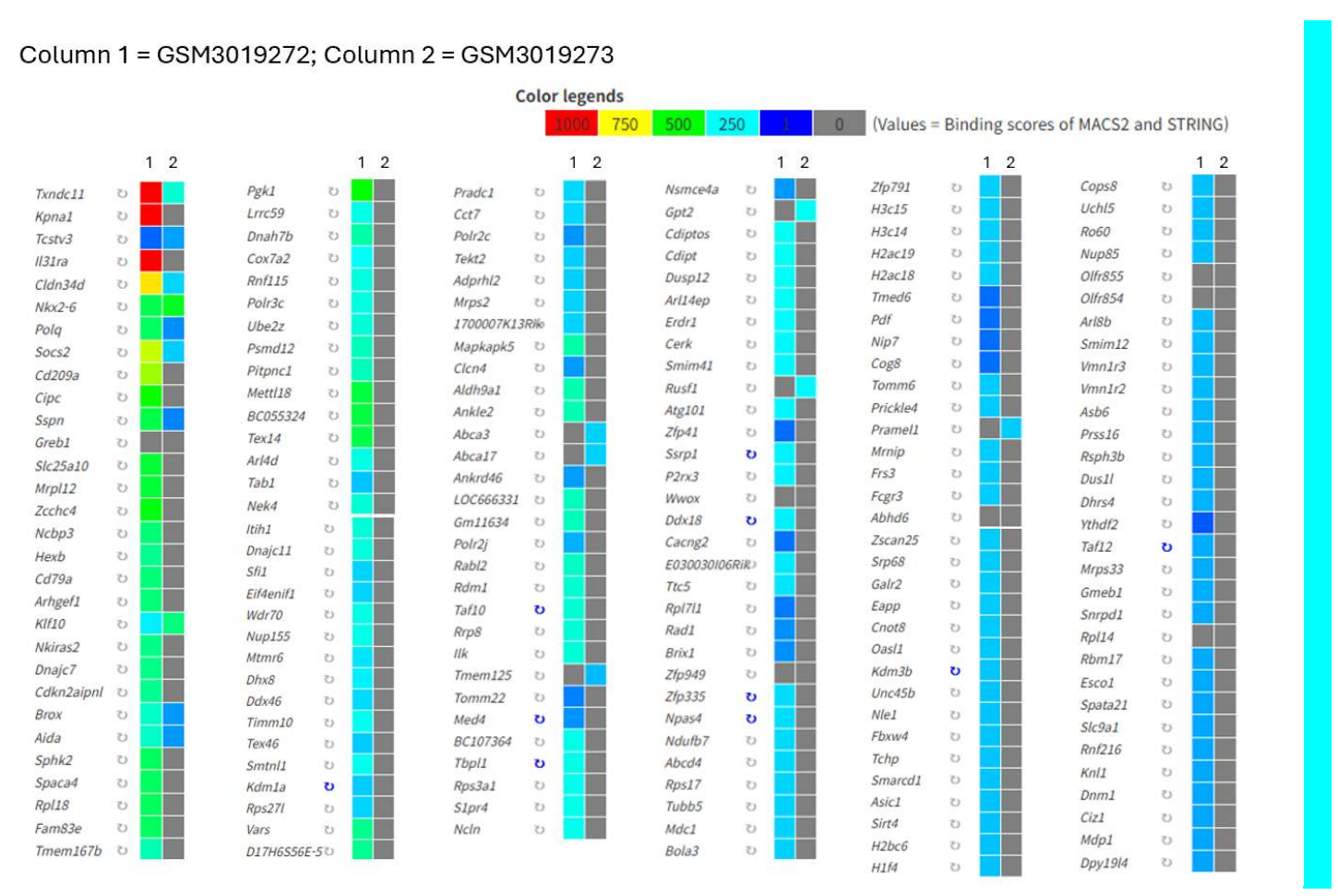

Representative comparison of BBX ChIP-seq signal quality between the two proliferative-condition samples, GSM3019272 and GSM3019273. Each gene is shown with two columns: column 1 represents GSM3019272, and column 2 represents GSM3019273. Compared with GSM3019272, GSM3019273 showed substantially weaker and less consistent BBX ChIP-seq signals across the examined loci, limiting reliable locus-by-locus interpretation.

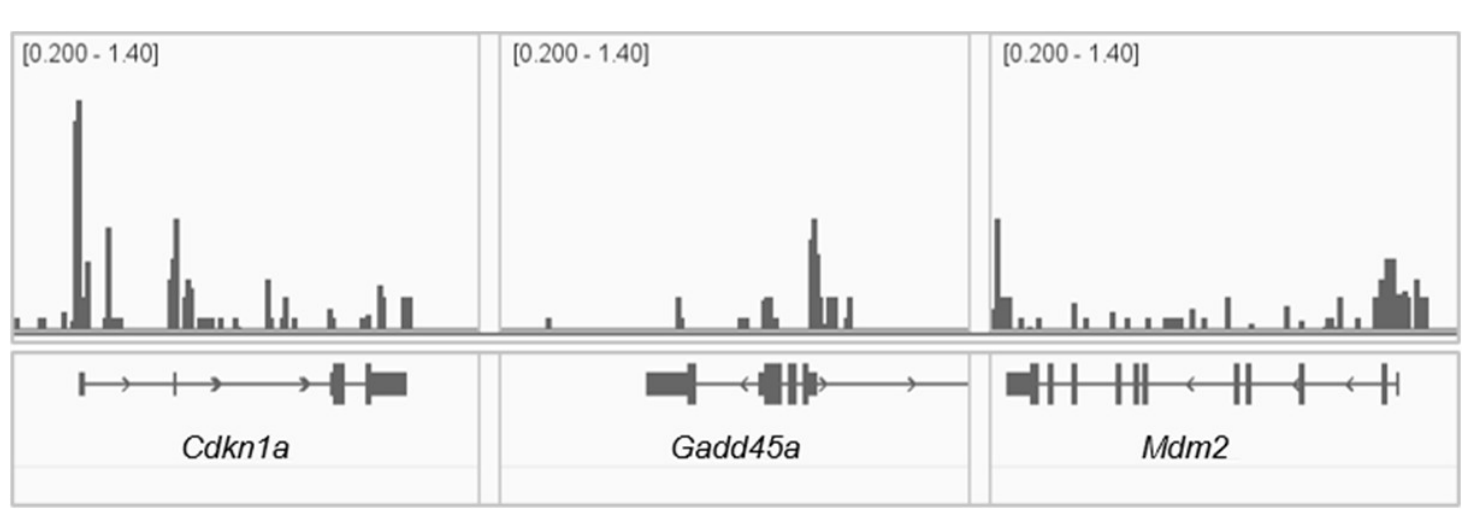

Representative BBX ChIP-seq tracks at the Cdkn1a (p21), Gadd45a, and Mdm2 loci in GSM3019272. Tracks

are shown at the same y-axis scale. BBX enrichment at the Gadd45a and Mdm2 loci was weaker than that observed at the Cdkn1a (p21) locus and did not show comparable promoter-proximal enrichment.

18. The efficiency of the knockdown vector for p21 should be shown.

> As suggested by the referee, we have included qPCR data validating the knockdown efficiency of the p21-targeting vector in Fig. EV4A. We also validated the knockdown efficiency of the RB-targeting vector used in the rescue experiments, and these results are shown in the same panel.

19. In the experiments with p53DN, p21, and RB knockdown, there is a marked reduction of cells accumulating in the IZ. What is the relationship between these IZ-accumulating cells, cell cycle exit, and senescence?

> We thank the referee for raising this important point. We interpret the IZ-accumulating cells as BBX-depleted cells in which aberrant activation of the p53–p21 pathway promotes both cell-cycle exit and senescence-associated cellular changes. Increased cell-cycle exit can explain, at least in part, the reduced retention of cells in the proliferative VZ/SVZ. However, cell-cycle exit alone would not readily explain the formation of large abnormal clusters in the IZ. If BBX knockdown simply promoted cell-cycle exit, electroporated cells would be expected to continue migrating toward the cortical plate after leaving the progenitor zone.

> In support of this interpretation, we showed in the original manuscript that cells within the IZ clusters express high levels of p21. In the revised manuscript, we further added γH2AX staining and found that this DNA damage/senescence-associated marker was strongly induced in the clustered cells (Fig. 5G). Thus, these cells appear not only to have exited the

cell cycle but also to exhibit a senescence-associated DNA damage response and a functionally compromised cellular state. Such a state may disrupt the transcriptional, translational, metabolic, and cytoskeletal programs required for neuronal migration, polarization, and neurite remodeling, thereby contributing to abnormal accumulation in the IZ.

> Consistent with this model, suppression of p53 activity or p21 expression markedly reduced IZ cluster formation, indicating that the p53–p21 pathway is required for this phenotype. In addition, RB co-depletion also reduced IZ accumulation, supporting the idea that p21/RB-associated senescence-related changes contribute to the abnormal clustering of BBX-depleted cells in the IZ.

20. Since knockdown experiments were performed in vivo, it would be important to examine γH2AX staining in these samples to support the conclusions.

> We agree with the referee that γH2AX staining in the in vivo knockdown samples is important for supporting our conclusion. We therefore performed γH2AX staining after in utero electroporation of the shBBX construct and added the results to Fig. 5G and H. The revised data show a marked increase in γH2AX signals in shBBX-electroporated cortical tissues, including the abnormal IZ cell clusters and cells located in the CP region. Because we showed in the original manuscript that the IZ clusters were composed largely of DCX^{+} immature neurons, these findings suggest that BBX-depleted neuronal populations accumulating in the IZ exhibit a senescence-associated DNA damage response in vivo. We have revised the Results section accordingly.

21. In Fig. 6A-B, please show SOX2 immunostaining on tissue sections rather than relying

on Western blotting alone, since this would provide more direct evidence.

> Following the referee's suggestion, we performed SOX2 immunostaining on tissue sections after in utero electroporation. Consistent with the Western blot results, BBX overexpression increased the percentage of $SOX2^{+}$ electroporated cells in the VZ/SVZ, and this increase was reduced by co-expression of p21. Conversely, the reduction in $SOX2^{+}$ electroporated cells caused by BBX knockdown was rescued by p21 co-knockdown using shp21. These results have been added to Fig. EV4B and EV4C and provide more direct in vivo evidence that BBX regulates $SOX2^{+}$ progenitor populations in a p21-dependent manner.

References

1. Ankam S, Teo BKK, Pohan G, Ho SWL, Lim CK, Yim EKF (2018) Front Bioeng Biotech 6: 69
2. Boi L, Johansson Y, Tonini R, Moratalla R, Fisone G, Silberberg G (2024) eLife 12: RP90278
3. Siegenthaler JA, Miller MW (2005) J Neurosci 25: 8627–36

1st Revision - Editorial Decision **31st Aug 2026**

Dear Prof. Yoon,

Thank you for the submission of your revised manuscript. We have now received the enclosed reports from the referees and I am happy to say that all support its publication now. Referee 1 still has a few more minor suggestions that I would like you to incorporate before we can proceed with the official acceptance of your manuscript.

A few editorial requests will also need to be addressed:

- The Disclosure and Competing Interest Statement needs to be placed after the Acknowledgments.

- The author credits need to be removed from the ms file. All credits need to be entered during online ms submission.

- A callout is missing for Fig. 1B, please add.

- For the 2 EV tables provided, their legends need to be removed from the ms file and provided in each Excel file; Table EV2 is a dataset and needs to be updated to Dataset EV1 in all places (the legend can be provided as a separate sheet/tab). The note to the editor about the two tables in the ms should deleted. Datasets can have several sheets, so all is fine.

- The SD for the main figures need to be uploaded as one folder per figure. The SD for all EV figures can be combined into one folder.

- Please add the specific URLs for the GSE297615 and GSE337091 datasets to the data availability statement.

* Figure legends comments *

1. Please note that the exact p values are not provided in the legends of figures 1B-L; 2A-H; 4D, F, G, H, I, J; 5B, C, D, E, F, G; 6A-G; EV1 A, B, C, D, E, F, G, I; EV2 A, B, C, D, E, G; EV3 C, D, E; EV4 A. Please provide exact values as reasonable.
2. Please indicate the statistical test used for data analysis in the legends of figures 3A, B, C; EV5 D
3. Please note that information related to n is missing in the legend of figure 3B
4. Please note that the scale bar needs to be defined for figures 2B, C; EV2 E, F.
5. Please note that the white dashed lines are not defined in the legend of figure 2B-F; 4M, 6C, D, G; EV2 A, B, C, E, F; EV3 C, EV4 B, C. This needs to be rectified.
6. Please note that the white borders are not defined in the legend of figure 2A-F; 4M, EV2 A, B, C, E, F; EV3 C, EV4 B, C. This needs to be rectified.

I would like to suggest a minor change to the title. Do you agree with this:

BBX maintains mammalian brain development by repressing p53-mediated p21 expression

Please also add "mouse" to the abstract.

EMBO press papers are accompanied online by A) a short (1-2 sentences) summary of the findings and their significance, B) 2-3 bullet points highlighting key results and C) a synopsis image that is exactly 550 pixels wide and 300 pixels high. The synopsis image should provide a sketch of the major findings, like a graphical abstract. Please note that text needs to be readable at the final size. Please send us this information along with the final manuscript.

I look forward to seeing a final version of your manuscript as soon as possible.

Kind regards,
Esther

Esther Schnapp, PhD
Senior Editor
EMBO reports

Referee #1:

The authors have address most of the initial comments (apart from generating a mouse model) proposed by the reviewers.

2 minor points:

1) In EV2 A' the arrows does not point to Tbr2+ cells

2) In Fig4B in the text it is written that the authors used NSCs for co-IP, in the figure legend it says E13.5 cortical lysates . This should be corrected and clarified.

Referee #2:

I agree with the publication of this paper in current form.

Referee #3:

All this reviewer's concerns have been satisfactorily addressed in this revision. This reviewer recommend acceptance without further changes.

The authors have addressed all minor editorial requests.

Prof. Keejung Yoon
Sungkyunkwan University
Department of Integrative Biotechnology
Office 207 Bldg 62
2066, Seobu-ro, Jangan-gu
Suwon, Gyeonggi-do 16419
Korea, Republic of

Dear Prof. Yoon,

I am very pleased to accept your manuscript for publication in the next available issue of EMBO reports. Thank you for your contribution to our journal.

Your manuscript will be processed for publication by EMBO Press. It will be copy edited and you will receive page proofs prior to publication. Please note that you will be contacted by Springer Nature Author Services to complete licensing and payment information.

You may qualify for financial assistance for your publication charges - either via a Springer Nature fully open access agreement or an EMBO initiative. Check your eligibility: https://link.springer.com/journal/44319/how-to-publish-with-us

Should you be planning a Press Release on your article, please notify the production team via this form in order to coordinate publication and release dates: https://forms.cloud.microsoft/e/eH4A8JVP6g

If you have any questions, please do not hesitate to contact the Editorial Office. Thank you for your contribution to EMBO Reports.

Best regards,
Esther

Esther Schnapp, PhD
Senior Editor
EMBO reports

>>> Please note that it is EMBO Reports policy for the transcript of the editorial process (containing referee reports and your response letter) to be published as an online supplement to each paper. If you do NOT want this, you will need to inform the Editorial Office via email immediately. More information is available here: https://link.springer.com/partners/embo-press/editorial-policies#Peer%20review

EMBO Press Author Checklist

Corresponding Author Name: Keejung Yoon	
Journal Submitted to: EMBO Reports	
Manuscript Number: EMBOR-2025-62413V3	

USEFUL LINKS FOR COMPLETING THIS FORM
The EMBO Journal - Author Guidelines
EMBO Reports - Author Guidelines
Molecular Systems Biology - Author Guidelines
EMBO Molecular Medicine - Author Guidelines

Reporting Checklist for Life Science Articles (updated January
This checklist is adapted from Materials Design Analysis Reporting (MDAR) Checklist for Authors. MDAR establishes a minimum set of requirements in transparent reporting in the life sciences (see Statement of Task: 10.31222/osf.io/9sm4x). Please follow the journal's guidelines in preparing your
Please note that a copy of this checklist will be published alongside your article.

Abridged guidelines for figures
1. Data
The data shown in figures should satisfy the following conditions:
- the data were obtained and processed according to the field's best practice and are presented to reflect the results of the experiments in an accurate and unbiased manner.
- ideally, figure panels should include only measurements that are directly comparable to each other and obtained with the same assay.
- plots include clearly labeled error bars for independent experiments and sample sizes. Unless justified, error bars should not be shown for technical
- if n<5, the individual data points from each experiment should be plotted. Any statistical test employed should be justified.
- Source Data should be included to report the data underlying figures according to the guidelines set out in the authorship guidelines on Data

2. Captions
Each figure caption should contain the following information, for each panel where they are relevant:
- a specification of the experimental system investigated (eg cell line, species name).
- the assay(s) and method(s) used to carry out the reported observations and measurements.
- an explicit mention of the biological and chemical entity(ies) that are being measured.
- an explicit mention of the biological and chemical entity(ies) that are altered/varied/perturbed in a controlled manner.
- the exact sample size (n) for each experimental group/condition, given as a number, not a range;
- a description of the sample collection allowing the reader to understand whether the samples represent technical or biological replicates (including how many animals, litters, cultures, etc.).
- a statement of how many times the experiment shown was independently replicated in the laboratory.
- definitions of statistical methods and measures:
  - common tests, such as t-test (please specify whether paired vs. unpaired), simple χ2 tests, Wilcoxon and Mann-Whitney tests, can be unambiguously identified by name only, but more complex techniques should be described in the methods section;
  - are tests one-sided or two-sided?
  - are there adjustments for multiple comparisons?
  - exact statistical test results, e.g., P values = x but not P values < x;
  - definition of 'center values' as median or average;
  - definition of error bars as s.d. or s.e.m.

Please complete ALL of the questions below.
Select "Not Applicable" only when the requested information is not relevant for your study.

Materials

Newly Created Materials	Information included in the manuscript?	In which section is the information available? (Reagents and Tools Table, Materials and Methods, Figures, Data Availability Section)
New materials and reagents need to be available; do any restrictions apply?	Yes	Methods

Antibodies	Information included in the manuscript?	In which section is the information available? (Reagents and Tools Table, Materials and Methods, Figures, Data Availability Section)
For **antibodies** provide the following information:<br>- Commercial antibodies: RRID (if possible) or supplier name, catalogue number and or/clone number<br>- Non-commercial: RRID or citation	Yes	Reagents and Tools Table, Methods

DNA and RNA sequences	Information included in the manuscript?	In which section is the information available? (Reagents and Tools Table, Materials and Methods, Figures, Data Availability Section)
**Short novel DNA or RNA including primers, probes**: provide the sequences.	Yes	Reagents and Tools Table

Cell materials	Information included in the manuscript?	In which section is the information available? (Reagents and Tools Table, Materials and Methods, Figures, Data Availability Section)
**Cell lines:** Provide species information, strain. Provide accession number in repository **OR** supplier name, catalog number, clone number, and/**OR** RRID.	Yes	Methods
**Primary cultures:** Provide species, strain, sex of origin, genetic modification status.	Yes	Methods
Report if the cell lines were recently **authenticated** (e.g., by STR profiling) and tested for mycoplasma contamination.	Yes	Methods

Experimental animals	Information included in the manuscript?	In which section is the information available? (Reagents and Tools Table, Materials and Methods, Figures, Data Availability Section)
**Laboratory animals or Model organisms:** Provide species, strain, sex, age, genetic modification status. Provide accession number in repository **OR** supplier name, catalog number, clone number, **OR** RRID.	Yes	Methods
**Animal observed in or captured from the field:** Provide species, sex, and age where possible.	Not Applicable	
Please detail **housing and husbandry conditions**.	Yes	Methods

Plants and microbes	Information included in the manuscript?	In which section is the information available? (Reagents and Tools Table, Materials and Methods, Figures, Data Availability Section)
**Plants:** provide species and strain, ecotype and cultivar where relevant, unique accession number if available, and source (including location for collected wild specimens).	Not Applicable	
**Microbes:** provide species and strain, unique accession number if available, and source.	Yes	Methods

Human research participants	Information included in the manuscript?	In which section is the information available? (Reagents and Tools Table, Materials and Methods, Figures, Data Availability Section)
If collected and within the bounds of privacy constraints report on age, sex and gender or ethnicity for all study participants.	Not Applicable	

Core facilities	Information included in the manuscript?	In which section is the information available? (Reagents and Tools Table, Materials and Methods, Figures, Data Availability Section)
If your work benefited from core facilities, was their service mentioned in the acknowledgments section?	Not Applicable	

Design

Study protocol	Information included in the manuscript?	In which section is the information available? (Reagents and Tools Table, Materials and Methods, Figures, Data Availability Section)
If study protocol has been **pre-registered, provide DOI in the manuscript**. For clinical trials, provide the trial registration number **OR** cite DOI.	Not Applicable	
Report the **clinical trial registration number** (at ClinicalTrials.gov or equivalent), where applicable.	Not Applicable	

Laboratory protocol	Information included in the manuscript?	In which section is the information available? (Reagents and Tools Table, Materials and Methods, Figures, Data Availability Section)
Provide DOI OR other citation details if **external detailed step-by-step protocols** are available.	Not Applicable	

Experimental study design and statistics	Information included in the manuscript?	In which section is the information available? (Reagents and Tools Table, Materials and Methods, Figures, Data Availability Section)
Include a statement about **sample size** estimate even if no statistical methods were used.	Yes	Methods
Were any steps taken to minimize the effects of subjective bias when allocating animals/samples to treatment (e.g. **randomization procedure**)? If yes, have they been described?	Yes	Methods
Include a statement about **blinding** even if no blinding was done.	Yes	Methods
Describe **inclusion/exclusion criteria** if samples or animals were excluded from the analysis. Were the criteria pre-established?<br><br>If sample or data points were omitted from analysis, report if this was due to attrition or intentional exclusion and provide justification.	Yes	Methods
For every figure, are **statistical tests** justified as appropriate? Do the data meet the assumptions of the tests (e.g., normal distribution)? Describe any methods used to assess it. Is there an estimate of variation within each group of data? Is the variance similar between the groups that are being statistically compared?	Yes	Methods, Figure Legends

Sample definition and in-laboratory replication	Information included in the manuscript?	In which section is the information available? (Reagents and Tools Table, Materials and Methods, Figures, Data Availability Section)
In the figure legends: state number of times the experiment was **replicated** in laboratory.	Yes	Figure Legends
In the figure legends: define whether data describe **technical or biological replicates**.	Yes	Figure Legends

Ethics

Ethics	Information included in the manuscript?	In which section is the information available? (Reagents and Tools Table, Materials and Methods, Figures, Data Availability Section)
Studies involving **human participants**: State details of **authority granting ethics approval** (IRB or equivalent committee(s), provide reference number for approval.	Not Applicable	
Studies involving **human participants**: Include a statement confirming that **informed consent** was obtained from all subjects and that the experiments conformed to the principles set out in the WMA Declaration of Helsinki and the Department of Health and Human Services Belmont Report.	Not Applicable	
Studies involving **human participants:** For publication of **patient photos**, include a statement confirming that consent to publish was obtained.	Not Applicable	
Studies involving experimental **animals**: State details of **authority granting ethics approval** (IRB or equivalent committee(s), provide reference number for approval. Include a statement of compliance with ethical regulations.	Yes	Methods
Studies involving **specimen and field samples:** State if relevant **permits** obtained, provide details of authority approving study; if none were required, explain why.	Not Applicable	Not applicable, as this study did not involve field-collected specimens or field samples and therefore did not require collection permits.

Dual Use Research of Concern (DURC)	Information included in the manuscript?	In which section is the information available? (Reagents and Tools Table, Materials and Methods, Figures, Data Availability Section)
Could your study fall under dual use research restrictions? Please check biosecurity documents and list of **select agents and toxins** (CDC): https://www.selectagents.gov/sat/list.htm	Not Applicable	
If you used a select agent, is the security level of the lab appropriate and reported in the manuscript?	Not Applicable	
If a study is subject to dual use research of concern regulations, is the name of the **authority granting approval and reference number** for the regulatory approval provided in the manuscript?	Not Applicable	

Reporting

The MDAR framework recommends adoption of discipline-specific guidelines, established and endorsed through community initiatives. Journals have their own policy about requiring specific guidelines and recommendations to complement MDAR.

Adherence to community standards	Information included in the manuscript?	In which section is the information available? (Reagents and Tools Table, Materials and Methods, Figures, Data Availability Section)
State if relevant guidelines or checklists (e.g., **ICMJE, MIBBI, ARRIVE, PRISMA**) have been followed or provided.	Not Applicable	
For **tumor marker prognostic studies,** we recommend that you follow the **REMARK** reporting guidelines (see link list at top right). See author guidelines, under 'Reporting Guidelines'. Please confirm you have followed these guidelines.	Not Applicable	
For **phase II and III randomized controlled trials**, please refer to the **CONSORT** flow diagram (see link list at top right) and submit the CONSORT checklist (see link list at top right) with your submission. See author guidelines, under 'Reporting Guidelines'. Please confirm you have submitted this list.	Not Applicable	

Data Availability

Data availability	Information included in the manuscript?	In which section is the information available? (Reagents and Tools Table, Materials and Methods, Figures, Data Availability Section)
Have **primary datasets** been deposited according to the journal's guidelines (see 'Data Deposition' section) and the respective accession numbers provided in the Data Availability Section?	Yes	Methods
Were **human clinical and genomic datasets** deposited in a public access-controlled repository in accordance to ethical obligations to the patients and to the applicable consent agreement?	Not Applicable	
Are **computational models** that are central and integral to a study available without restrictions in a machine-readable form? Were the relevant accession numbers or links provided?	Not Applicable	
If publicly available data were reused, provide the respective **data citations in the reference list**.	Yes	References
